# Discovery of 5-Hydroxy-1,4-naphthoquinone (Juglone) Derivatives as Dual Effective Agents Targeting Platelet-Cancer Interplay through Protein Disulfide Isomerase Inhibition

**DOI:** 10.1021/acs.jmedchem.3c02107

**Published:** 2024-02-21

**Authors:** Yu-Pu Juang, Ju-Ying Tsai, Wan-Lan Gu, Hui-Ching Hsu, Chao-Lung Lin, Chin-Chung Wu, Pi-Hui Liang

**Affiliations:** † School of Pharmacy, College of Medicine, National Taiwan University, Taipei 100, Taiwan; ‡ Graduate Institute of Natural Product, Kaohsiung Medical University, Kaohsiung 807, Taiwan; § The Genomics Research Center, Academia Sinica, Taipei 128, Taiwan

## Abstract

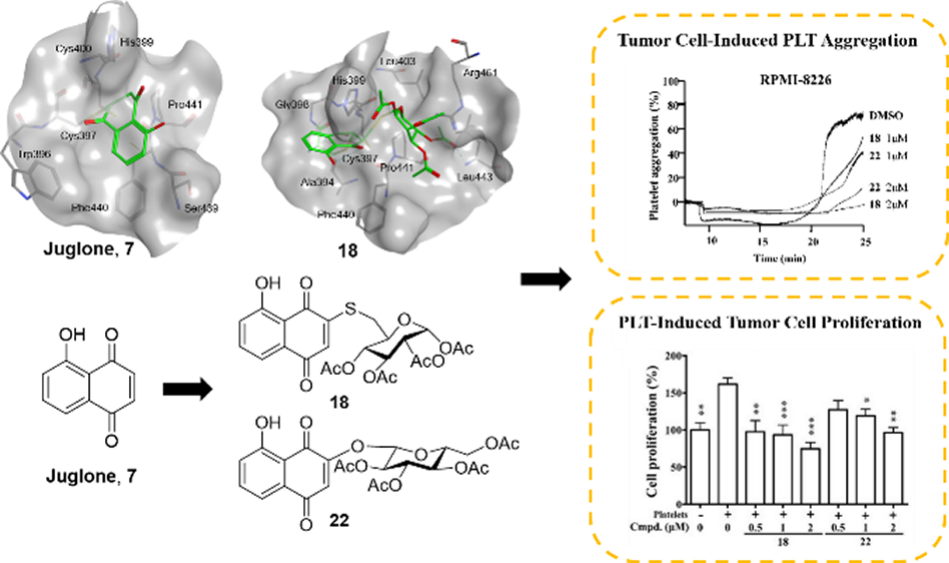

In this study, a series of 2- and/or 3-substituted juglone derivatives were designed and synthesized. Among them, **9**, **18**, **22**, **30**, and **31** showed stronger inhibition activity against cell surface PDI or recombinant PDI and higher inhibitory effects on U46619- and/or collagen-induced platelet aggregation than juglone. The glycosylated derivatives **18** and **22** showed improved selectivity for inhibiting the proliferation of multiple myeloma RPMI 8226 cells, and the IC_50_ values reached 61 and 48 nM, respectively, in a 72 h cell viability test. In addition, **18** and **22** were able to prevent tumor cell-induced platelet aggregation and platelet-enhanced tumor cell proliferation. The molecular docking showed the amino acid residues Gln243, Phe440, and Leu443 are important for the compound–protein interaction. Our results reveal the potential of juglone derivatives to serve as novel antiplatelet and anticancer dual agents, which are available to interrupt platelet–cancer interplay through covalent binding to PDI catalytic active site.

## Introduction

The interaction between platelets and cancer cells plays an important role in cancer progression and thrombotic complications.^1^ Cancer-associated thrombosis, a common cause of death in patients with malignancy, is related to thrombocytosis and the ability of cancer cells to activate coagulation cascades and platelets.^2−4^ On the other hand, platelets form aggregates with cancer cells that are known to increase metastatic potential by impeding immune attack and enhancing cancer cell survival in the circulation.^5^ Moreover, cancer-activated platelets secret the δ-granule components ADP and ATP, which contribute to cancer cell extravasation,^6^ and the α-granule components, such as platelet-derived growth factor (PDGF), epidermal growth factor, and vascular endothelial growth factor, which increase cancer cell growth during metastasis.^7^ Therefore, targeting the platelet–cancer interaction is a promising strategy for cancer treatment.

PDI is the prototype of the PDI family that mainly locates in endoplasmic reticulum (ER), where it facilitates oxidative protein folding via disulfide bond formation and isomerization.^8^ In addition to ER, PDI is also expressed at the cell surface where it tends to act as a thiol reductase and is implicated in various pathophysiological processes, such as thrombosis, cancer, and inflammatory responses.^9,10^ For example, cell surface PDI contributes to platelet aggregation and blood coagulation.^11^ Studies using platelet-specific PDI-deficient mice have revealed that PDI is essential for thrombus propagation but not for hemostasis.^12,13^ In cancers, ER-resident PDI is involved in cancer cell proliferation and survival, while cell surface PDI plays an important role in regulating the adhesion and migration of cancer cells.^14^ Moreover, PDI overexpression is frequently observed in cancers and is correlated with worse prognosis.^15,16^

**Figure 1:**
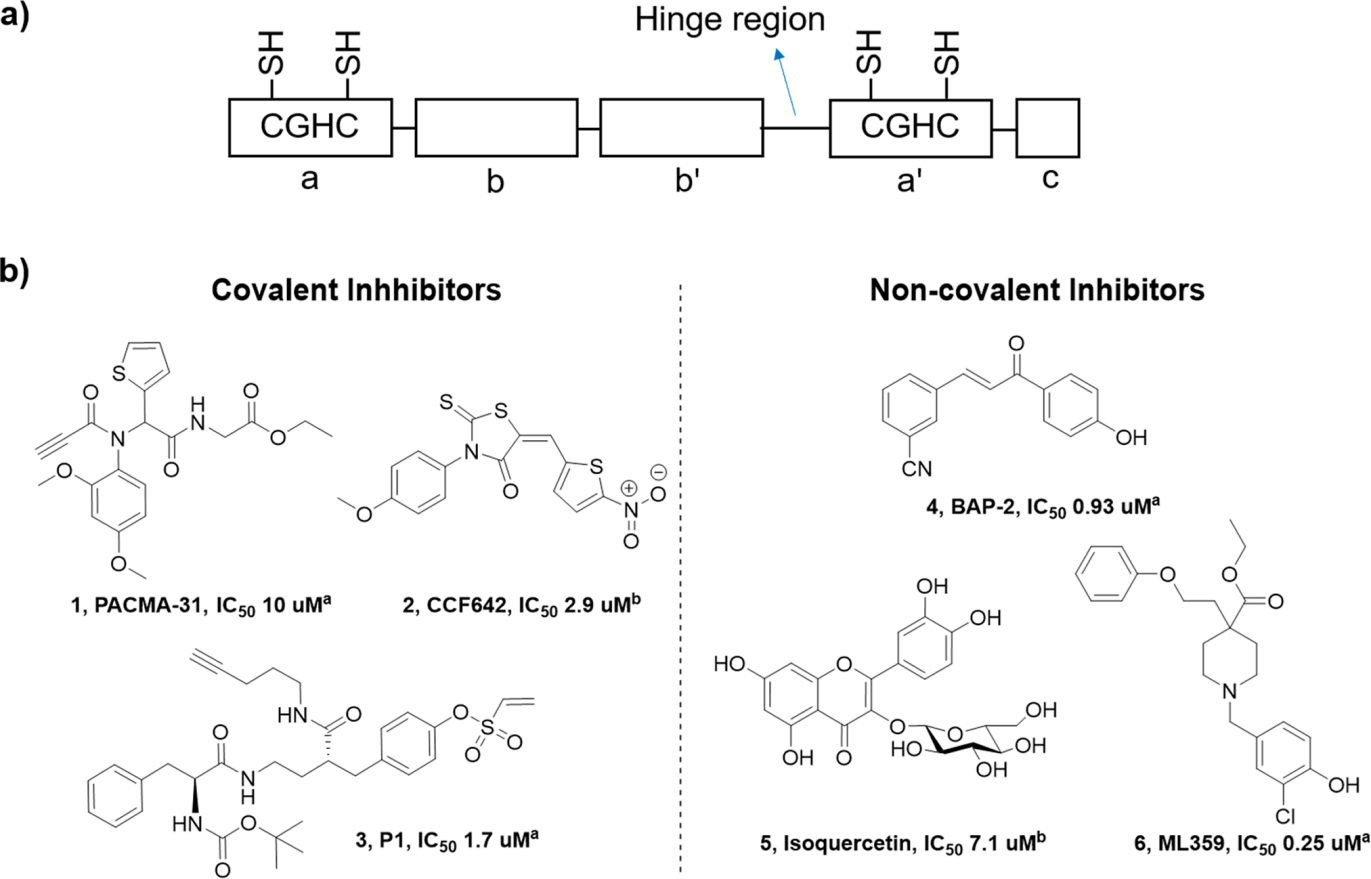
PDI protein domain and reported PDI inhibitors. (a) PDI protein is consisted with a, b, b′, a′, and c domain, the catalytic cysteine residue is existed in a and a′ domain. (b) Structures and activity of PDI inhibitors. ^a^IC_50_ measured by the insulin-aggregation assay; ^b^IC_50_ measured by the glutathione disulfide (GSSG) fluorescence assay.

The reported PDI inhibitors can be categorized into two types, active site covalent inhibitors targeting catalytic cysteine in the a′ domain and noncovalent inhibitors targeting substrate binding site in the b′ domain of the PDI (Figure 1a).^11,17−22^ The covalent inhibitors of PDI are Michael acceptor electrophiles, which react with the active site cysteine residues of PDI through thiol-Michael addition. PACMA-31 (**1**) and CCF642 (**2**) have shown cytotoxic effects against ovarian cancer and multiple myeloma through inhibition of PDI and induction of ER stress.^17,18,23^ Based on the previously reported covalent inhibitor of PDI, the P1 (**3**) was synthesized and showed enhanced PDI inhibition activity.^19^ On the other hand, several noncovalent inhibitors were developed, such as BAP-2 (**4**), isoquercetin (**5**), and ML359 (**6**), exhibiting the inhibition mechanism of blocking the substrate binding site in the b′ domain.^20,22^ The flavonoid glycoside isoquercetin (**5**, Figure 1b) has been reported to exhibit antiplatelet activity,^11^ and demonstrated efficacy in preventing platelet activation and platelet-dependent thrombin generation in a phase II/III cancer clinical trial with no serious adverse events.^24^ Because cancer-induced platelet activation and hypercoagulability are frequently observed in cancer patients,^25^ these results reveal a potential role for PDI inhibitors in the treatment of cancer and thrombotic complications by blocking the platelet–cancer interaction.^26,27^

**Figure 2:**
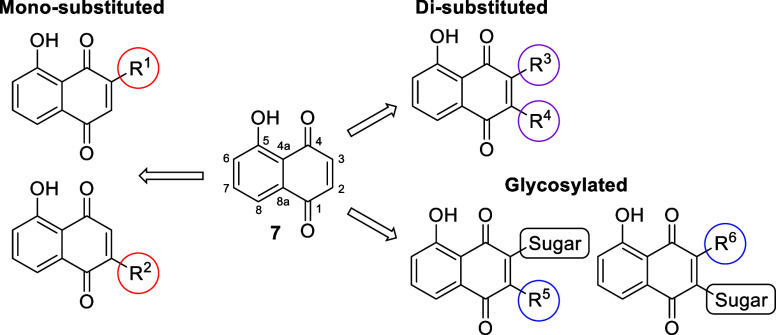
Three design strategies to generate JUG derivatives.

Juglone (JUG, **7**, Figure 2), 5-hydroxy-1,4-naphthoquinone, is an allelochemical found in walnut plants such as *Juglans regia (J. regia)*.^28^ Walnut extracts are widely used in folk medicine for treating arthritis, stomach aches, skin disorders, and infectious diseases.^29,30^ Besides, JUG has been used as a natural dye and colorant in cosmetics and foods despite its potentially hazardous effects.^31^ Previous studies have shown the anticancer, anti-inflammatory, antidiabetic, and antiviral effects of JUG,^32,33^ and a number of JUG derivatives have been synthesized and biologically evaluated.^34−36^ Recently, we have reported that JUG exhibited antiplatelet effects which were associated with inhibition of platelet surface PDI activity.^37^

The chemical structure of JUG contains a quinone-type Michael acceptor that is able to covalently bind to the cysteine thiols of cellular proteins as well as glutathione (GSH), contributing to both pharmacological effects and toxicity.^38,39^ Chemical modifications of Michael acceptors have been reported to be a useful method for modulation of their thiol reactivity and thus bioactivities and/or toxicity.^40^ In this study, a panel of 2- and/or 3-substituted JUG derivatives were synthesized and evaluated for their biological effects and cytotoxicity. Three strategies were applied for the derivatization on the Michael acceptor sites (Figure 2): (1) monosubstitution; (2) disubstitution; and (3) glycosylation. The third is inspired by nature, since in the plant tissue, JUG is stored in a nontoxic glycosylated form, hydrojuglone glucoside, before being released into the soil.^41,42^ Moreover, the natural PDI inhibitors isoquercetin and rutin also contain glycosidic linkages, which are crucial for their PDI-inhibitory activity and may restrict cell permeability, thereby sparing intracellular PDI and reducing cytotoxicity.^11^

## Results and Discussion

### Synthesis of JUG Derivatives

**Scheme 1:**
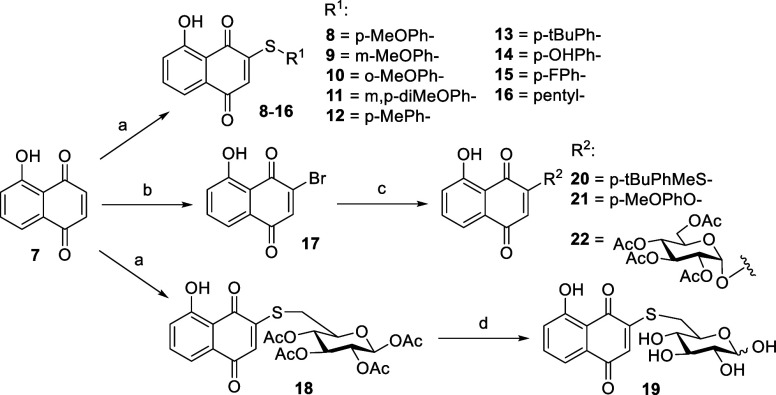
Synthesis of Mono-Substituted Derivatives **8–22** Reagents and conditions: (a) R-SH, ethanol, −20 or 0 °C; (b) Br_2_, acetic acid, CHCl_3_, 0 °C; (c) R-SH or R–OH, K_2_CO_3_, *N*,*N*-dimethylformamide (DMF); and (d) NaOMe, methanol, 0 °C.

The thioether-type JUG derivatives were synthesized via nucleophilic addition with a series of thiol nucleophiles (Scheme 1).^43^ JUG was suspended in ethanol under the N_2_ atmosphere at −20 °C followed by dropwise addition of thiol nucleophile suspended or dissolved in ethanol to produce compound **8**–**16**.^44^ Furthermore, 1,2,3,4-tetra-*O*-acetyl-6-desoxy-6-thio-β-d-glucopyranose in ethanol was added to JUG suspension to produce glycosylated JUG derivatives **18**. The acetyl groups on compound **18** were removed by NaOMe to give compound **19** as an α/β anomeric mixture.^45,46^ Due to low nucleophilicity of the hydroxyl group, the bromide was first added to JUG for the synthesis of compound **20**, **21**, and **22**. To a solution of JUG in CHCl_3_ was added bromine, followed by the addition of acetic acid and ethanol, refluxed for 2 h to give compound **17**. Next, compounds **20**, **21,** and **22** were synthesized through nucleophilic substitution with hydroxyl-bearing nucleophiles.

**Scheme 2:**
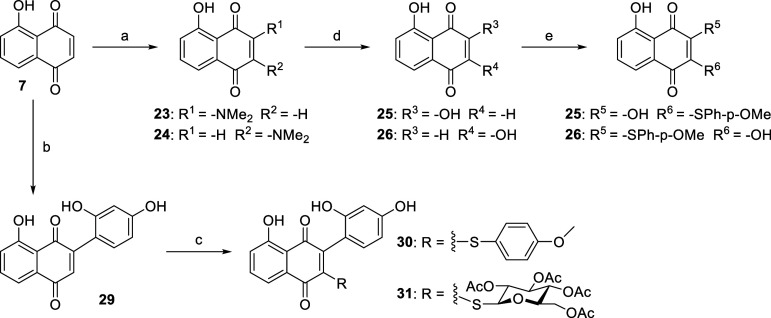
Synthesis of Disubstituted Derivatives **23**–**31** Reagents and conditions: (a) dimethylamine (2 M in THF), H_2_O; (b) resorcinol, acetic acid, 2 M H_2_SO_4(aq)_; (c) 4-methoxybenzenethiol or 2,3,4,6-tetra-*O*-acetyl-1-thio-d-glucopyranose, ethanol, −20 °C; (d) 10% HCl_(aq)_, 1,4-dioxane, reflux; and (e) 4-methoxybenzenethiol, ethanol, −50 °C.

To evaluate the influence of blocking Michael acceptor, disubstituted JUG derivatives were synthesized. To a suspension of JUG in H_2_O was added dimethylamine, stirred for 2 h to give **23** and **24** (Scheme 2). Compounds **23** and **24** were hydrolyzed with 10% HCl_(aq)_ to give hydroxylated JUG **25** and **26**.^47^ Disubstituted derivatives **27** and **28** were synthesized from compounds **25** and **26** via the nucleophilic addition with 4-methoxybenzenethiol. To mimic the structure of isoquercetin (**5**), which inhibited plasma PDI activity and diminished platelet-dependent thrombin generation in a phase II/III clinical trial,^24^ resorcinol was coupled to a suspension of JUG in acetic acid and H_2_SO_4_ (2M) to give compound **29** via the modified Michael addition. Compounds **30** and **31** were synthesized from compound **29** via addition with 4-methoxybenzenethiol and 2,3,4,6-tetra-*O*-acetyl-1-thio-d-glucopyranose.^48^ To the best of our knowledge, the synthesis and structure elucidation of compounds **10**, **11**, **13**, **14**, **15**, **16**, **18**, **20**, **27**, **28**, **30**, and **31** were reported first.

### JUG Derivatives Show Inhibition Activity against Surface and Recombinant PDI

We first examined the inhibitory effects of the JUG derivatives on cell surface PDI reductase activity by using intact human platelets in which PDI is the major member of the PDI family at the platelet surface.^49^ In this assay, a nonfluorescent substrate, dieosin glutathione disulfide (Di-E-GSSG), was reduced into a fluorescent product eosin-glutathione (E-GSH) by platelet surface PDI in the presence of 5 μM dithiothreitol (DTT). As shown in Table 1, the JUG derivatives, except **23**–**28**, inhibited platelet surface PDI-inhibitory activity. Among them, the potencies of **18**, **22**, **29**, **30**, and **31** (IC_50_ = 0.42–0.62 μM) were comparable or stronger than that of JUG (IC_50_ = 0.63 μM). The active compounds were further investigated for their effects on purified human recombinant PDI (rPDI). Consistently, these compounds also inhibited PDI activity in the pure enzyme system, with **9**, **22**, **29**, and **31** being the most potent (IC_50_ values of 0.63–0.77 μM compared with 1.10 μM for JUG). The monosubstituted JUG derivatives **8**–**16** and **20**, in which the Michael acceptor was blocked by a thiophene or thiopentyl, exhibited a slightly less inhibitory activity on both cell surface PDI and rPDI compared with JUG, with the exception of **9**, which showed more potent inhibition on rPDI than JUG did. Of notice, glucosylation of JUG (**18**, **19**, **22**, and **31**) markedly reduced the membrane permeability (Table S1) while preserving or even enhancing the inhibitory activity against cell surface PDI and rPDI. A comparison of **18** and **19** showed that per-acetylation of the glucose moiety provided better PDI-inhibitory activity. In addition, the similar potency of ether-type derivatives to their thio-congeners (**8** and **21**; **18** and **22**) indicates that the S and O are interchangeable as a linker. The hydroxyl and dimethylamine substitutions of the Michael acceptor of JUG derivatives (**23**–**28**) led to the loss of PDI-inhibitory activity that might result from the resonance ability of hydroxyl and dimethylamine substitutions which prevented Michael's addition. On the other hand, the disubstituted JUG derivatives **30** and **31**, in which the Michael acceptor sites were blocked by a resorcinol moiety and by a thiophene or thio-glucose moiety, still exhibited potent anti-PDI activity. This suggests that **30** and **31** can inhibit PDI activity in a Michael addition-independent manner that is distinct from the other JUG derivatives, which might be related to noncovalent inhibition at the substrate binding site.

**Table 1:**
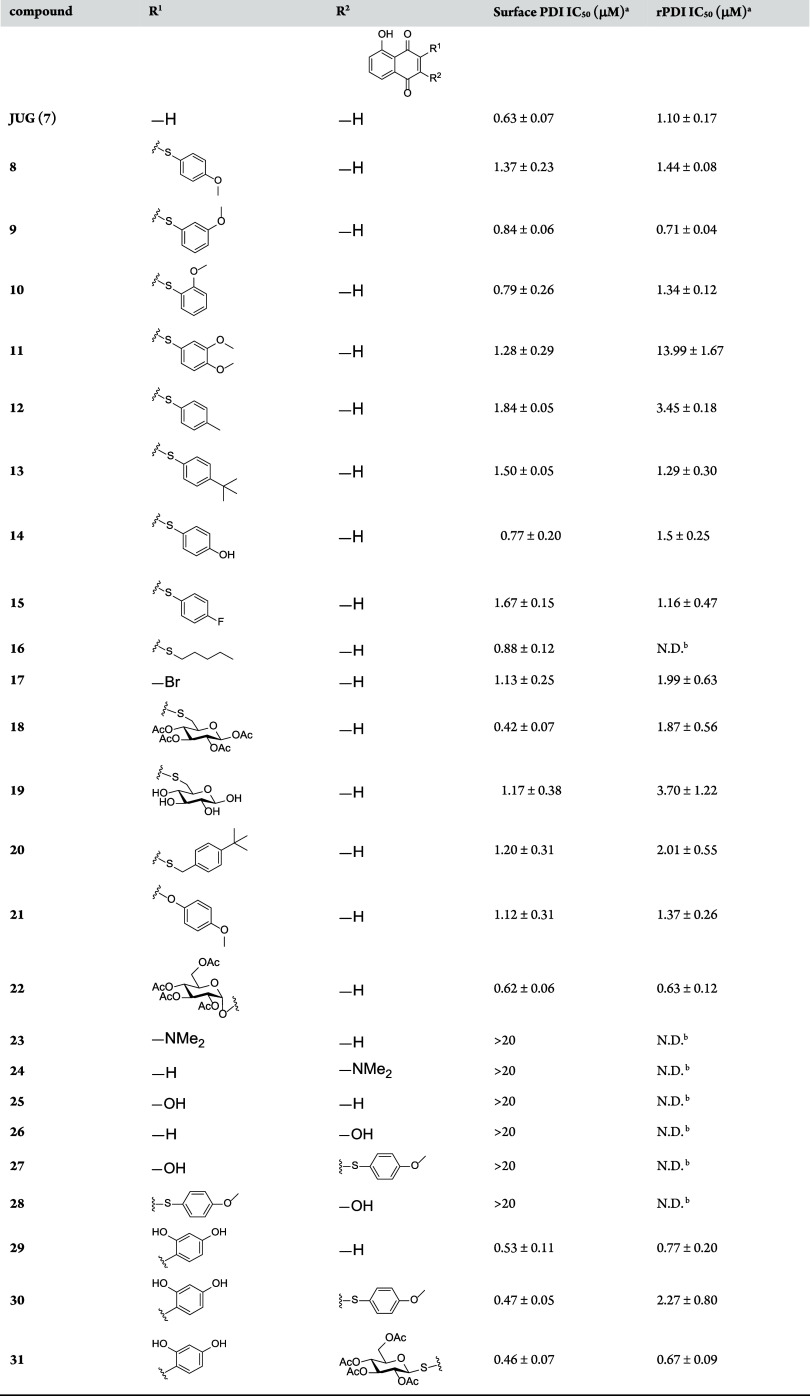
Inhibitory Activity of JUG Derivatives against Cell Surface PDI and rPDI

a PDI inhibition assay were determined using an artificial PDI substrate, Di-E-GSSG, and fluorometry.

b Not determined.

### JUG Derivatives Inhibit U46619 and Collagen-Induced Platelet Aggregation

The antiplatelet effects of the JUG derivatives were evaluated by measuring human platelet aggregation, which can be regulated by cell surface PDI. Platelet aggregation was induced with platelet activators U46619 (a mimetic of thromboxane A_2_) and collagen (binding to GPVI). As shown in Table 2, the JUG derivatives with PDI-inhibitory activity, except **19**, were also capable of preventing U46619- and collagen-induced platelet aggregation. In contrast, compounds **23**–**28** which did not inhibit platelet PDI activity also showed no inhibitory effects on platelet aggregation. Among the antiplatelet JUG derivatives, **9**, **18**, and **22**, which showed stronger inhibition against either cell surface PDI or recombinant PDI activity than JUG, also exhibited more potent inhibitory effects on U46619- and/or collagen-induced platelet aggregation. Interestingly, juglone derivatives showed better inhibition activity against collagen-induced platelet aggregation compared to U46619-induced except **29**, **30,** and **31**, indicating that the addition of resorcinol moiety might alter the mechanism of derivatives.

**Table 2:** Inhibitory Effects of JUG Derivatives on Platelet Aggregation

	platelet aggregation IC_50_ (μM)^a^			platelet aggregation IC_50_ (μM)^a^	
cmpd.	U46619 induced	collagen induced	**cmpd.**	U46619 induced	collagen induced
JUG (**7**)	4.45 ± 0.47	1.18 ± 0.14	**20**	6.32 ± 0.30	2.92 ± 0.77
**8**	1.52 ± 0.08	0.29 ± 0.06	**21**	1.35 ± 0.13	0.35 ± 0.03
**9**	1.60 ± 0.16	0.38 ± 0.13	**22**	1.54 ± 0.02	0.50 ± 0.08
**10**	2.66 ± 0.42	0.71 ± 0.03	**23**	>20	>20
**11**	2.43 ± 0.30	1.58 ± 0.88	**24**	>20	15.98 ± 2.11
**12**	1.82 ± 0.17	0.76 ± 0.02	**25**	>20	>20
**13**	1.85 ± 0.17	0.64 ± 0.05	**26**	>20	>20
**14**	1.30 ± 0.35	0.75 ± 0.14	**27**	13.68 ± 0.89	>20
**15**	1.88 ± 0.18	0.83 ± 0.11	**28**	>20	>20
**16**	7.13 ± 1.82	2.57 ± 0.50	**29**	4.61 ± 1.10	7.04 ± 1.71
**17**	1.76 ± 0.08	0.52 ± 0.21	**30**	1.37 ± 0.03	5.19 ± 1.22
**18**	0.95 ± 0.10	0.56 ± 0.17	**31**	3.54 ± 0.11	6.65 ± 1.74
**19**	>50	>50	aspirin^50^	>200	157.1 ± 5.4

a Platelet aggregation of washed human platelets was induced by U46619 (1 μM) or collagen (5 μg/mL).

### JUG Derivatives Show Selective Cytotoxicity against Cancer over Normal Cell Line

**Figure 3:**
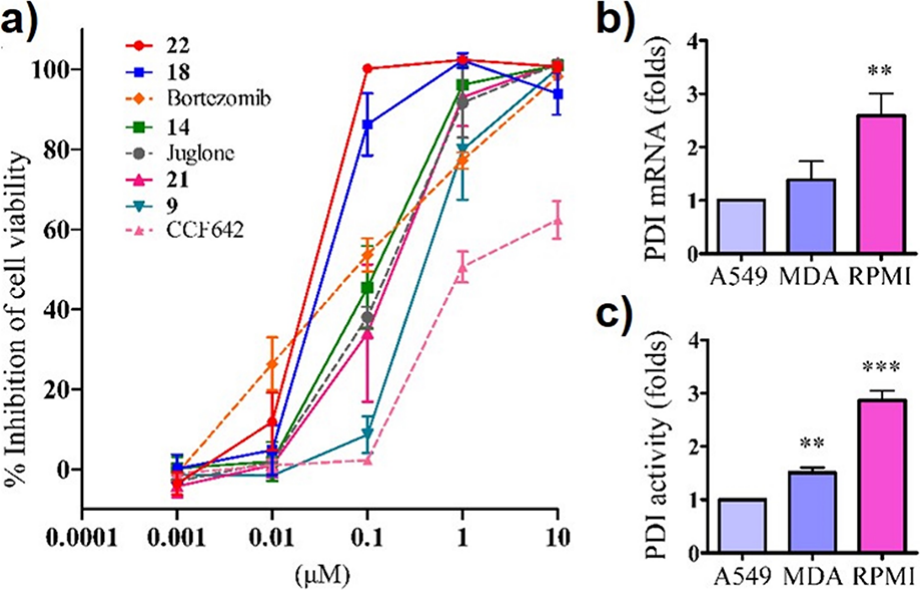
Inhibitory effects of JUG derivatives on multiple myeloma RPMI8226 cell proliferation. (a) RPMI8226 cells were incubated with JUG, JUG derivatives, the PDI inhibitor CCF642, and the proteasome inhibitor bortezomib for 72 h, the cell viability was determined with the resazurin assay (*n* = 4). (b) mRNA expression of PDI and (c) cell surface PDI activity in cancer cells were analyzed by using the RT-qPCR and Di-E-GSSG assay, respectively (*n* = 4). **P < 0.01; ***P < 0.001 as compared with A549 cells.

JUG has been investigated as a potential anticancer agent against breast cancer, lung cancer, prostate cancer, colorectal cancer, melanoma, and glioma.^33,51^ Here, we tested the cytotoxicity of the JUG derivatives against lung cancer A549, breast cancer MDA-MB-231, glioma U87, and multiple myeloma RPMI 8226 cell lines as well as human vascular endothelial EA.hy926 cells. As shown in Table 3, four cancer cell lines displayed a range of sensitivity to JUG (IC_50_ values from 3.95 to 11.87 μM) with RPMI8226 being the most susceptible. JUG was also toxic for endothelial cells with an IC_50_ value of 5.57 μM (selectivity index, S.I. = 1.4). Most of the substituted JUG derivatives exhibited weaker cytotoxicity than JUG against A549, MDA-MB-231, and U97 cancer cells as well as endothelial cells, with the exception of RPMI8226. Among them, **9**, **14**, **18**, **21**, and **22** had potent cytotoxicity against RPMI8226 with submicromolar IC_50_ values (0.57–0.77 μM) at 24 h of incubation. Furthermore, **18** and **21** exhibited S.I. larger than 10, indicating a sevenfold improvement of selectivity for cancer cells over normal cells compared to JUG. When the incubation period was extended to 72 h (Figure 3a), the potency of these compounds was further enhanced, with **18** and **22** being the most potent JUG derivatives against RPMI8226 (IC_50_ = 61 and 48 nM). Notably, regardless of incubation periods, the potency of **18** and **22** was superior than that of bortezomib (IC_50_ values of 5.24 μM and 80 nM at 24 and 72 h, respectively), which is a proteasome inhibitor used in the clinical treatment of multiple myeloma. Particularly, **18** and **22** displayed a better selectivity toward RPMI8226 than bortezomib, and the latter was very toxic for endothelial EA.hy926 cells. In regard of PDI, the cytotoxic PDI inhibitor CCF642 also displayed cytotoxicity toward RPMI8226 but was less potent than **18** and **22** did. Analysis of PDI mRNA expression in RPMI8226 and two other cancer cell lines (A549 and MDA-MB-231) revealed that RPMI8226 had the highest PDI expression (Figure 3b). Correspondingly, RPMI8226 also exhibited the highest cell surface PDI activity, indicating a crucial role of PDI in cell growth and survival of RPMI8226 (Figure 3c).

**Table 3:** Cell Viability of JUG Derivatives against Human Cancer Lines and Endothelial EAhy926 Cells^a^

cmpd.	A549 IC_50_ (μM)	MDA-MB-231 IC_50_ (μM)	U87 IC_50_ (μM)	RPMI8226 IC_50_ (μM)	EAhy926 IC_50_ (μM)	S.I.^b^ (EAhy926/RPMI8226)
JUG (**7**)	11.87 ± 0.60	6.15 ± 0.78	7.45 ± 0.33	3.95 ± 0.70	5.57 ± 0.02	1.4
**8**	14.87 ± 0.43	6.63 ± 0.93	16.77 ± 0.62	4.70 ± 0.08	13.87 ± 1.17	3.0
**9**	13.86 ± 0.53	5.57 ± 0.70	8.06 ± 0.48	0.77 ± 0.05	6.89 ± 0.12	8.9
**10**	17.28 ± 0.90	>100	>20	>20	N.D.^c^	
**11**	12.39 ± 1.56	38.94 ± 4.89	>20	2.26 ± 0.07	>20	>8.8
**12**	>100	25.16 ± 2.58	>20	3.83 ± 0.28	>20	>5.2
**13**	>100	15.07 ± 1.48	>20	3.50 ± 0.26	>20	>5.7
**14**	16.07 ± 0.27	6.92 ± 1.10	6.40 ± 0.12	0.58 ± 0.03	5.69 ± 0.16	9.8
**15**	38.31 ± 2.65	10.62 ± 0.85	17.33 ± 0.68	2.59 ± 0.25	>20	>7.7
**16**	>50	10.65 ± 1.26	>20	1.88 ± 0.08	>20	>10.6
**17**	15.31 ± 0.74	14.12 ± 0.79	>20	4.21 ± 0.43	>20	>4.8
**18**	36.78 ± 1.93	6.96 ± 0.47	>20	0.57 ± 0.01	7.27 ± 0.08	12.8
**19**	>100	>100	>20	>20	N.D.^c^	
**20**	>50	>20	>20	4.33 ± 0.34	>20	>4.6
**21**	11.98 ± 1.47	6.62 ± 1.79	12.57 ± 0.68	0.57 ± 0.02	6.03 ± 0.07	10.6
**22**	8.95 ± 0.23	4.42 ± 0.98	3.22 ± 0.37	0.66 ± 0.08	5.42 ± 0.05	8.2
**23**	>100	>100	>20	>20	N.D.^c^	
**24**	>100	>100	>20	>20	N.D.^c^	
**25**	>100	>100	>20	>20	N.D.^c^	
**26**	>100	>100	>20	>20	N.D.^c^	
**27**	>20	>100	>20	>20	>20	
**28**	>100	>100	>20	>20	N.D.^c^	
**29**	7.59 ± 0.16	13.08 ± 1.59	5.13 ± 0.29	3.88 ± 0.23	5.49 ± 0.09	1.4
**30**	17.55 ± 0.06	16.03 ± 1.35	11.22 ± 1.01	5.89 ± 0.32	10.97 ± 0.33	1.9
**31**	12.62 ± 1.51	11.32 ± 1.09	7.39 ± 0.22	3.07 ± 0.29	6.87 ± 0.21	2.2
bortezomib	0.69 ± 0.2	0.46 ± 0.01	7.86 ± 1.23	5.24 ± 0.79	0.09 ± 0.01	0.017
CCF642	12.97 ± 0.29	6.42 ± 0.29	>20	8.77 ± 0.29	5.57 ± 0.25	0.64

a Cells were incubated with test compounds for 24 h, then the cytotoxicity was determined with the resazurin assay. All results are presented as mean ± SEM (*n* = 3).

b Selectivity index.

c Not determined.

### JUG Derivatives Inhibit Tumor Cell-Induced Platelet Aggregation

**Figure 4:**
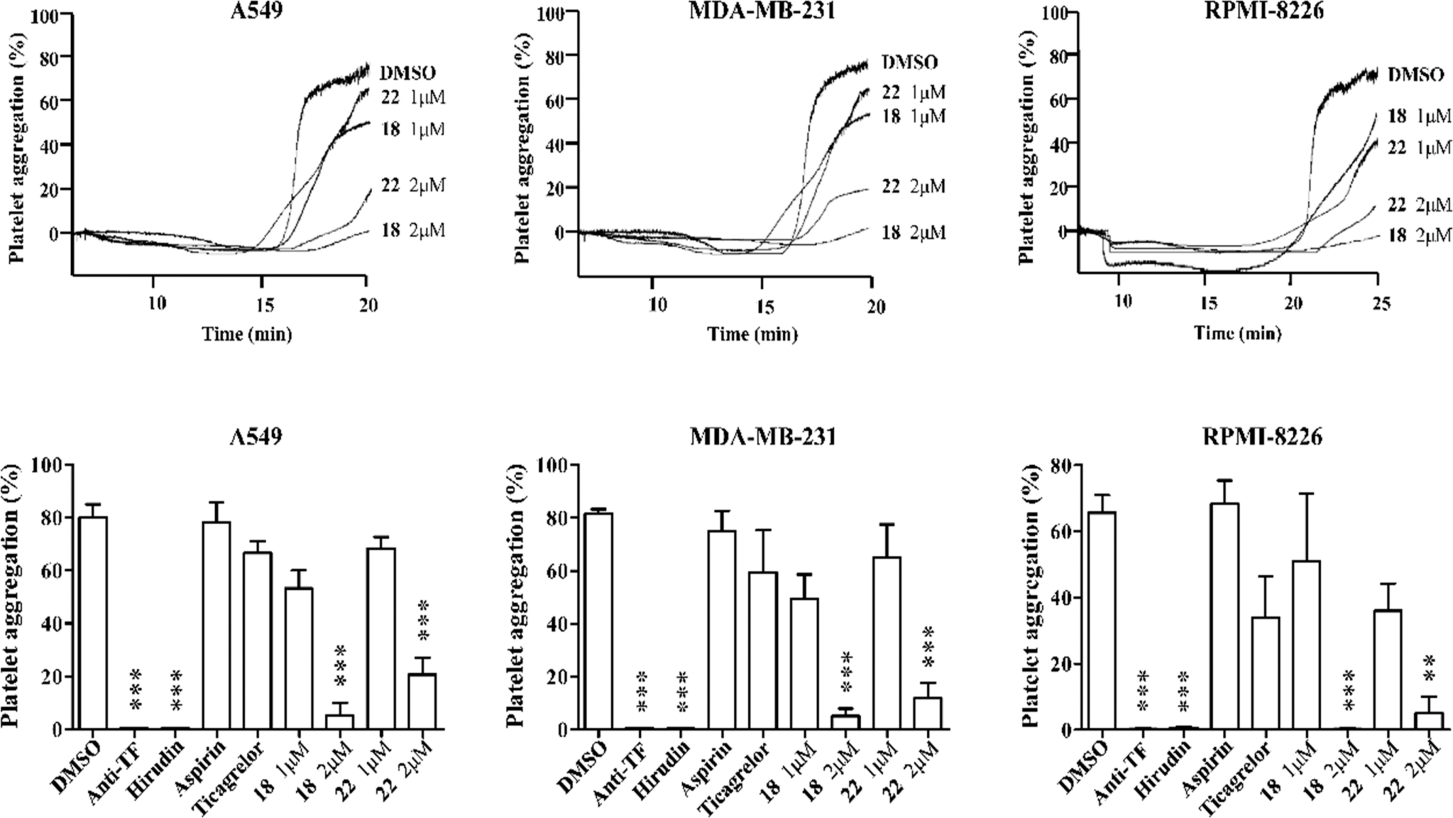
Inhibitory effects of JUG derivatives on tumor cell-induced platelet aggregation. Washed human platelets were coincubated with A549, MDA-MB-231, or RPMI8226 cancer cells in the presence of dimethyl sulfoxide (DMSO) (control), **18** or **19** at the concentrations of 1 or 2 μM. Platelet aggregation was measured using by turbidimetric aggregometry. The anti-TF antibody (20 μg/mL) and thrombin inhibitor hirudin (0.01 μM) as well as antiplatelet agents aspirin (100 μM) and ticagrelor (1 μM) were used for comparison with the JUG derivatives. Representative traces (upper panels) and quantitative results (lower panels) of platelet aggregation were shown (*n* = 3).

Given the potent antiplatelet and anticancer activity of **18** and **22**, these two compounds were evaluated for their ability to inhibit tumor cell-induced platelet aggregation (TCIPA). As shown in Figure 4, when platelets were coincubated with A549, MDA-MB-231, or RPMI8226 cells in the presence of plasma, platelet aggregation occurred within 20 min. The TCIPA was dependent on cancer expression of tissue factor (TF), which elicits activation of plasma coagulation factors and generation of thrombin, the latter subsequently stimulates platelet aggregation. As a result, a blocking antibody to TF and the thrombin inhibitor hirudin were able to prevent the TCIPA. In contrast, the clinical antiplatelet drugs aspirin and ticagrelor had no or little effect on the TCIPA, probably due to the resistance of thrombin-induced platelet aggregation to cyclooxygenase and ADP P2Y_12_ inhibition by aspirin and ticagrelor, respectively.^52^ Importantly, both **18** and **22** significantly prevented TCIPA caused by the three cancer cell lines at 2 μM.

### JUG Derivatives Inhibit Platelet-Enhanced Cancer Cell Growth

**Figure 5:**
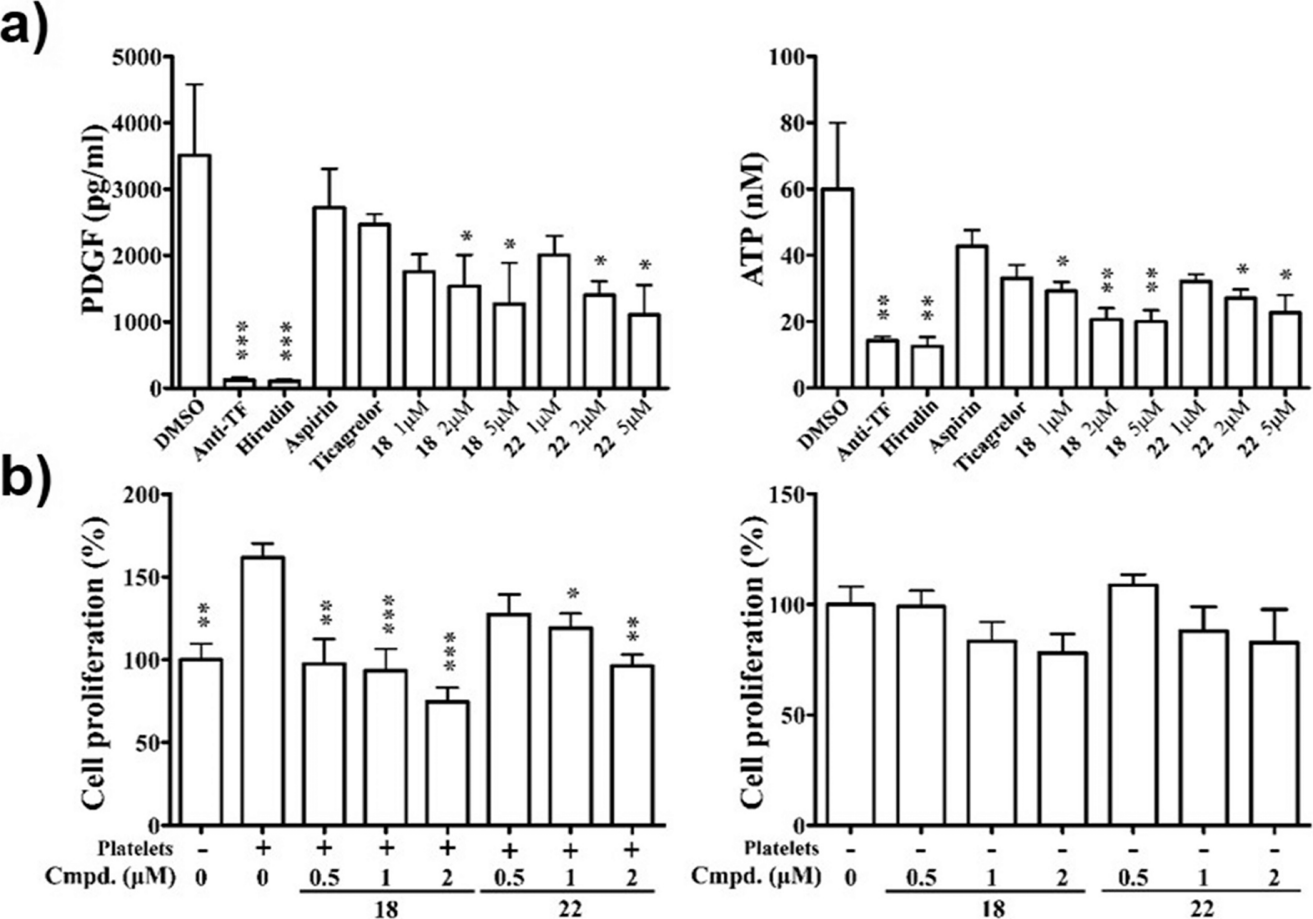
Inhibitory effects of JUG derivatives **18** and **22** on tumor cell-induced platelet secretion and platelet-enhanced tumor cell proliferation. (a) Washed human platelets were coincubated with A549 cancer cells under the same conditions as described in Figure 4. Secretion of PDGF and ATP were determined with the enzyme-linked immunosorbent assay (ELISA) and bioluminescent assay, respectively. (b) A549 cancer cells were coincubated with washed human platelets at 37 °C and 5% CO_2_ in a cell culture incubator for 48 h. After washing with PBS, the numbers of viable cancer cells were determined by trypan blue exclusion. Data are mean ± SEM of at least three independent experiments. In (a), *P < 0.05; **P < 0.01; ***P < 0.001 as compared with vehicle control (DMSO). In (b), *P < 0.05; **P < 0.01; ***P < 0.001 as compared with A549 cells plus platelets.

We next investigated if the JUG derivatives also inhibited tumor cell-induced platelet secretion, and thus prevented platelet-enhanced tumor cell growth. Figure 5a shows that A549 cancer cells coincubated with platelets caused an increase in extracellular levels of PDGF and ATP that is mainly due to TF/thrombin-mediated platelet activation and secretion. This event was reduced by **18** and **22**, but not aspirin or ticagrelor. In order to examine platelet-enhanced cancer cell growth, A549 cancer cells were coincubated with platelets for 48 h. As shown in Figure 5b, cancer cell growth was increased by 61.6% as compared with cancer cells alone. Compound **18** and, to a lesser extent, **22** blunted platelet-enhanced cancer cell growth in a concentration range from 0.5 to 2 μM. At the same concentrations, the two compounds did not significantly reduce cancer cell growth in the absence of platelets.

### JUG Derivatives Showed Covalent Inhibition Activity against PDI

**Figure 6:**
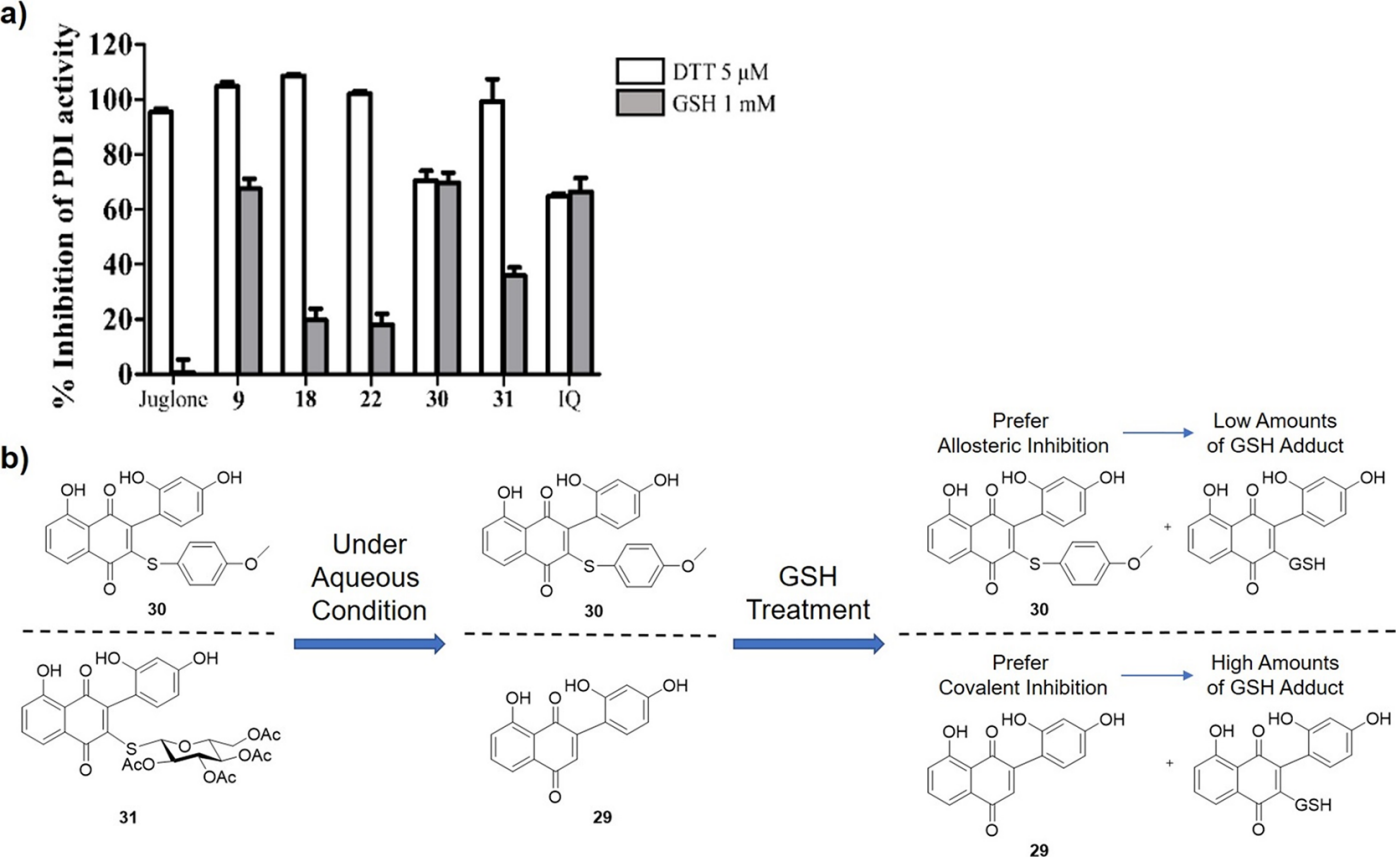
Effects of GSH on the PDI-inhibitory activities of JUG derivatives. (a) Human rPDI was incubated with JUG, JUG derivatives or isoquercetin (IQ; all at 10 μM) in the presence of either DTT (5 μM) or GSH (1 mM), and the PDI activity was determined using the Di-E-GSSG assay. Data are mean ± SEM of three independent experiments. (b) Metabolites of compounds **30** and **31** under aqueous conditions and after GSH treatment using UPLC-MS analysis.

GSH is the major cellular nonprotein thiol, which exists within cells at millimolar concentrations (1–8 mM) while is present extracellularly at only a few micromolar.^53^ The high concentrations of intracellular GSH thus may compete with the binding of PDI to covalent PDI inhibitors.^54^ We investigated if the PDI-inhibitory effects of the JUG and JUG derivatives **9**, **18**, **22**, **30**, and **31** can be affected by GSH. In this assay, the noncovalent PDI inhibitor isoquercetin was used as a negative control. As shown in Figure 6a, JUG completely lost PDI-inhibitory activity in the presence of 1 mM GSH, while the JUG derivatives had different susceptibility to GSH. The rank order for susceptibility to GSH (high to low) was JUG > **22** > **18** > **31** > **9** > **30** = isoquercetin. These results showed that JUG as well as monosubstituted JUG derivatives **18** and **22** were more susceptible to GSH treatment than compound **9**, indicating that the chemical properties of the substitutions can differently affect the thiol reactivity of juglone derivatives to PDI and GSH. On the other hand, the disubstituted JUG derivatives **30** and **31** were structure analogs of isoquercetin (**5**) and had no Michael acceptor sites, which were considered to be noncovalent inhibitors and the inhibition activity should not be influenced by GSH treatment. Surprisingly, in contrast to **30**, inhibition activity of compound **31** was largely reduced by GSH. To investigate this effect, UPLC-MS was used to analyze the metabolites of compounds **30** and **31** during enzyme assay (Figures 6b and S1–S4). The results showed that the glucose moiety of compound **31** was easily hydrolyzed in aqueous condition and the major active ingredient during assay was compound **29**, which contained a Michael acceptor (Figure S3). On the other hand, the UPLC-MS analysis of **30** showed that the compound remained intact in aqueous conditions (Figure S1). After GSH treatment, compound **31** showed a significant amount of GSH addition product, whereas only a small amount of the compound **30**-GSH adduct was found (Figure S5) These results suggest that the hydrolytic product of **31**, i.e., **29**, and the monosubstituted JUG derivatives act like covalent inhibitors, while **30** acts like the noncovalent inhibitor isoquercetin (Figures S2 and S4).

### Covalent Inhibition Binding Model of JUG Derivatives Generated by Computer Simulation

**Figure 7:**
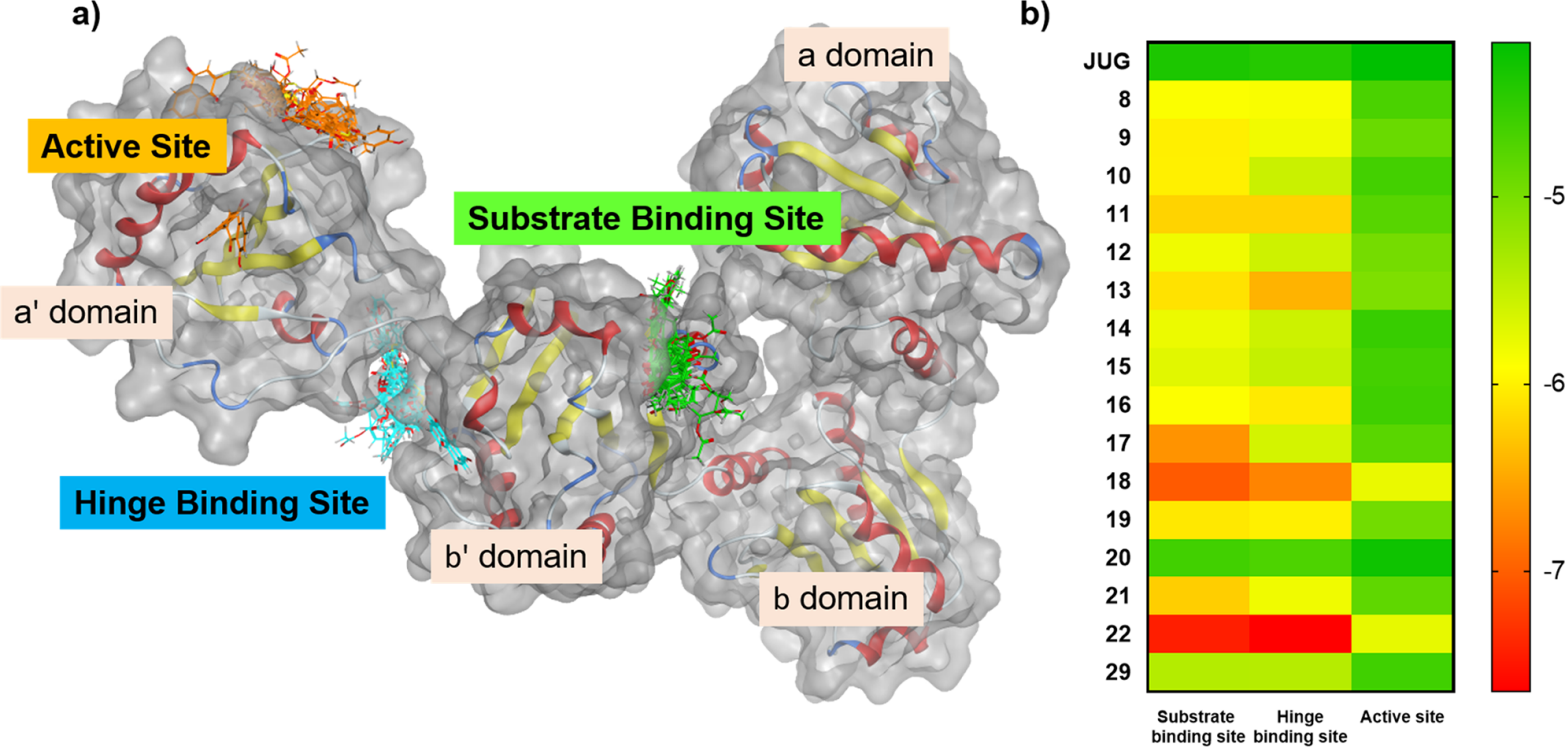
Interaction of JUG and monosubstituted JUG derivatives with PDI protein. (a) Molecular docking of JUG and **16** JUG derivatives into active site, substrate binding sites, and hinge binding site of PDI (PDB 6i7s). (b) Heat map plot for the binding energy of JUG and JUG derivatives in three binding pockets.

**Figure 8:**
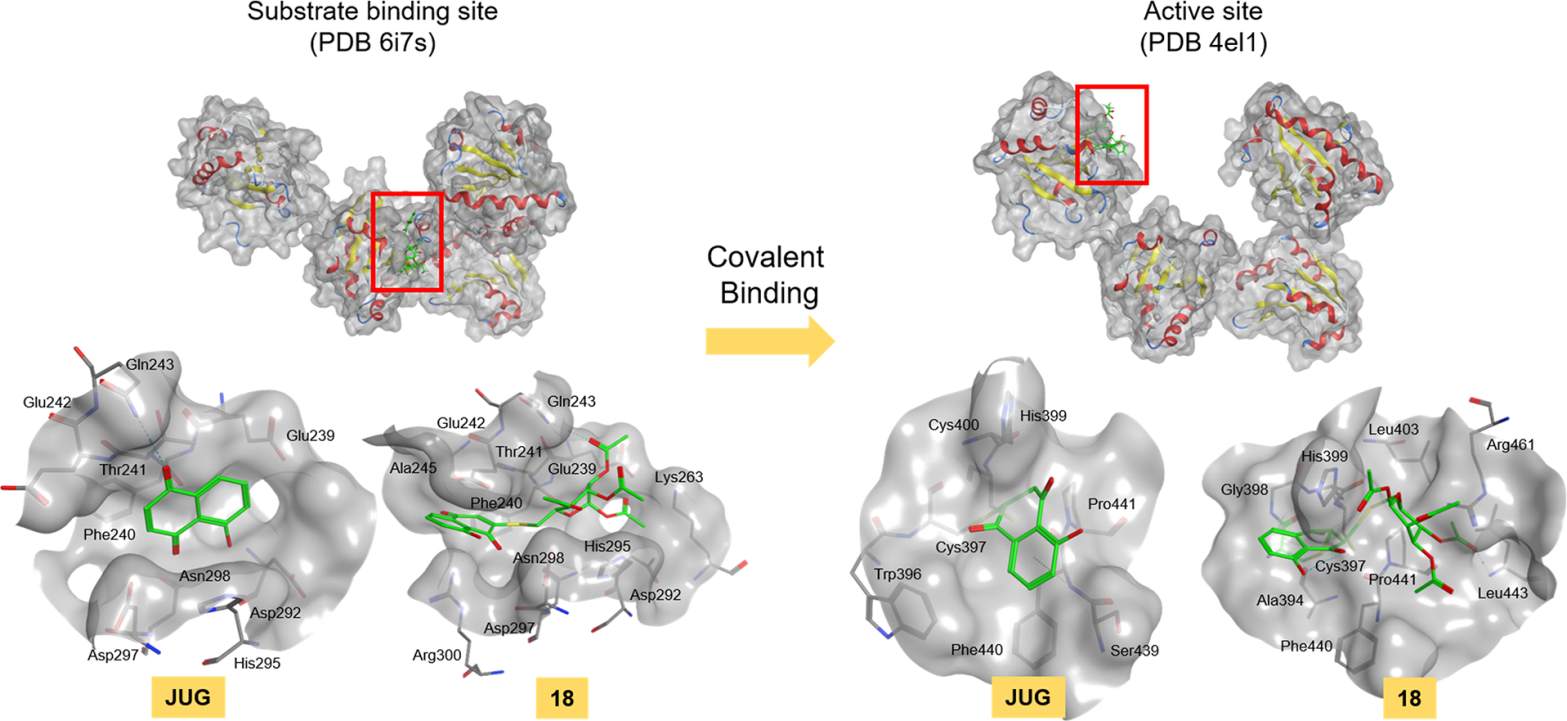
Covalent inhibition model of JUG and **18** binding to the PDI protein (PDB 6i7s and 4el1). Compounds were first bound to the substrate binding site in the b′ domain and the Michael acceptor of the compounds were attacked by the Cys397 in the a′ domain to form covalent bond.

Computer simulation was performed in molecular operating environment (MOE) to investigate the inhibition mechanism of JUG and JUG derivatives against PDI protein. The high flexibility of PDI protein posed a challenge to obtain an ideal protein model for computer simulation.^55^ One recent study utilizing the multiparameter confocal single-molecule fluorescence resonance energy transfer (FRET) method revealed that the covalent inhibitors were prone to inhibit reduced-PDI protein and induce a conformational change, which made the protein acquire the conformation similar to oxidation-PDI.^56^ Furthermore, the FRET experiment showed that the reduced-PDI in solution was preferred to stay in the open conformation rather than the closed conformation reported in the previous crystal structure.^57^ Therefore, the reduced-PDI in open conformation (PDB: 6i7s), which was cocrystallized with microsomal triglyceride transfer protein, was used in our computer simulation experiment.^58^ The superimposition of the PDI proteins from 6i7s, 4ekz, and 4el1 revealed that the reduced-PDI in 6i7s exhibited similar open conformation to the oxidized-PDI in 4el1 compared to the closed conformation in 4ekz, indicating that the PDI in 6i7s could reflect the reduced-PDI conformation in solution (Figure S6a). To investigate the potential covalent inhibition mechanism of monosubstituted JUG derivatives, the active site Cys397 and two pockets related to rutin, a PDI inhibiting flavonoid sharing a similar structure to isoquercetin and JUG, were used in derivatives library docking (Figure 7a). First, the catalytic binding site around Cys397 in PDI a′ domain is related to the activity of PDI in disulfide bond formation and cleavage.^17^ Second, the substrate binding pocket in the PDI b′ domain was revealed by point mutation, which is related to the activity of PDI substrate identification and binding.^59,60^ Third, the pocket in the hinge region of PDI between the a′ and b′ domain was revealed by molecular dynamics, and the binding of molecules might restrict the conformation of PDI.^61^ The results showed that monosubstituted JUG derivatives exhibited better binding affinity to the substrate binding pocket of PDI than the hinge binding pocket and the active site, indicating that the inhibition mechanism of monosubstituted JUG derivatives might start with the binding to the substrate binding site and induce conformational change to make the compounds attach to Cys397 covalently (Figure 7b). The covalent binding model of JUG and **18** are presented in Figure 8. First, the compounds were bound to the substrate binding site, and the docking results showed that compound **18** occupied more space to the substrate binding site and both of them showed hydrogen bond interaction with Gln243. Since the conformation of reduced-PDI was reported to be turned into the conformation similar to oxidized-PDI during inhibition by covalent inhibitors^56^ and the binding affinities of monosubstituted JUG derivatives to the active site were better in oxidized-PDI than reduced-PDI, the oxidized-PDI from 4el1 was used in covalent docking to Cys397 (Figure S6b). In the covalent docking model, compound **18** conquered a larger region in the catalytic pocket than JUG. The hydrogen bond derived from Leu443 was showed in the binding model of **18**, whereas JUG only showed one aromatic-hydrogen interaction with Phe440, which might result in the enhanced inhibition activity. Based on the docking pose of compounds, Gln243, Phe440, and Leu443 are important residues for the covalent inhibition of JUG and **18** against PDI (Figure 8).

### Elucidation of Inhibition Selectivity against PDI Protein in JUG Derivatives

In the present work, we focused on PDI, the prototype of the PDI family, as the target of JUG derivatives. In addition to PDI, ERp57 (PDIA3) and ERp72 (PDIA4) are two other major members of the PDI family in both platelets and A549 cells.^62,63^ Therefore, the selectivity of compounds **18** and **22** against PDI over ERp57 and ERp72 were examined. As shown in Table 4, **18** and **22** inhibited ERp57 and ERp72 at concentrations about 4 to 7-fold higher than that required to inhibit PDI. In contrast, the IC_50_ values of juglone for ERp57 and ERp72 were 2.2- and 2.9-fold higher than PDI IC_50_.

**Table 4:** Inhibitory Activity of JUG Derivatives against PDI, ERp57, and ERp72

IC_50_(μM)^a^			
	PDI	ERp57	ERp72
**7** (JUG)	1.10 ± 0.17	2.40 ± 0.23	3.16 ± 0.57
**18**	1.87 ± 0.56	6.98 ± 1.78	13.66 ± 0.66
**22**	0.63 ± 0.12	3.24 ± 1.10	4.01 ± 0.39

a Reductase assay was determined using human recombinant PDIs, Di-E-GSSG, and fluorometry. Data are mean ± SEM, *n* = 3.

Besides PDIs, the peptidyl-prolyl isomerase, Pin1, is known as a potential target of juglone that can be covalently thiol modified by juglone.^64,65^ In the present study, we did not investigate if Pin1 is susceptible to the juglone derivatives that have Michael acceptor(s). However, it seems to be unlikely that Pin1 inhibition plays a major role in their bioactivities reported here. The function of Pin1 in platelets has not been described previously, and our own unpublished data does not show any effect of sulfopin, a highly selective inhibitor of Pin1,^66^ on platelet aggregation induced by either U46619 or collagen at concentrations up to 40 μM. Moreover, sulfopin also had no significant impacts on the cell viability of RPMI8226, A549, and MDA-MB-231 cancer cells (unpublished data); this result is consistent with previous studies in which neither genetic nor pharmacological inhibition of Pin1 was sufficient to affect cell viability across a wide range of cancer cell lines, suggesting Pin1 is not essential for cell survival although it may drive tumor progression in vivo.^66,67^ Another potential mechanism involved in the cytotoxicity of the JUG derivatives with Michael acceptors, e.g., **18** and **22**, is the induction of oxidative stress. It is known that juglone can cause depletion of cellular GSH by the nucleophilic addition to GSH and, to a lesser extent, by oxidation of GSH through the redox cycling and reactive oxygen species (ROS) formation.^31^

Among the cell lines tested in the present study, multiple myeloma RPMI8226 cells show the highest sensitivity to the juglone derivatives. Multiple myeloma is a malignancy of plasma cells and characterized by extensive production of abnormal immunoglobulins and thus prone to ER stress.^68^ Moreover, the augmented protein synthesis is accompanied by increased ROS formation in multiple myeloma.^69^ Therefore, multiple myeloma cells are highly dependent on the unfolded protein response pathway and the antioxidant pathway to deal with both ER stress and oxidative stress.^26^ Because PDI and GSH play important roles in unfolded protein response and the antioxidant pathway, respectively; the selective cytotoxicity of the JUG derivatives **18** and **22** toward RPMI8226 cells may be due to both PDI inhibition and GSH depletion, leading to further exaggerated ER stress and oxidative stress. In contrast to multiple myeloma cells, normal cells have lower ROS levels and protein-synthesis rates;^70^ this may explain the higher resistance of endothelial EA.hy926 cells to the JUG derivatives. Nevertheless, the potential toxicity of the JUG derivatives needs further in vitro and in vivo studies.

## Conclusions

In this study, **24** JUG derivatives were synthesized through nucleophilic addition or substitution on the Michael acceptor of JUG. The chemical modifications resulted in enhanced PDI-inhibitory activity and antiplatelet effect compared to JUG. The molecular docking showed the amino acid residues Gln243, Phe440, and Leu443 are important for the compound–protein interaction. Particularly, the glycosylated JUG derivatives **18** and **22** showed selective anticancer activity (S.I. = 12.8 and 10.6, respectively) against multiple myeloma RPMI 8226 cells that is superior than the JUG (S.I. = 1.4), the proteasome inhibitor bortezomib (S.I. = 0.017) and PDI inhibitor CCF642 (S.I. = 0.64). In addition, **18** and **22** is capable of preventing cancer cell–platelet interactions involved in cancer progression or metastasis. Together, these results suggest the synthesized JUG derivatives could serve as potential leads for the development of novel anticancer and antithrombotic agents targeting platelet–cancer interaction through the covalent inhibition of PDI.

## Experimental Section

### General Chemicals and Instrumentation

Reagents and solvents for synthesis were of reagent grade and used without further purification. High-performance liquid chromatography (HPLC) analysis was performed on a HITACHI D-2000 Elite system equipped with a BDS HYPERSIL C18 250 × 4.6 column. The column was eluted with the mobile phase at flow rate of 1.0 mL/min. The purities of all final products were confirmed by HPLC to be >95% prior to biological evaluation. Thin-layer chromatography (0.25 mm, E. Merck silica gel 60 F_254_) was used to monitor reaction progress; plates were visualized by UV (state wavelength), or by staining with ninhydrin and heating. Acquisition of ^1^H and ^13^C nuclear magnetic resonance (NMR) spectra was performed on Bruker-AV-400 (400 MHz) or Bruker-AVIII-600 (600 MHz). Chemical shifts (δ) are given in ppm and referenced to residual solvent peaks ^1^H: 7.26 ppm, ^13^C: 77.0 ppm for CDCl_3_; ^1^H: 5.32 ppm, ^13^C: 54.2 ppm for CD_2_Cl_2_; ^1^H: 3.31 ppm, ^13^C: 49.0 ppm for CD_3_OD; ^1^H: 2.50 ppm, ^13^C: 39.5 ppm for (CD_3_)_2_SO; ^1^H: 2.05 ppm, ^13^C: 29.8 ppm for (CD_3_)_2_CO. Splitting patterns are reported as s (singlet), brs (broad singlet), d (doublet), dd (double doublet), t (triplet), q (quartet), and m (multiplet). Coupling constants (*J*) are given in Hertz (Hz). Mass spectra were obtained by Bruker bioTOF III. Melting point was measured on a FARGO melting point apparatus MP-1D.

### Synthesis of JUG Derivatives **8**–**31**^71^

#### 2-((4-Methoxyphenyl)thio)-8-hydroxynaphthalene-1,4-dione (**8**)

To a stirred solution of **7** (100 mg, 0.57 mmol) in ethanol (4 mL) under the N_2_ atmosphere at 0 °C was added 4-methoxybenzenethiol (68.4 μL, 0.57 mmol) predissolved in 4 mL of ethanol. After stirring for 2 h, the reaction mixture was concentrated under reduced pressure and purified by flash column chromatography (silica gel; toluene/hexane = 2/1) to give **8** (56 mg, 31%) as an orange solid; *R*_f_ = 0.5 (toluene/hexane = 5/1); mp 175 °C; ^1^H NMR (600 MHz, CD_2_Cl_2_) δ 11.67 (s, 1H, OH), 7.62 (t, 1H, *J* = 8.1 Hz, H-7), 7.51 (dd, 1H, *J =* 1.0 Hz, 7.6 Hz, H-8), 7.46 (m, 2H), 7.24 (dd, 1H, *J* = 1.0 Hz, 7.6 Hz, H-6), 7.04 (m, 2H), 6.03 (s, 1H, H-2), 3.86 (s, 3H, OCH_3_) ppm; ^13^C NMR (150 MHz, CD_2_Cl_2_) δ 187.5 (C-4), 181.0 (C-1), 161.8 (C-5), 161.7 (C-4a), 156.9 (C-3), 137.3 (2C), 137.2, 132.4 (C-8a), 129.0 (C-2), 123.6 (C-6), 119.1 (C-8), 117.1 (C-9), 116.1 (2C), 114.8, 55.6 ppm. HRMS (ESI-TOF MS) C_17_H_13_O_4_S^+^ [M + H]^+^ calc. 313.0529, found 313.0517; HPLC purity 95.9% (*t*_R_ 20.5 min, Hypersil BDS C18, 250 × 4.6 mm, 5 μm, 1 mL/min, H_2_O/ACN = 90/10, 0–2 min, H_2_O/ACN = 10/90, 2–20 min, H_2_O/ACN = 10/90, 20–30 min).

#### 2-((3-Methoxyphenyl)thio)-8-hydroxynaphthalene-1,4-dione (**9**)

To a stirred solution of **7** (100 mg, 0.57 mmol) in ethanol (4 mL) under the N_2_ atmosphere at 0 °C was added 3-methoxybenzenethiol (68.4 μL, 0.57 mmol) predissolved in ethanol (4 mL). After stirring for 3 h, the reaction mixture was concentrated under reduced pressure and purified by column chromatography (silica gel; toluene/hexane = 1/1) to give **9** (60 mg, 34%) as an orange solid: *R*_f_ = 0.33 (DCM/hexane = 1/1); mp 152 °C; ^1^H NMR (400 MHz, CDCl_3_) δ 11.70 (s, 1H), 7.61 (t, 1H, *J =* 7.7 Hz), 7.55 (d, 1H, *J =* 7.7 Hz), 7.41 (t, 1H, *J =* 7.8 Hz), 7.23 (d, 1H, *J =* 7.7 Hz), 7.12 (d, 1H, *J =* 7.8 Hz), 7.06–7.03 (m, 2H), 6.12 (s, 1H), 3.83 (s, 3H) ppm; ^13^C NMR (100 MHz, CDCl_3_) δ 187.1, 181.3, 161.8, 160.8, 156.3, 137.1, 132.2, 131.2, 129.0, 127.8, 127.7, 123.8, 120.7, 119.3, 116.6, 114.6, 55.5 ppm; HRMS (ESI-TOF MS) C_17_H_13_O_4_S^+^ [M + H]^+^ calc. 313.0529, found 313.0515; HPLC purity 96.6% (*t*_R_ 20.5 min, Hypersil BDS C18, 250 × 4.6 mm, 5 μm, 1 mL/min, H_2_O/ACN = 90/10, 0–2 min, H_2_O/ACN = 10/90, 2–20 min, H_2_O/ACN = 10/90, 20–30 min).

#### 2-((2-Methoxyphenyl)thio)-8-hydroxynaphthalene-1,4-dione (**10**)

To a stirred solution of **7** (100 mg, 0.57 mmol) in ethanol (4 mL) under the N_2_ atmosphere at 0 °C was added 2-methoxybenzenethiol (68.4 μL, 0.57 mmol) predissolved in ethanol (4 mL) and warmed to room temperature gradually. After stirring overnight, the reaction mixture was concentrated under reduced pressure and purified by column chromatography (silica gel; toluene/hexane = 1/2) to give **10** (112 mg, 63%) as an orange solid: *R*_f_ = 0.22 (DCM/hexane = 1/1); mp 196 °C; ^1^H NMR (600 MHz, CDCl_3_) δ 11.75 (s, 1H), 7.61 (t, 1H, *J =* 7.4 Hz), 7.55 (d, 1H, *J =* 7.4 Hz), 7.54–7.50 (m, 2H), 7.22 (d, 1H, *J =* 7.4 Hz), 7.07–7.03 (m, 2H), 6.00 (s, 1H), 3.85 (s, 3H) ppm; ^13^C NMR (150 MHz, CDCl_3_) δ 187.5, 181.4, 161.7, 159.8, 154.2, 137.4, 136.9, 133.0, 132.3, 128.6, 123.7, 122.0, 119.2, 114.7, 114.2, 112.0, 56.0 ppm; HRMS (ESI-TOF MS) C_17_H_13_O_4_S^+^ [M + H]^+^ calc. 313.0529, found 313.0517; HPLC purity 96.3% (*t*_R_ 19.7 min, Hypersil BDS C18, 250 × 4.6 mm, 5 μm, 1 mL/min, H_2_O/ACN = 90/10, 0–2 min, H_2_O/ACN = 10/90, 2–20 min, H_2_O/ACN = 10/90, 20–30 min).

#### 2-((3,4-Dimethoxyphenyl)thio)-8-hydroxynaphthalene-1,4-dione (**11**)

To a stirred solution of **7** (100 mg, 0.57 mmol) in ethanol (4 mL) under the N_2_ atmosphere at −20 °C was added 3,4-dimethoxybenzenethiol (80 μL, 0.57 mmol) predissolved in ethanol (4 mL). After stirring for 16 h, the reaction mixture was concentrated under reduced pressure and purified by column chromatography (silica gel; DCM/hexane = 1/1) to give **11** (25 mg, 13%) as an orange solid; *R*_f_ = 0.20 (DCM/hexane = 1/1); mp 166 °C; ^1^H NMR (400 MHz, CDCl_3_) δ 11.71 (s, 1H), 7.61 (t, 1H, *J =* 7.6 Hz), 7.54 (dd, 1H, *J =* 0.9 Hz, 7.6 Hz), 7.22 (dd, 1H, *J =* 0.9 Hz, 8.3 Hz), 7.12 (dd, 1H, 0.9 Hz, 7.6 Hz), 6.97–6.95 (m, 2H), 6.09 (s, 1H), 3.94 (s, 3H), 3.88 (s, 3H) ppm; ^13^C NMR (100 MHz, CDCl_3_) δ 187.3, 181.4, 161.8, 157.1, 151.2, 150.2, 137.1, 132.2, 129.1, 129.0, 128.4, 123.8, 119.4, 117.6, 117.1, 114.6, 112.5, 56.1, 56.0 ppm; HRMS (ESI-TOF MS) C_18_H_15_O_5_S^+^ [M + H]^+^ calc. 343.0635, found 343.0620; HPLC purity 96.4% (*t*_R_ 18.7 min, Hypersil BDS C18, 250 × 4.6 mm, 5 μm, 1 mL/min, H_2_O/ACN = 90/10, 0–2 min, H_2_O/ACN = 10/90, 2–20 min, H_2_O/ACN = 10/90, 20–30 min).

#### 2-(*p*-Tolylthio)-8-hydroxynaphthalene-1,4-dione (**12**)

To a stirred solution of **7** (50 mg, 0.29 mmol) in ethanol (2 mL) under the N_2_ atmosphere at 0 °C was added 4-methylbenzenethiol (37 mg, 0.29 mmol) predissolved in ethanol (1 mL). After stirring for 4 h, the reaction mixture was concentrated under reduced pressure and purified by column chromatography (silica gel; toluene/hexane = 1/1) to give **12** (14 mg, 16%) as an orange solid; *R*_f_ = 0.3 (DCM/hexane = 1/2); mp 176 °C; ^1^H NMR (600 MHz, CDCl_3_) δ 11.71 (s, 1H), 7.61 (t, 1H, *J =* 7.6 Hz), 7.54 (dd, 1H, *J =* 0.9 Hz, 7.3 Hz), 7.40 (d, 2H, *J =* 8.0 Hz), 7.30 (d, 2H, *J =* 8.0 Hz), 7.22 (dd, 1H, *J =* 0.9 Hz, 8.0 Hz), 6.08 (s, 1H), 2.43 (s, 3H) ppm; ^13^C NMR (150 MHz, CDCl_3_) δ 187.2, 181.2, 161.9, 156.7, 141.1, 137.0, 135.6, 132.3, 131.2, 128.9, 123.7, 123.4, 119.3, 114.7, 21.3 ppm; HRMS (ESI-TOF MS) C_17_H_13_O_3_S^+^ [M + H]^+^ calc. 297.0580, found 297.0579; HPLC purity 95.1% (*t*_R_ 22.2 min, Hypersil BDS C18, 250 × 4.6 mm, 5 μm, 1 mL/min, H_2_O/ACN = 90/10, 0–2 min, H_2_O/ACN = 10/90, 2–20 min, H_2_O/ACN = 10/90, 20–30 min).

#### 2-((4-(*tert*-Butyl)phenyl)thio)-8-hydroxynaphthalene-1,4-dione (**13**)

To a stirred solution of **7** (100 mg, 0.57 mmol) in ethanol (4 mL) under the N_2_ atmosphere at 0 °C was added 4-*t*-butylbenzenethiol (94 μL, 0.57 mmol) predissolved in ethanol (4 mL). After stirring for 3 h, the reaction mixture was concentrated under reduced pressure and purified by column chromatography (silica gel; toluene/hexane = 2/3) to give **13** (31 mg, 16%) as an orange solid; *R*_f_ = 0.53 (DCM/hexane = 1/1); mp 179 °C; ^1^H NMR (400 MHz, CD_3_OD) δ 11.72 (s, 1H), 7.63–7.45 (m, 6H), 7.23 (dt, 1H, *J* = 1.2 Hz, 8.3 Hz), 6.14 (s, 1H), 1.36 (s, 9H) ppm; ^13^C NMR (100 MHz, CD_3_OD) δ 187.3, 181.4, 161.9, 156.7, 154.3, 137.1, 135.4, 132.3, 128.9, 127.6, 123.8, 123.2, 119.4, 114.7, 35.0, 31.2 ppm; HRMS (ESI-TOF MS) C_20_H_19_O_3_S^+^ [M + H]^+^ calc. 339.1049, found 339.1032; HPLC purity 95.1% (*t*_R_ 25.6 min, Hypersil BDS C18, 250 × 4.6 mm, 5 μm, 1 mL/min, H_2_O/ACN = 90/10, 0–2 min, H_2_O/ACN = 10/90, 2–20 min, H_2_O/ACN = 10/90, 20–30 min).

#### 2-((4-Hydroxyphenyl)thio)-8-hydroxynaphthalene-1,4-dione (**14**)

To a stirred solution of **7** (50 mg, 0.29 mmol) in ethanol (1 mL) under the N_2_ atmosphere at 0 °C was added 4-mercaptophenol (35 mg, 0.29 mmol) predissolved in ethanol (1 mL). After stirring overnight, the reaction mixture was concentrated under reduced pressure and purified by column chromatography (silica gel; toluene/hexane = 1/1) to give **14** (43 mg, 50%) as an orange solid; *R*_f_ = 0.61 (EtOAc/hexane = 1/1); mp 240 °C; ^1^H NMR (400 MHz, (CD_3_)_2_CO) δ 11.66 (s, 1H),9.23 (brs, 1H), 7.77 (t, 1H, *J =* 7.6 Hz), 7.52–7.50 (m, 1H), 7.47–7.45 (m, 2H), 7.35 (s, 1H), 7.31–7.28 (m, 1H), 7.08–7.06 (m, 2H), 5.97 (s, 1H) ppm; ^13^C NMR (100 MHz, (CD_3_)_2_CO) δ 187.4, 180.5, 161.6, 161.4, 156.9, 137.4, 132.4, 128.5, 128.3, 123.4, 123.3, 118.7, 117.6, 117.5, 115.6, 114.8 ppm; HRMS (ESI-TOF MS) C_17_H_11_O_3_S^–^ [M – H]^−^ calc. 297.0227, found 297.0218; HPLC purity 96.1% (*t*_R_ 16.6 min, Hypersil BDS C18, 250 × 4.6 mm, 5 μm, 1 mL/min, H_2_O/ACN = 90/10, 0–2 min, H_2_O/ACN = 10/90, 2–20 min, H_2_O/ACN = 10/90, 20–30 min).

#### 2-((4-Fluorophenyl)thio)-8-hydroxynaphthalene-1,4-dione (**15**)

To a stirred solution of **7** (100 mg, 0.57 mmol) in ethanol (4 mL) under the N_2_ atmosphere at −20 °C was added 4-fluorobenzenethiol (59.7 μL, 0.57 mmol) predissolved in ethanol (4 mL). After stirring for 3 h, the reaction mixture was concentrated under reduced pressure and purified by column chromatography (silica gel; toluene/hexane = 2/3) to give **15** (25 mg, 15%) as an orange solid; *R*_f_ = 0.44 (toluene/hexane = 2/1); mp 168 °C; ^1^H NMR (400 MHz, CDCl_3_) δ 11.69 (s, 1H), 7.64 (t, 1H, *J =* 7.8 Hz), 7.58–7.54 (m, 3H), 7.26–7.21 (m, 3H), 6.04 (s, 1H) ppm; ^13^C NMR (100 MHz, CDCl_3_) δ 187.0, 181.2, 164.3 (d, ^3^*J*_CF_ = 251.2 Hz, C-4′), 161.8, 156.2, 138.0 (d, ^3^*J*_CF_ = 9.8 Hz, C-2′, C-6′), 137.2, 132.1, 128.9, 123.9, 122.1 (d, ^4^*J*_CF_ = 3.7 Hz, C-1′), 119.4, 117.9 (d, ^2^*J*_CF_ = 24.2 Hz, C-3′, C-5′), 114.5 ppm; HRMS (ESI-TOF MS) C_16_H_10_FO_3_S^+^ [M + H]^+^ calc. 301.0329, found 301.0320; HPLC purity 96.4% (*t*_R_ 20.0 min, Hypersil BDS C18, 250 × 4.6 mm, 5 μm, 1 mL/min, H_2_O/ACN = 90/10, 0–2 min, H_2_O/ACN = 10/90, 2–20 min, H_2_O/ACN = 10/90, 20–30 min).

#### 2-(Pentylthio)-8-hydroxynaphthalene-1,4-dione (**16**)

To a stirred solution of **7** (50 mg, 0.29 mmol) in ethanol (2 mL) under the N_2_ atmosphere at −20 °C was added 1-pentanethiol (34.7 μL, 0.29 mmol) predissolved in ethanol (1 mL) under the N_2_ atmosphere. After stirring for 2 h, the reaction mixture was concentrated under reduced pressure and purified by column chromatography (silica gel; CHCl_3_/hexane = 1/3) to give **16** (18 mg, 22%) as an orange solid; *R*_f_ = 0.47 (CHCl_3_/hexane = 2/1); mp 144 °C; ^1^H NMR (600 MHz, CD_2_Cl_2_) δ 11.68 (s, 1H), 7.64 (t, 1H, *J* = 7.7 Hz), 7.56–7.58 (m, 1H), 7.21 (dd, 1H, *J* = 0.96 Hz, 7.7 Hz), 6.57 (s, 1H), 2.85 (t, 2H, *J =* 7.4 Hz), 1.77 (q, 2H, *J =* 7.4), 1.50–1.45 (m, 2H), 1.41–1.37 (m, 2H), 0.93 (t, 3H, *J =* 7.5 Hz) ppm; ^13^C NMR (150 MHz, CD_2_Cl_2_) δ 187.8, 181.0, 162.2, 155.2, 137.5, 132.7, 128.3, 123.8, 119.4, 115.3, 31.6, 31.1, 27.4, 22.6, 14.0 ppm; HRMS (ESI-TOF MS) C_15_H_17_O_3_S^+^ [M + H]^+^ calc. 277.0893, found 277.0887; HPLC purity 96.6% (*t*_R_ 22.5 min, Hypersil BDS C18, 250 × 4.6 mm, 5 μm, 1 mL/min, H_2_O/ACN = 90/10, 0–2 min, H_2_O/ACN = 10/90, 2–20 min, H_2_O/ACN = 10/90, 20–30 min).

#### 2-Bromo-8-hydroxynaphthalene-1,4-dione (**17**)

To a stirred solution of **7** (100 mg, 0.57 mmol) and acetic acid (10 μL) in CHCl_3_ (1.5 mL) under the N_2_ atmosphere at 0 °C was added bromine (15 μL, 0.56 mmol) predissolved in CHCl_3_ (1.5 mL) dropwise and protected from light. After stirring overnight, the reaction mixture was concentrated under reduced pressure and then recrystallized with DCM/ethanol to give **17** (84 mg, 60%) as an orange solid; *R*_f_ = 0.55 (DCM/hexane = 1/1); mp 174 °C; ^1^H NMR (400 MHz, CDCl_3_) δ 11.73 (s, 1H), 7.71–7.60 (m, 2H), 7.49 (s, 1H), 7.32–7.29 (dd, 1H, *J* = 1.6 Hz, 7.9 Hz) ppm; ^13^C NMR (100 MHz, CDCl_3_) δ 182.9, 181.6, 162.0, 141.2, 139.3, 137.2, 131.7, 124.8, 119.9, 114.0 ppm; HRMS (ESI-TOF MS) C_10_H_4_BrO_3_^–^ [M – H]^−^ calc. 250.9349, found 250.9341; HPLC purity 95.2% (*t*_R_ 15.6 min, Hypersil BDS C18, 250 × 4.6 mm, 5 μm, 1 mL/min, H_2_O/ACN = 90/10, 0–2 min, H_2_O/ACN = 10/90, 2–20 min, H_2_O/ACN = 10/90, 20–30 min).

#### 2-(1,2,3,4-Tetra-O-acetyl-6-deoxy-6-thio-α-d-glucopyranose)-8-hydroxynaphthalene-1,4-dione (**18**)

To a stirred solution of **7** (24 mg, 0.14 mmol) in ethanol (1 mL) was added a solution of 1,2,3,4-tetra-*O*-acetyl-6-deoxy-6-thio-α-d-glucopyranose (50 mg, 0.14 mmol) predissolved in ethanol (1 mL) under the N_2_ atmosphere at −20 °C. After stirring for 3 h, the reaction mixture was filtered, collected the solid, and recrystallized with ethanol to give **18** (46 mg, 61%) as a yellow solid; *R*_f_ = 0.61 (EtOAc/hexane = 1/1); mp 217 °C; ^1^H NMR (400 MHz, CDCl_3_) δ 11.65 (s, 1H), 7.66–7.59 (m, 2H), 7.24–7.22 (dd, 1H, *J* = 1.6 Hz, 8.0 Hz), 6.58 (s, 1H), 6.32 (d, 1H, *J* = 3.6 Hz), 5.46 (t, 1H, *J* = 9.8 Hz), 5.13–5.07 (m, 2H), 4.25–4.19 (m, 1H), 3.06–2.94 (m, 2H), 2.17 (s, 3H), 2.13 (s, 3H), 2.03 (s, 3H), 2.01 (s, 3H) ppm; ^13^C NMR (100 MHz, CDCl_3_) δ 186.8, 180.8, 170.2, 169.74, 169.67, 168.7, 161.9, 153.5, 137.3, 131.9, 128.2, 123.9, 119.5, 114.6 ppm; HRMS (ESI-TOF MS) C_24_H_25_O_12_S^+^ [M + H]^+^ calc. 537.1061, found 537.1061; HPLC purity 95.3% (*t*_R_ 18.4 min, Hypersil BDS C18, 250 × 4.6 mm, 5 μm, 1 mL/min, H_2_O/ACN = 90/10, 0–2 min, H_2_O/ACN = 10/90, 2–20 min, H_2_O/ACN = 10/90, 20–30 min).

#### 2-(6-Deoxy-6-thio-d-glucose)-8-hydroxynaphthalene-1,4-dione (**19**)

To a stirred solution of **18** (53.6 mg, 0.10 mmol) in methanol (10 mL) was added NaOMe (0.3 mL) at 0 °C. After stirring for 1 h, the reaction mixture was quenched by Amberlite 120 (H^+^ form), filtered and concentrated. The residue was purified by column chromatography (silica gel; methanol/acetone/EtOAc/toluene = 1/2/2/10) to give α/β anomeric mixture **19** (46 mg, 38%) as a yellow solid; *R*_f_ = 0.38 (methanol/EtOAc/benzene = 2/4/7); ^1^H NMR (600 MHz, (CD_3_)_2_SO, α/β = 3/2) δ 7.78–7.73 (m, 1H), 7.53–7.49 (m, 1H), 7.34–7.28 (m, 1H), 6.83–6.79 (m, 1H, H-3), 4.91 (d, *J* = 3.6 Hz, 0.6H, H-1′, α), 4.33 (d, *J* = 7.7 Hz, 0.4H, H-1′, β), 3.91–3.81 (m, 1H), 3.47–3.39 (m, 1H), 3.22–2.90 (m, 4H) ppm (the rest of the peaks belong to OH, including one Ar–OH and four Glu-OH in α and β form); ^13^C NMR (150 MHz, (CD_3_)_2_SO) δ: 186.2, 180.4, 154.5, 137.3, 132.1, 127.6, 123.3, 118.6, 114.8, 92.4, 73.4, 72.9, 72.4, 72.0, 69.0, 32.7, 33.4 ppm; HRMS (ESI-TOF MS) C_16_H_17_O_8_S^+^ [M + H]^+^ calc. 369.0639, found 369.0642; HPLC purity 95.2% (combined α and β form, *t*_R_ 11.95, 11.99 min, Hypersil BDS C18, 250 × 4.6 mm, 5 μm, 1 mL/min, H_2_O/ACN = 90/10, 0–2 min, H_2_O/ACN = 10/90, 2–20 min, H_2_O/ACN = 10/90, 20–30 min).

#### 2-((4-(*tert*-Butyl)benzyl)thio)-8-hydroxynaphthalene-1,4-dione (**20**)

To a stirred solution of **17** (50 mg, 0.20 mmol) and K_2_CO_3_ (29.0 mg, 0.21 mmol) in DMF (5 mL) under the N_2_ atmosphere was added 4-*tert*-butylbenzyl mercaptan (36.3 μL, 0.20 mmol) predissolved in DMF (5 mL). After stirring for 4 h, the reaction mixture was concentrated under reduced pressure and purified by column chromatography (silica gel; toluene/hexane = 1/2) to give **20** (10 mg, 15%) as an orange solid; *R*_f_ = 0.46 (toluene/hexane = 1/1); mp 136 °C; ^1^H NMR (400 MHz, CDCl_3_) δ 12.15 (s, 1H), 7.64–7.63 (m, 1H), 7.55 (t, 1H, *J =* 8.0 Hz), 7.38–7.37 (m, 2H), 7.33–7.32 (m, 2H,), 7.25–7.24 (m, 1H), 6.62 (s, 1H), 4.05 (s, 2H), 1.30 (s, 9H) ppm; ^13^C NMR (100 MHz, CDCl_3_) δ 186.7, 181.2, 161.3, 156.0, 151.0, 135.4, 131.5, 130.4, 128.4 (2C), 126.7, 125.8, 124.9 (2C), 119.6, 114.4, 35.1, 34.4, 31.1(3C) ppm; HRMS (ESI-TOF MS) C_21_H_20_O_3_S^+^ [M + H]^+^ calc. 353.1206, found 353.1189; HPLC purity 95.1% (*t*_R_ 25.6 min, Hypersil BDS C18, 250 × 4.6 mm, 5 μm, 1 mL/min, H_2_O/ACN = 90/10, 0–2 min, H_2_O/ACN = 10/90, 2–20 min, H_2_O/ACN = 10/90, 20–30 min).

#### 2-(4-Methoxyphenoxy)-8-hydroxynaphthalene-1,4-dione (**21**)

To a stirred solution of **17** (50 mg, 0.20 mmol) in DMF (5 mL) under the N_2_ atmosphere was added a solution of K_2_CO_3_ (28.4 mg, 0.21 mmol) and 4-methoxyphenol (24.4 mg, 0.20 mmol) in DMF (5 mL). After stirring for 4 h, the reaction mixture was concentrated under reduced pressure and purified by column chromatography (silica gel; DCM/hexane = 1/1) to give **21** (23 mg, 39%) as a yellow solid; *R*_f_ = 0.25 (DCM/hexane = 2/1); mp 167 °C; ^1^H NMR (600 MHz, CD_2_Cl_2_) δ 11.77 (s, 1H), 7.65 (t, 1H, *J* = 7.7 Hz), 7.55 (dd, 1H, *J =* 0.84 Hz, 7.7 Hz), 7.26 (dd, 1H, *J* = 0.8 Hz, 4.3 Hz), 7.08–7.07 (m, 2H), 6.99–6.97 (m, 2H), 5.89 (s, 1H), 3.82 (s, 3H) ppm; ^13^C NMR (150 MHz, CD_2_Cl_2_) δ 185.3, 184.2, 162.3, 161.0, 158.4, 146.2, 137.5, 132.5, 124.1, 122.2, 119.0, 115.7, 114.0, 56.1 ppm; HRMS (ESI-TOF MS) C_17_H_13_O_5_^+^ [M + H]^+^ calc. 297.0757, found 297.0747; HPLC purity 95.4% (*t*_R_ 20.3 min, Hypersil BDS C18, 250 × 4.6 mm, 5 μm, 1 mL/min, H_2_O/ACN = 90/10, 0–2 min, H_2_O/ACN = 10/90, 2–20 min, H_2_O/ACN = 10/90, 20–30 min).

#### 2-(2,3,4,6-Tetra-α-O-acetylglucopyranosyl)-8-hydroxynaphthalene-1,4-dione (**22**)

To a stirred solution of **17** (75 mg, 0.30 mmol) in DMF (6 mL) under the N_2_ atmosphere was added a solution of Ce_2_CO_3_ (91.5 mg, 0.30 mmol) and 2,3,4,6-tetra-*O*-acetyl-d-glucopyranose (103.5 mg, 0.30 mmol) in DMF (6 mL). After stirring for 4 h, the reaction mixture was concentrated under reduced pressure and purified by column chromatography (silica gel; EtOAc/hexane = 1/3) to give **22** (90 mg, 58%) as a yellow solid; *R*_f_ = 0.50 (EtOAc/hexane = 2/1); mp 214 °C; ^1^H NMR (400 MHz, CDCl_3_) δ 11.76 (s, 1H), 7.69–7.62 (m, 2H), 7.29–7.28 (m, 1H), 6.53 (s, 1H), 5.84 (d, 1H, *J* = 3.4 Hz), 5.74 (t, 1H, *J* = 9.2 Hz), 5.17 (t, 1H, *J* = 9.2 Hz), 5.10 (dd, 1H, *J* = 3.4 Hz, 10.2 Hz), 4.30 (dd, 1H, *J* = 5.60 Hz, 10.2 Hz), 4.07–4.04 (m, 2H), 2.12 (s, 3H), 2.07–2.06 (s, 9H) ppm; ^13^C NMR (100 MHz, CDCl_3_) δ 184.0, 183.8, 170.5, 170.3, 169.9, 169.5, 161.8, 156.4, 137.2, 131.6, 124.2, 119.0, 115.7, 114.2, 94.4, 69.8, 69.5, 69.0, 67.8, 61.3, 20.7 (3C), 20.6 ppm; HRMS (ESI-TOF MS) C_24_H_24_O_13_Na^+^ [M + Na]^+^ calc. 543.1109, found 543.1112; HPLC purity 95.3% (*t*_R_ 15.3 min, Hypersil BDS C18, 250 × 4.6 mm, 5 μm, 1 mL/min, H_2_O/ACN = 90/10, 0–2 min, H_2_O/ACN = 10/90, 2–20 min, H_2_O/ACN = 10/90, 20–30 min).

#### 2-(Dimethylamino)-8-hydroxynaphthalene-1,4-dione (**23**) and 3-(Dimethylamino)-8-hydroxynaphthalene-1,4-dione (**24**)

To a stirred suspension of **7** (700 mg, 4.0 mmol) in H_2_O (40 mL) was added dimethylamine_(2 M in THF)_ (4 mL). After stirring for 2 h, the reaction mixture was concentrated under reduced pressure and purified by column chromatography (silica gel; EtOAc/hexane = 1/7) to give **23** (174 mg, 20%) and **24** (50 mg, 6%) as red solids; *R*_f_ = 0.33 (**23**) and 0.55 (**24**) (EtOAc/hexane = 1/1); **23**: mp 160 °C; ^1^H NMR (600 MHz, CDCl_3_) δ 11.86 (s, 1H), 7.59–7.57 (m, 2H), 7.09–7.20 (m, 1H), 5.83 (s, 1H), 3.22 (s, 6H) ppm; ^13^C NMR (150 MHz, CDCl_3_) δ 188.3, 182.2, 161.7, 151.7, 136.9, 133.0, 122.6, 117.9, 115.4, 108.2, 43.1 ppm; HRMS (ESI-TOF MS) C_12_H_12_NO_3_^+^ [M + H]^+^ calc. 218.0812, found 218.0812; HPLC purity 95.2% (*t*_R_ 11.8 min, Hypersil BDS C18, 250 × 4.6 mm, 5 μm, 1 mL/min, H_2_O/ACN = 90/10, 0–2 min, H_2_O/ACN = 10/90, 2–20 min, H_2_O/ACN = 10/90, 20–30 min); **24**: mp 154 °C; ^1^H NMR (600 MHz, CDCl_3_) δ 12.43 (s, 1H), 7.48–7.43 (m, 2H), 7.18 (d, 1H, *J* = 7.9 Hz), 5.69 (s, 1H), 3.23 (s, 6H) ppm; ^13^C NMR (150 MHz, CDCl_3_) δ 188.0, 183.0, 160.4, 153.1, 133.8, 132.5, 124.4, 118.9, 114.7, 105.2, 42.9 ppm; HRMS (ESI-TOF MS) C_12_H_12_NO_3_^+^ [M + H]^+^ calc. 218.0812, found 218.0806; HPLC purity 96.8% (*t*_R_ 14.1 min, Hypersil BDS C18, 250 × 4.6 mm, 5 μm, 1 mL/min, H_2_O/ACN = 90/10, 0–2 min, H_2_O/ACN = 10/90, 2–20 min, H_2_O/ACN = 10/90, 20–30 min).

#### 2,8-Dihydroxynaphthalene-1,4-dione (**25**)

To a stirred solution of **23** (50 mg, 0.23 mmol) in 1,4-dioxane (1 mL) was added 10% HCl_(aq)_ (255 μL). After refluxing overnight, the reaction mixture was concentrated under reduced pressure and purified by column chromatography (silica gel; EtOAc/hexane = 1/2) to give **25** (40 mg, 91%) as an orange solid; *R*_f_ = 0.61 (EtOAc/hexane = 4/1); mp 198 °C; ^1^H NMR (400 MHz, CDCl_3_) δ 11.09 (s, 1H), 7.70–7.64 (m, 2H), 7.23 (dd, 1H, *J =* 1.8 Hz, 7.9 Hz), 6.35 (s, 1H) ppm; ^13^C NMR (100 MHz, CDCl_3_) δ 185.1, 184.0, 161.5, 156.0, 138.2, 132.6, 123.3, 119.5, 113.0, 111.5 ppm; HRMS (ESI-TOF MS) C_10_H_5_O_4_^–^ [M – H]^−^ calc. 189.0193, found 189.0188; HPLC purity 97.0% (*t*_R_ 14.0 min, Hypersil BDS C18, 250 × 4.6 mm, 5 μm, 1 mL/min, H_2_O/ACN = 90/10, 0–2 min, H_2_O/ACN = 10/90, 2–20 min, H_2_O/ACN = 10/90, 20–30 min).

#### 3,8-Dihydroxynaphthalene-1,4-dione (**26**)

To a stirred solution of **24** (70 mg, 0.32 mmol) in 1,4-dioxane (1 mL) was added 10% HCl_(aq)_ (350 μL). After refluxing overnight, the reaction mixture was concentrated under reduced pressure and purified by column chromatography (silica gel; EtOAc/hexane = 1/3) to give **26** (50 mg, 82%) as an orange solid; *R*_f_ = 0.61 (EtOAc/hexane = 4/1); mp 210 °C; ^1^H NMR (400 MHz, CDCl_3_) δ 12.32 (s, 1H), 7.69–7.67 (dd, 1H), 7.61–7.57 (t, 1H), 7.49 (bs, 1H), 7.34–7.32 (dd, 1H), 6.31(s, 1H); ppm; ^13^C NMR (100 MHz, CDCl_3_) δ 191.3, 181.2, 161.4, 157.0, 135.2, 129.3, 126.8, 119.8, 114.5, 110.4 ppm; HRMS (ESI-TOF MS) C_10_H_5_O_4_^–^ [M – H]^−^ calc. 189.0194, found 189.0193; HPLC purity 99.5% (*t*_R_ 3.0 min, Hypersil BDS C18, 250 × 4.6 mm, 5 μm, 1 mL/min, H_2_O/ACN = 90/10, 0–2 min, H_2_O/ACN = 10/90, 2–20 min, H_2_O/ACN = 10/90, 20–30 min).

#### 2,8-Dihydroxy-3-((4-methoxyphenyl)thio)naphthalene-1,4-dione (**27**)

To a stirred solution of **25** (50 mg, 0.26 mmol) in ethanol (1.5 mL) under the N_2_ atmosphere at −50 °C was added 4-methoxybenzenethiol (32 μL, 0.26 mmol) predissolved in ethanol (1.5 mL) and warmed to room temperature gradually. After stirring overnight, the reaction mixture was concentrated under reduced pressure and purified by column chromatography (silica gel; EtOAc/hexane = 2/1) to give **27** (15 mg, 18%) as a brown solid; *R*_f_ = 0.55 (EtOAc/hexane/acetic acid = 2/1/0.1); mp 224 °C; ^1^H NMR (400 MHz, CDCl_3_) δ 11.11 (s, 1H), 7.64–7.63 (m, 2H), 7.51–7.40 (m, 2H), 7.20 (dd, 1H, *J* = 2.5 Hz, 7.1 Hz), 6.86–6.79 (m, 2H), 3.79 (s, 3H) ppm; ^13^C NMR (100 MHz, CDCl_3_) δ 182.5, 180.6, 161.6, 159.8, 154.8, 137.6, 134.5 (2C), 132.7, 123.7, 122.8, 120.5, 115.8, 114,5 (2C), 113.0, 55.3 ppm; HRMS (ESI-TOF MS) C_17_H_11_O_5_S^–^ [M – H]^−^ calc. 328.0478, found 328.0478; HPLC purity 95.9% (*t*_R_ 18.5 min, Hypersil BDS C18, 250 × 4.6 mm, 5 μm, 1 mL/min, H_2_O/ACN = 90/10, 0–2 min, H_2_O/ACN = 10/90, 2–20 min, H_2_O/ACN = 10/90, 20–30 min).

#### 3,8-Dihydroxy-2-((4-methoxyphenyl)thio)naphthalene-1,4-dione (**28**)

To a stirred solution of **26** (153 mg, 0.80 mmol) and acetic acid (10 μL) in ethanol (4.5 mL) was added 4-methoxybenzenethiol (98.3 μL, 0.80 mmol) predissolved in ethanol (4.5 mL) under the N_2_ atmosphere. After stirring overnight, the reaction mixture was concentrated under reduced pressure and purified by column chromatography (silica gel; EtOAc/hexane = 1/3) to give **28** (61 mg, 23%) as a brown solid; *R*_f_ = 0.33 (EtOAc/hexane/acetic acid = 1/1/0.1); mp 219 °C; ^1^H NMR (400 MHz, CDCl_3_) δ 12.22, (s, 1H), 7.65 (d, 1H, *J* = 7.2 Hz), 7.55 (t, 1H, *J* = 8.1 Hz), 7.45 (d, 2H, *J* = 8.4 Hz), 7.29 (d, 1H, J = 8.4 Hz), 6.82 (d, 2H, *J* = 8.4 Hz), 3.78 (s, 3H) ppm; ^13^C NMR (100 MHz, CDCl_3_) δ 187.6, 178.9, 161.6, 159.7, 156.6, 135.4, 134.2 (2C), 129.4, 126.5, 122.7, 120.5, 119.9, 114.6 (2C), 114.5, 55.3 ppm; HRMS (ESI-TOF MS) C_17_H_12_O_5_SNa^+^ [M + Na]^+^ calc. 351.0298, found 351.0291; HPLC purity 97.3% (*t*_R_ 19.9 min Hypersil BDS C18, 250 × 4.6 mm, 5 μm, 1 mL/min, H_2_O/ACN = 90/10, 0–2 min, H_2_O/ACN = 10/90, 2–20 min, H_2_O/ACN = 10/90, 20–30 min).

#### 2-(2,4-Dihydroxyphenyl)-8-hydroxynaphthalene-1,4-dione (**29**)

To a stirred solution of **7** (400 mg, 2.3 mmol) in acetic acid (4 mL) was added a solution of resorcinol (126 mg, 1.2 mmol) in acetic acid (8 mL) and 2 M H_2_SO_4(aq)_ (2 mL). After stirring for 3 h, the reaction mixture was concentrated under reduced pressure and purified by column chromatography (silica gel; EtOAc/hexane = 1/3) to give **29** (167 mg, 26%) as a black red solid; *R*_f_ = 0.50 (EtOAc/hexane = 1/1); mp 213 °C; ^1^H NMR (600 MHz, (CD_3_)_2_SO) δ 11.94 (s, 1H), 9.69 (d, 2H, *J* = 8.4 Hz), 7.75 (t, 1H, *J* = 7.7 Hz), 7.53 (dd, 1H, *J* = 0.8 Hz, 7.7), 7.35 (dd, 1H, *J* = 0.8 Hz, 8.4), 7.08 (d, 1H, *J* = 8.4 Hz), 6.97 (s, 1H), 6.39 (d, 1H, *J* = 2.3), 6.31 (dd, 1H, *J* = 2.3 Hz, 8.4) ppm; ^13^C NMR (150 MHz, (CD_3_)_2_SO) δ 189.6, 184.6, 160.9, 160.4, 157.2, 150.0, 147.6, 137.1, 136.3, 132.7, 132.4, 118.3, 115.7, 111.7, 107.0, 103.1 ppm; HRMS (ESI-TOF MS) C_16_H_10_O_5_^–^ [M – H]^−^ calc. 283.0601, found 283.0613; HPLC purity 96.7% (*t*_R_ 12.6 min Hypersil BDS C18, 250 × 4.6 mm, 5 μm, 1 mL/min, H_2_O/ACN = 90/10, 0–2 min, H_2_O/ACN = 10/90, 2–20 min, H_2_O/ACN = 10/90, 20–30 min).

#### 2-(2,4-Dihydroxyphenyl)-3-((4-methoxyphenyl)thio)-8-hydroxynaphthalene-1,4-dione (**30**)

To a stirred solution of **29** (100 mg, 0.35 mmol) in ethanol (2 mL) was added 4-methoxybenznethiol (43 μL, 0.35 mmol) predissolved in ethanol (2 mL) under the N_2_ atmosphere at −20 °C. After stirring for 2 h, the reaction mixture was concentrated under reduced pressure and purified by column chromatography (silica gel; EtOAc/hexane = 1/2) to give **30** (98 mg, 67%) as a dark purple solid; *R*_f_ = 0.33 (EtOAc/hexane = 1/1); mp 236 °C; ^1^H NMR (600 MHz, (CD_3_)_2_CO) δ 12.80 (s, 1H), 8.66 (s, 2H), 7.55–7.51 (m, 2H), 7.23 (d, 1H, *J* = 8.3 Hz), 7.16 (d, 1H, *J* = 9.3 Hz), 7.12–7.10 (m, 3H), 7.05 (d, 1H, *J* = 9.3 Hz), 6.53 (d, 1H, *J* = 2.3 Hz), 6.49 (dd, 1H, *J* = 8.3 Hz, 2.3 Hz), 3.90 (s, 3H) ppm; ^13^C NMR (150 MHz, (CD_3_)_2_CO) δ 189.5, 183.7, 161.1, 160.2, 160.0, 156.5, 145.6, 137.5 (3C), 137.4, 134.4, 132.3, 125.2, 124.2, 122.2, 115.6 (2C), 112.0, 107.0, 54.96 ppm; HRMS (ESI-TOF MS) C_23_H_17_O_6_S^+^ [M + H]^+^ calc. 421.0740, found 421.0739; HPLC purity 96.7% (*t*_R_ 16.4 min, Hypersil BDS C18, 250 × 4.6 mm, 5 μm, 1 mL/min, H_2_O/ACN = 90/10, 0–2 min, H_2_O/ACN = 10/90, 2–20 min, H_2_O/ACN = 10/90, 20–30 min).

#### 2-(2,4-Dihydroxyphenyl)-3-(2,3,4,6-tetra-*O*-acetyl-1-thio-β-d-glucopyranose)-8-hydroxynaphthalene-1,4-dione (**31**)

To a stirred solution of **29** (20 mg, 0.071 mmol) in ethanol (1 mL) under the N_2_ atmosphere at −20 °C was added a solution of 2,3,4,6-tetra-*O*-acetyl-1-thio-d-glucopyranose (27.4 mg, 0.075 mmol) predissolved in ethanol (1 mL). After stirring for 2 h, the reaction mixture was concentrated under reduced pressure and purified by column chromatography (silica gel; EtOAc/hexane = 1/3) to give **31** (10 mg, 22%) as a dark purple solid; *R*_f_ = 0.50 (EtOAc/hexane = 1/1); mp 227 °C; ^1^H NMR (400 MHz, (CD_3_)_2_CO) δ 12.14 (s,1H), 8.75 (s, 2H), 7.77 (t, *J* = 7.4 Hz, 1H), 7.59 (d, *J* = 7.4 Hz, 1H), 7.31 (d, *J* = 8.2 Hz, 1H), 7.21 (d, *J* = 8.2 Hz, 1H), 6.52 (d, 1H, 2.3 Hz), 6.48 (d, *J* = 2.3 Hz, 1H), 6.46 (d, 1H, *J* = 2.3 Hz), 5.27–5.23 (m, 1H), 5.04 (t, *J* = 9.9, 1H), 4.90–4.88 (m, 1H), 4.24–4.19 (m, 1H), 4.10–4.06 (m, 1H), 3.99–3.94 (m, 1H), 2.02 (s, 3H), 2.01 (s, 3H), 1.99 (s, 3H), 1.94 (s, 3H) ppm; ^13^C NMR (100 MHz, (CD_3_)_2_CO) δ 190.9, 184.9, 170.4, 170.3, 170.2, 170.0, 162.3, 161.0, 157.5, 148.1, 137.7, 137.3, 133.2, 124.5, 118.7, 116.4, 113.0, 108.0, 103.8, 90.8, 79.8, 76.6, 75.7, 71.0, 70.0, 69.5, 69.2, 64.4, 63.8, 62.8, 20.6 ppm; HRMS (ESI-TOF MS) C_30_H_27_O_14_S^–^ [M – H]^−^ calc. 643.1127, found 643.1117; HPLC purity 96.7% (*t*_R_ 12.4 min, Hypersil BDS C18, 250 × 4.6 mm, 5 μm, 1 mL/min, H_2_O/ACN = 90/10, 0–2 min, H_2_O/ACN = 10/90, 2–20 min, H_2_O/ACN = 10/90, 20–30 min).

#### Cell Culture and Cytotoxicity Assay

The human cancer cell lines, lung adenocarcinoma A549, multiple myeloma RPMI8226, and glioma U87 were obtained from the American Tissue Culture Collection (ATCC, Manassas, VA, USA). Human breast adenocarcinoma MDA-MB-231, human embryonic kidney cells HEK-293T, and human vascular endothelial EA.hy926 cell lines were purchased from Bioresource Collection and Research Center (BCRC, Hsinchu, Taiwan). Cells were maintained in a humidified incubator at 37 °C in 5% CO_2_, and cell viability was determined with the resazurin assay (Cayman Chemical, USA) after the incubation with compounds for indicated concentrations and times or DMSO as control.^72^

#### Quantitative Reverse-Transcription Polymerase Chain Reaction (RT-qPCR)

Total RNA was extracted from A549, MDA-MB-231, and RPMI8226 cancer cells by GeneDireX Total RNA Isolation Kit (GeneDireX Inc., Taipei, Taiwan). The cDNA was synthesized by a High-Capacity cDNA Reverse Transcription Kit (Applied Biosystems, CA, USA). RT-qPCR was performed using a reaction mixture containing cDNA templates, primers, and KAPA SYBRFAST qPCR Master Mix in the Applied Biosystems StepOne Real-Time PCR System. Primer pairs used in RT-qPCR are listed below: PDI forward primer 5′- TCG AGT TCA CCG AGC AGA CAG −3′ and reverse primer 5′- AGC TCT CGG CTG CTG TTT TG −3′; β-actin forward primer 5′-TCA CCC ACA CTG TGC CCA TCT ACG A-3′ and reverse primer 5′-CAG CGG AAC CGC TCA TTG CCA ATG G-3′. The fold change in gene expression was calculated with normalization to β-actin values by a 2^–ΔΔCt^ comparative cycle threshold method.

#### Platelet Aggregation Assay

Washed human platelets were prepared from acid citrate dextrose-anticoagulated blood of healthy human volunteers as described previously.^73^ The study was approved by the Institutional Review Board of Kaohsiung Medical University, and informed consent was obtained from every volunteer. Platelet aggregation was measured by using a turbidimetric aggregometer (Chrono-Log Co., USA) under stirring conditions (1200 rpm) at 37 °C. The extent of platelet aggregation was measured as the maximal increase of light transmission after the addition of the stimulators U46619 and collagen.^73^ In the TCIPA assay, platelet aggregation was induced by A549 (1 × 10^4^ cells/mL), MDA-MB-231 (1 × 10^2^ cells/mL), or RPMI8226 (1 × 10^5^ cells/mL) cancer cells in the presence of 0.375% plasma.^74^

#### Measurement of Tumor Cell-Induced Platelet Secretion

Washed human platelets were coincubated with cancer cells under the same conditions as described in the TCIPA assay. After a 25 min incubation in the aggregometer, the samples were centrifuged at 13,000*g* for 1 min. The concentrations of PDGF-BB and ATP in the supernatants were determined using an ELISA kit (Abcam, Cambridge, UK) and an ATP bioluminescent assay kit (Sigma, MO, USA), respectively.^52^

#### Measurement of Platelet-Enhanced Tumor Cell Proliferation

A549 cells (1 × 10^4^ cells/well) were seeded in a 96-well plate and coincubated with wash human platelets (1 × 10^7^ cells/well) containing with 0.375% plasma for 48 h at 37 °C and 5% CO_2_ in a cell culture incubator. After washing with phosphate buffered saline, the numbers of viable cancer cells were determined by trypan blue exclusion.

#### PDI Inhibition Assay

PDI reductase activity was measured using the Di-E-GSSG assay.^75^ Washed human platelets (8 × 10^7^ /mL) or recombinant human PDI (20 nM) were incubated with test compounds in the presence of a nonfluorescent substrate Di-E-GSSG (150 nM). DTT (5 μM) was added to start the reaction, and the fluorescent product E-GSH (Ex 508 nm/Em 560 nm) was recorded for 60 min at 37 °C by a BioTek Synergy HT Microplate Reader (BioTek Instruments, VT, USA).

#### UPLC-MS Analysis of Compound Metabolites in High GSH Condition

Compounds (400 μM) were incubated in water (250 μL) with or without GSH (4 mM) at 37 °C for 30 min. The compound mixture was diluted with methanol (250 μL) and used for UPLC-MS analysis Metabolite analysis was performed on an ACQUITY UPLC I-Class/SQ Detector 2 (Waters, Milford, USA). The reverse phase BEH C18 column (100 mm × 2.1 mm, 1.7 μm, Waters) and VanGuard BEH C18 guard column (5 mm × 2.1 mm, 1.7 μm, Waters) were used. All data were acquired by Empower 3. The mobile phase for LC separation was Millipore water (A) and acetonitrile (B), and the program was as follows: 0.00 min 90% A → 0.50 min 90% A → 6.50 min 5% A → 8.50 min 5% A → 8.60 min 90% A → 9.00 min 90% A; at a rate of 0.4 mL/min for 9.00 min with 5 μL per injection. The temperature of the column oven was maintained at 40 °C. Mass spectrometer parameters were set as follows: cone voltage, 40.0 V, for positive/negative ion mode; desolvation temperature, 200 °C; cone gas flow(N_2_), 1 L/h; and desolvation gas flow (N_2_), 650 L/h.

#### Molecular Docking

The crystal structures of PDI protein were obtained from the PDB database with the PDB ID 6i7s, 4ekz, and 4el1.^57,58^ Structure issues of the PDI protein crystals were corrected using QuickPrep in MOE software, and the protein protonation pattern and minimum energy conformation were calculated by MOE under default parameters. The 3D structure of JUG was downloaded from The Cambridge Crystallographic Data Centre (https://www.ccdc.cam.ac.uk/), and the 3D structure of JUG derivatives was generated and optimized using conformer calculation in MarvinSketch (Chemaxon Ltd.) with the MMFF94 force field. MOE software was used to dock the JUG and JUG derivatives to PDI proteins. The binding site screening was performed on the pockets of covalent and substrate binding sites in PDI proteins as previously reported, which were 5 Å around Cys397, His354, and His256 respectively.^17,59,61^ The docking scores were set to 50 poses for London dG and 5 poses for GBVI/WSA dG. The covalent docking to catalytic Cys397 was performed by the Docktite function in MOE software.^76^ The disulfide bond in oxidized-PDI was removed artificially for covalent docking. The JUG and JUG derivatives were identified as Michael acceptors by the warhead screening function. The covalent docking process was performed by pharmacophore docking with the pharmacophore as the placement method and induced fit as the refinement method and the docking scores were both set to 100 poses for Affinity dG. The docking results were ordered by the binding energy using the S Score function, and the ligand interaction function in MOE was used to obtain the 2D interaction diagram.

## Statistical Analysis

Data are presented as the mean ± standard error of the mean (SEM), and statistical significance was calculated by one-way analysis of variance (ANOVA) using GraphPad Prism 8. The statistical significance was determined as follows: **P < 0.01; ***P < 0.001

## Supporting Information Available

Parallel artificial membrane permeability assay (PAMPA); UPLC-MS analysis of **30** and **31** after GSH treatment; PDI superimposition between PDI of 6i7s, 4ekz, and 4el1 and binding affinity comparison; ^1^H NMR, ^13^C NMR, HRMS, and HPLC purity spectra of synthesized compounds (PDF)
Molecular formula strings (CSV)
Binding mode of library docking of monosubstituted JUG derivatives with three binding pockets of 6i7s (PDB)
Binding mode of library docking of monosubstituted JUG derivatives with active site of 4el1 (PDB)
Binding mode of JUG with substrate binding pockets of 6i7s (PDB)
Binding mode of **18** with substrate binding pockets of 6i7s (PDB)
Covalent binding mode of JUG with active binding site Cys397 of 4el1 (PDB)
Covalent binding mode of **18** with active binding site Cys397 of 4el1 (PDB)
PDI protein superimposition between 6i7s and 4ekz (PDB)
PDI protein superimposition between 6i7s and 4el1 (PDB)

## Supplementary Material

jm3c02107_si_002.csv

jm3c02107_si_003.pdb

jm3c02107_si_004.pdb

jm3c02107_si_005.pdb

jm3c02107_si_006.pdb

jm3c02107_si_007.pdb

jm3c02107_si_008.pdb

jm3c02107_si_009.pdb

jm3c02107_si_010.pdb

## ABBREVIATIONS

ACN: acetonitrile
Di-E-GSSG: dieosin glutathione disulfide
E-GSH: eosin-glutathione
ER: endoplasmic reticulum
EtOAc: ethyl acetate
GPVI: glycoprotein VI
GSH: glutathione
GSSG: glutathione disulfide
MOE: molecular operating environment
NaOMe: sodium methoxide
PDGF: platelet-derived growth factor
PDI: protein disulfide isomerase
rPDI: recombinant PDI
TCIPA: tumor cell-induced platelet aggregation
TF: tissue factor
VEGF: vascular endothelia growth factor
