# Supplementary material for: Discovery of 5-Hydroxy-1,4-naphthoquinone (Juglone) Derivatives as Dual Effective Agents Targeting Platelet-Cancer Interplay through Protein Disulfide Isomerase Inhibition
Source: J Med Chem. 2024 Feb 21;67(5):3626–42. doi: 10.1021/acs.jmedchem.3c02107 (PMC10945480; doi:10.1021/acs.jmedchem.3c02107)
Supplement: Supplementary file 1 — jm3c02107_si_001.pdf [file jm3c02107_si_001.pdf]

Supporting Information

Discovery of 5-hydroxy-1,4-naphthoquinone (Juglone) Derivatives as Dual Effective Agents Targeting Platelet-Cancer Interplay through Protein Disulfide Isomerase Inhibition

Yu-Pu Juang[§,⊥], Ju-Ying Tsai[#,⊥], Wan-Lan Gu[§], Hui-Ching Hsu[#], Chao-Lung Lin[§], Chin-Chung Wu[#,*], and Pi-Hui Liang[§,†,*]

[§]School of Pharmacy, College of Medicine, National Taiwan University, Taipei 100, Taiwan.
[#] Graduate Institute of Natural Product, Kaohsiung Medical University, Kaohsiung 807, Taiwan.
†The Genomics Research Center, Academia Sinica, Taipei 128, Taiwan.
[⊥]These Arthur contributed equally to this work.

AUTHOR INFORMATION
*Corresponding Authors
E-mail: phliang@ntu.edu.tw; ccwu@kmu.edu.tw

Table of Contents

1. Parallel artificial membrane permeability assay (PAMPA) S3

2. UPLC-MS analysis of **30** and **31** after GSH treatment S4–8

3. PDI superimposition between PDI of 6i7s, 4ekz, and 4el1 and binding affinity comparison S9

4. ^{1}H NMR, ^{13}C NMR, HRMS, HPLC purity spectra of compounds S10–57

1. Parallel artificial membrane permeability assay (PAMPA).

Methods: The membrane permeability of JUG derivatives was determined using PAMPA evolution instruments from pION Inc. In the PAMPA assay, a set of sandwich plates consists of a 96-well microtiter donor plate at the bottom and a 96-well filter acceptor plate on the top from Millipore (IPVH, 125 μm thick filter, 0.45 μm pore). The stock solutions of samples were prepared at 10 mM concentrations in DMSO and stored at 0 ℃ before use. Before being added to a 96-well filter plate, the stock solution was diluted first with buffer to achieve a final sample concentration of 50 μM and to reduce the DMSO concentration below 1% (v/v). Each well of the 96-well donor plate was filled with a 200 μL diluted sample solution (50 μM), and the 96-well filter acceptor plate was then wetted with a 5 μL 2% w/v dioleyoylphosphatidylcholine (DOPC) in n-dodecane and subsequently placed on the donor wells. [10.1016/j.ejps.2005.12.008; 10.1016/j.ejps.2004.07.009] Immediately, the acceptor wells were filled with 200 μL of buffer solution and the PAMPA plate sandwich was sealed and incubated at 25 ℃ for 3 h. After reaching the permeation time, the PAMPA plate sandwich was separated and transferred to 96-well UV plates respectively. The amounts of drugs in both the donor and acceptor compartments were measured by comparing the experimental spectra with the UV spectrum (220~400 nm) obtained from reference standards.

Table S1. Membrane permeability of juglone derivatives determined by PAMPA.Verapamil and ranitidine were used as standard compound indicating 100% and 0% membrane permeability respectively.

Cmpds.	Permeability (%)	Cmpds.	Permeability (%)	Cmpds.	Permeability (%)
**Juglone**	83.43	**16**	91.9	**25**	5.21
**8**	80.43	**17**	16.9	**26**	5.17
**9**	38.9	**18**	10.64	**27**	1.08
**10**	28.4	**19**	-0.96	**28**	9.28
**11**	2.7	**20**	17.1	**29**	42.95
**12**	0.1	**21**	61.4	**30**	12.64
**13**	8.5	**22**	57.45	**31**	28.86
**14**	N.D.[a]	**23**	16.7	Verapamil	100
**15**	85.4	**24**	30.2	Ranitidine	0

[a] N.D.: not determined

2. UPLC-MS analysis of 30 and 31 after GSH treatment

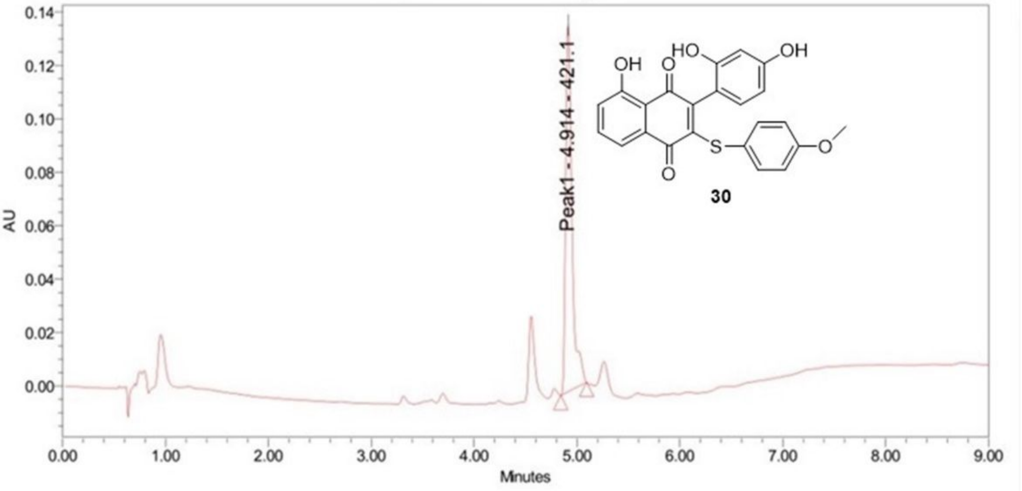

Peak Results

	Name	RT	Area	Height	Amount	Units
1	Peak1	4.914	577321	137409		

PDA Result Table

	Name	RT	Purity1 Angle	Purity1 Threshold	Match1 Spect. Name	Match1 Angle	Match1 Threshold
1	Peak1	4.914					

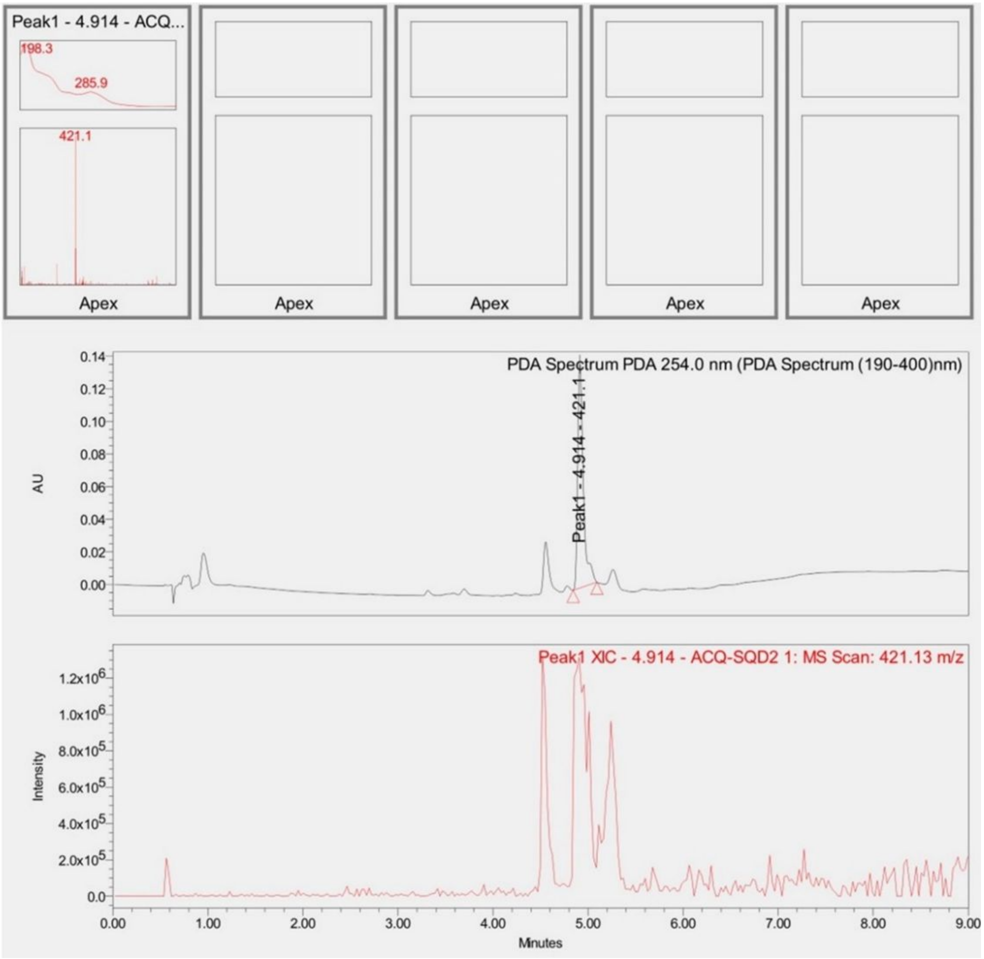

Fig. S1. UPLC-MS spectrum of compound **30**.

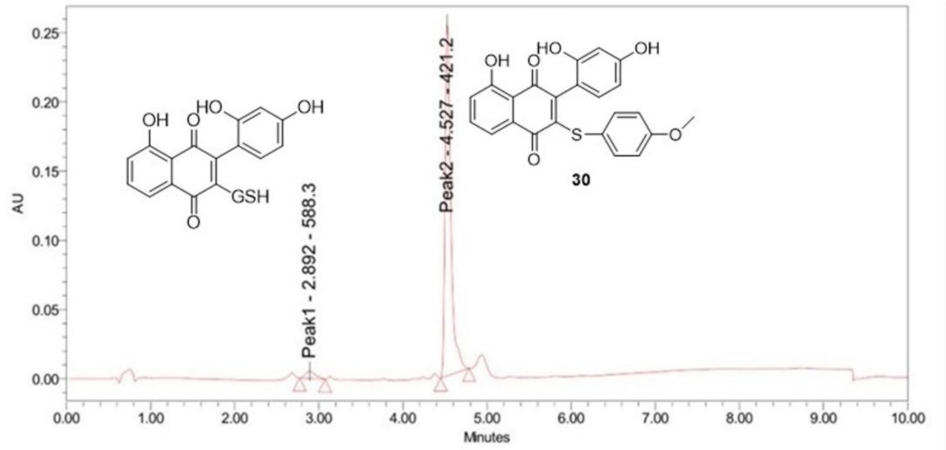

Peak Results

	Name	RT	Area	Height	Amount	Units
1	Peak1	2.892	43060	5202		
2	Peak2	4.527	1211275	254000		

PDA Result Table

	Name	RT	Purity1 Angle	Purity1 Threshold	Match1 Spect. Name	Match1 Angle	Match1 Threshold
1	Peak1	2.892					
2	Peak2	4.527					

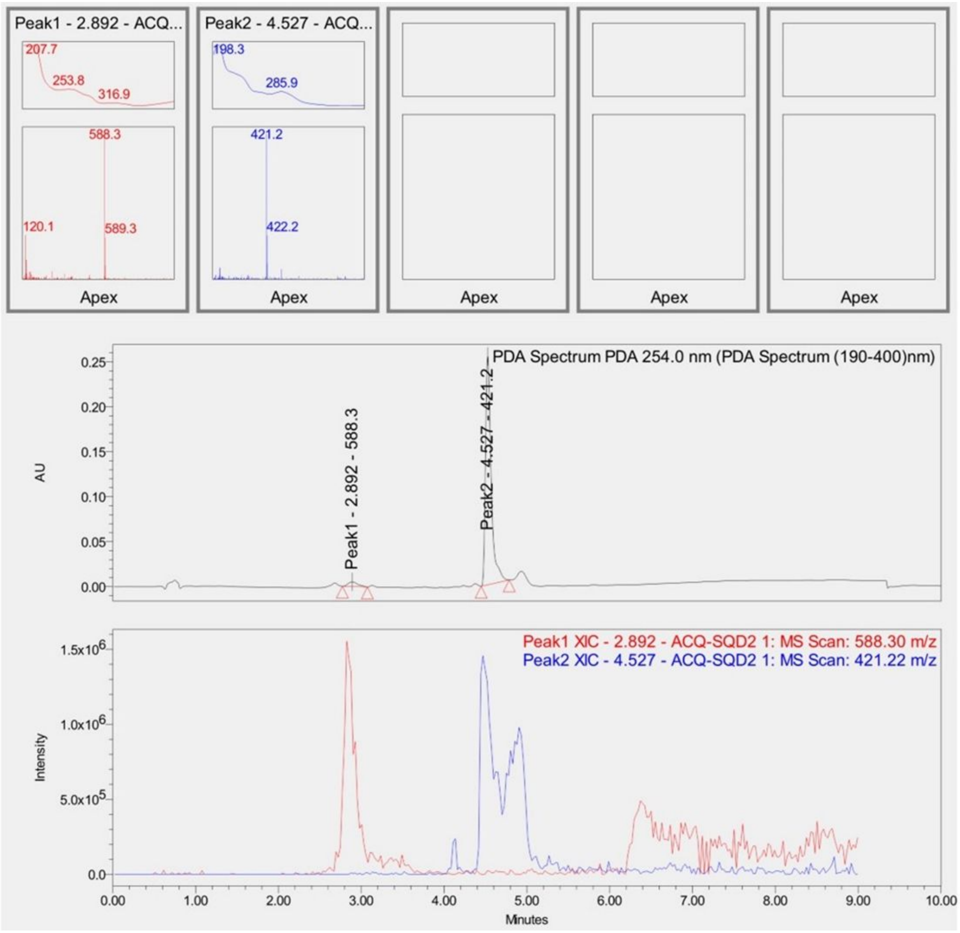

Fig. S2. UPLC-MS spectrum of compound **30** after GSH treatment.

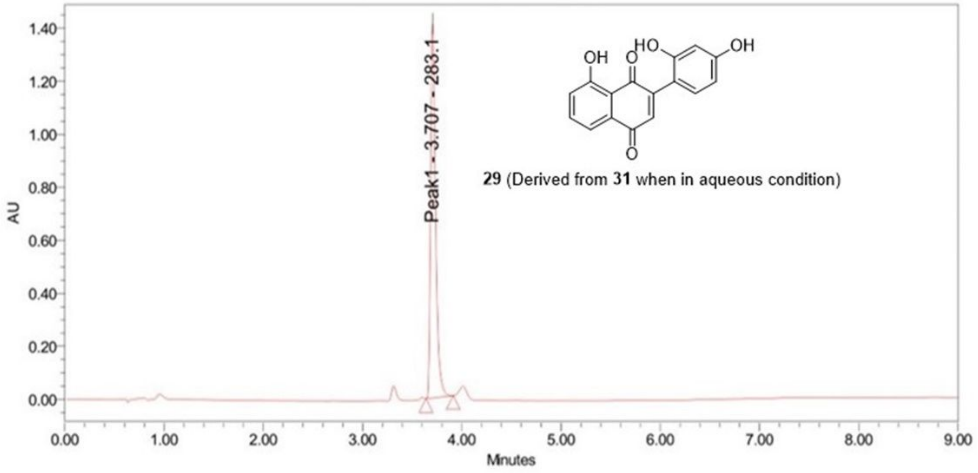

Peak Results

	Name	RT	Area	Height	Amount	Units
1	Peak1	3.707	4997129	1412482		

PDA Result Table

	Name	RT	Purity1 Angle	Purity1 Threshold	Match1 Spect. Name	Match1 Angle	Match1 Threshold
1	Peak1	3.707					

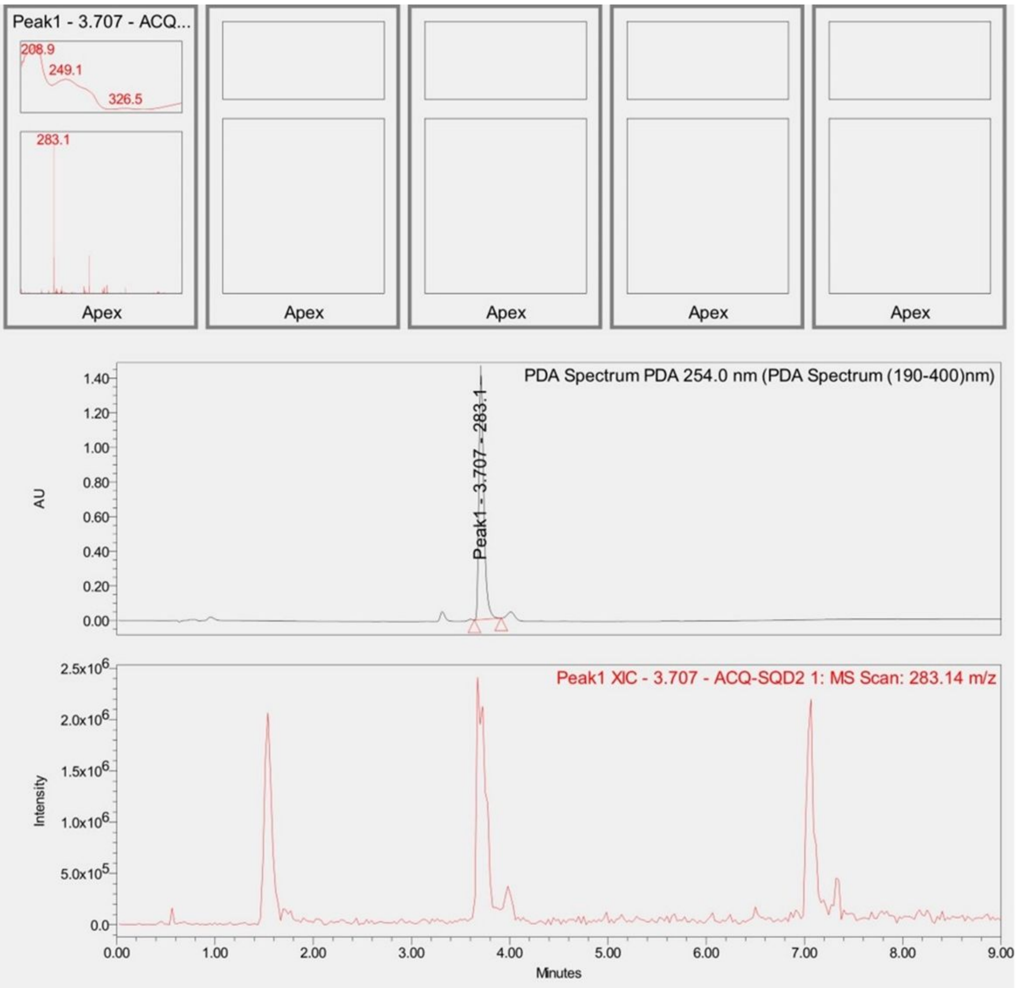

Fig. S3. UPLC-MS spectrum of compound **31**.

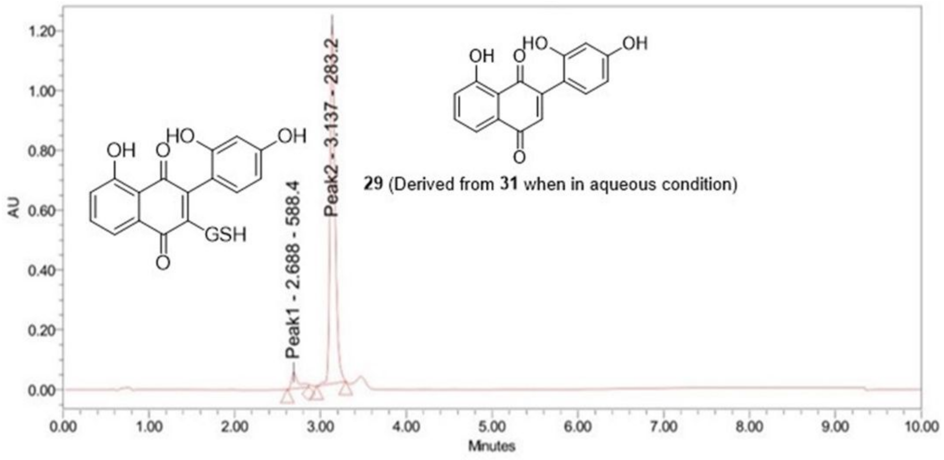

Peak Results

	Name	RT	Area	Height	Amount	Units
1	Peak1	2.688	293822	55088		
2	Peak2	3.137	4949547	1199488		

PDA Result Table

	Name	RT	Purity1 Angle	Purity1 Threshold	Match1 Spect. Name	Match1 Angle	Match1 Threshold
1	Peak1	2.688					
2	Peak2	3.137					

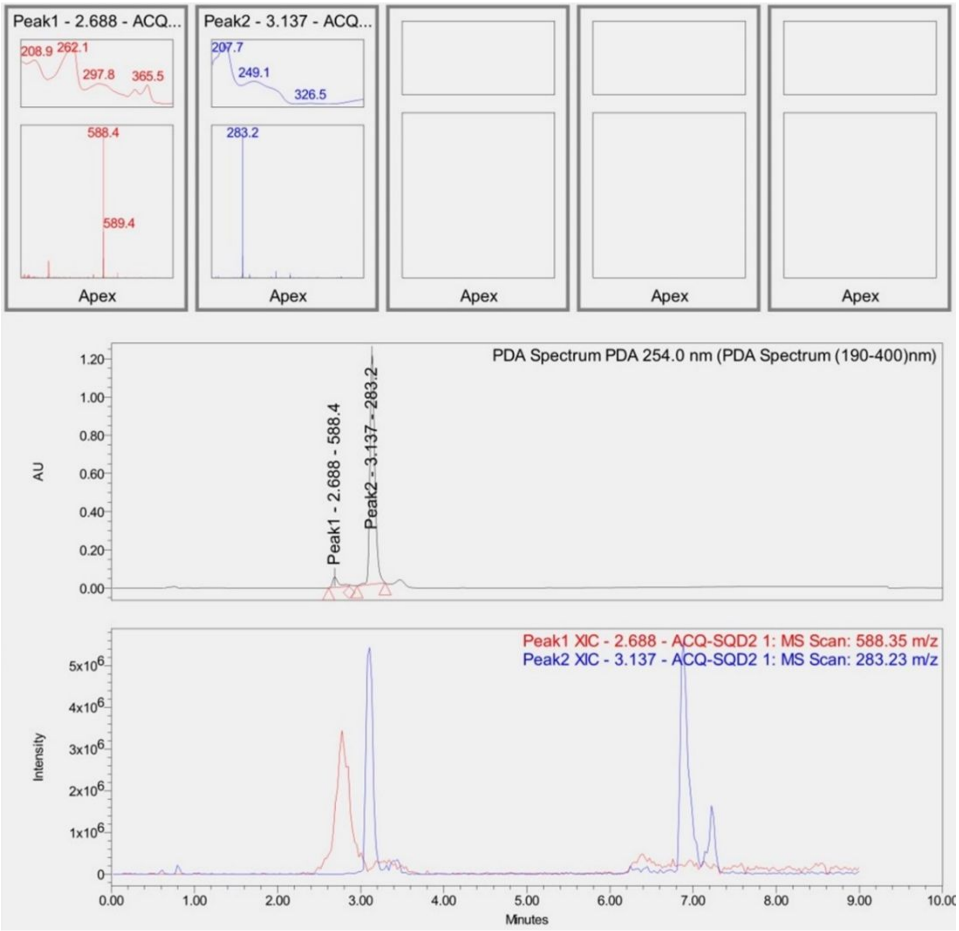

Fig. S4. UPLC-MS spectrum of compound **31** after GSH treatment.

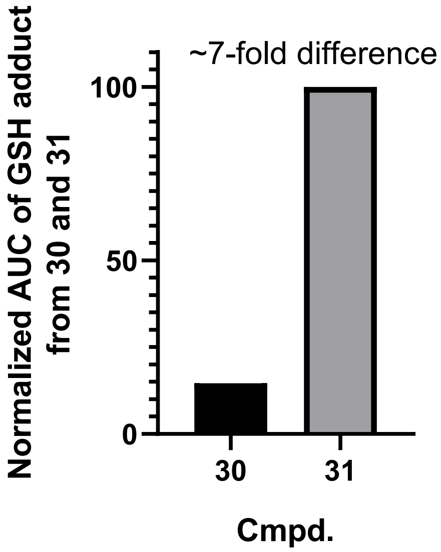

Fig. S5. Normalized area-under-curve data of GSH adduct in compound **30** and **31**. Data derived from area of peak 1 in Fig. S2 and S4.

3. PDI superimposition between PDI of 6i7s, 4ekz, and 4el1 and binding affinity comparison

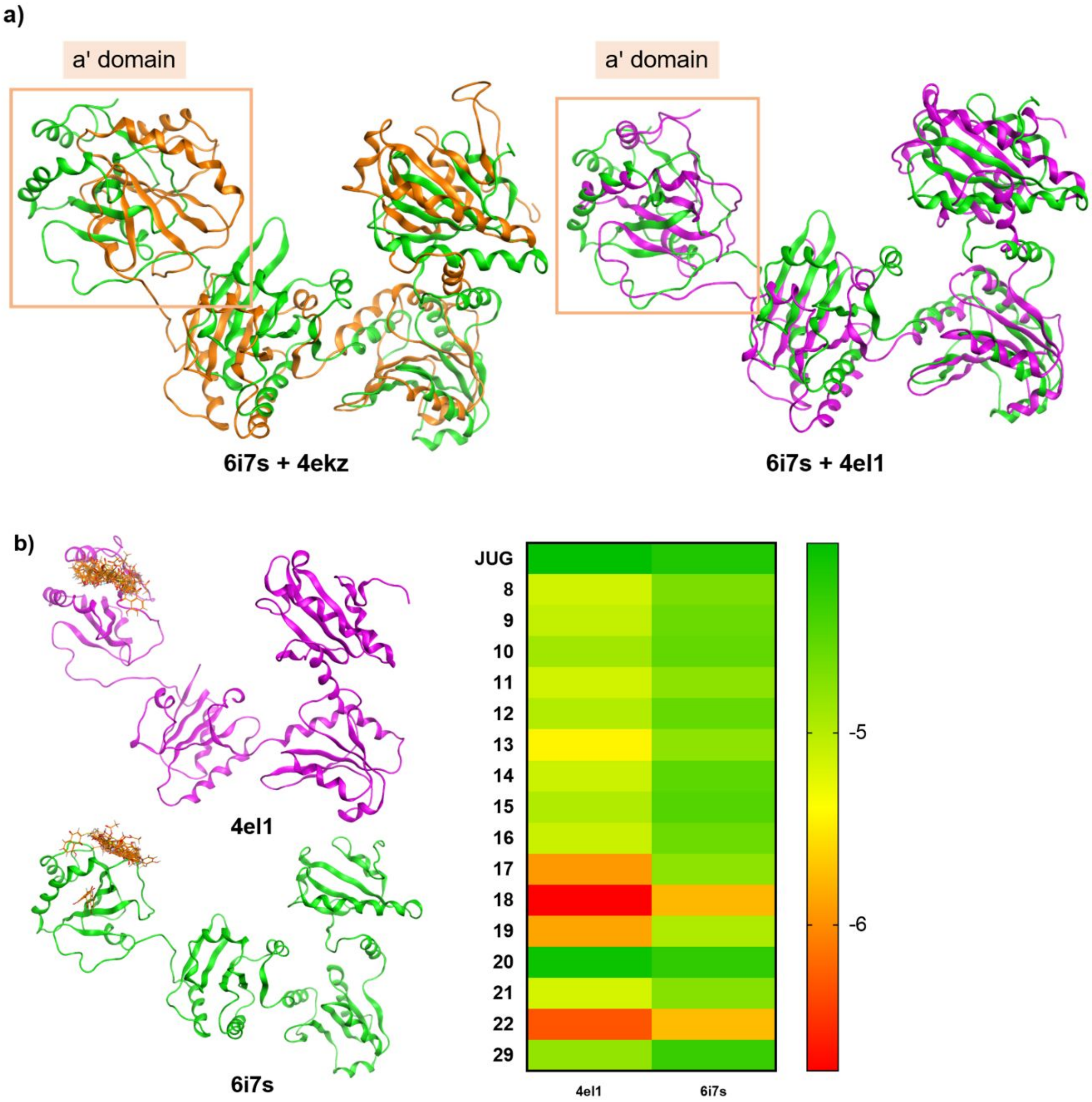

Fig. S6. (a) PDI protein superimposition between 6i7s, 4ekz, and 4el1. Green: 6i7s; Orange: 4ekz; Purple: 4el1. (b) Binding affinity heatmap of mono-substituted JUG derivatives binding to active site in oxidized-PDI (4el1) and reduced-PDI (6i7s).

4. ^{1}H NMR, ^{13}C NMR, HRMS, HPLC purity spectra of compounds

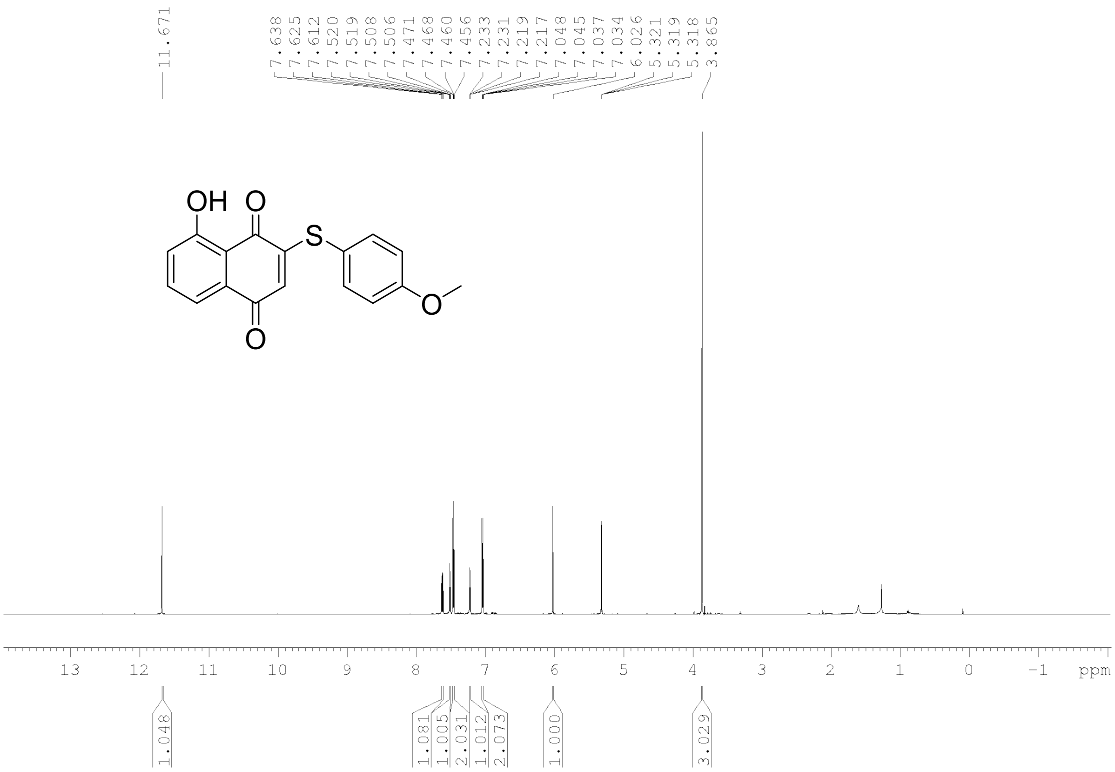

Fig. S7. ^{1}H NMR of **8** (CD_2Cl_2, 600 MHz)

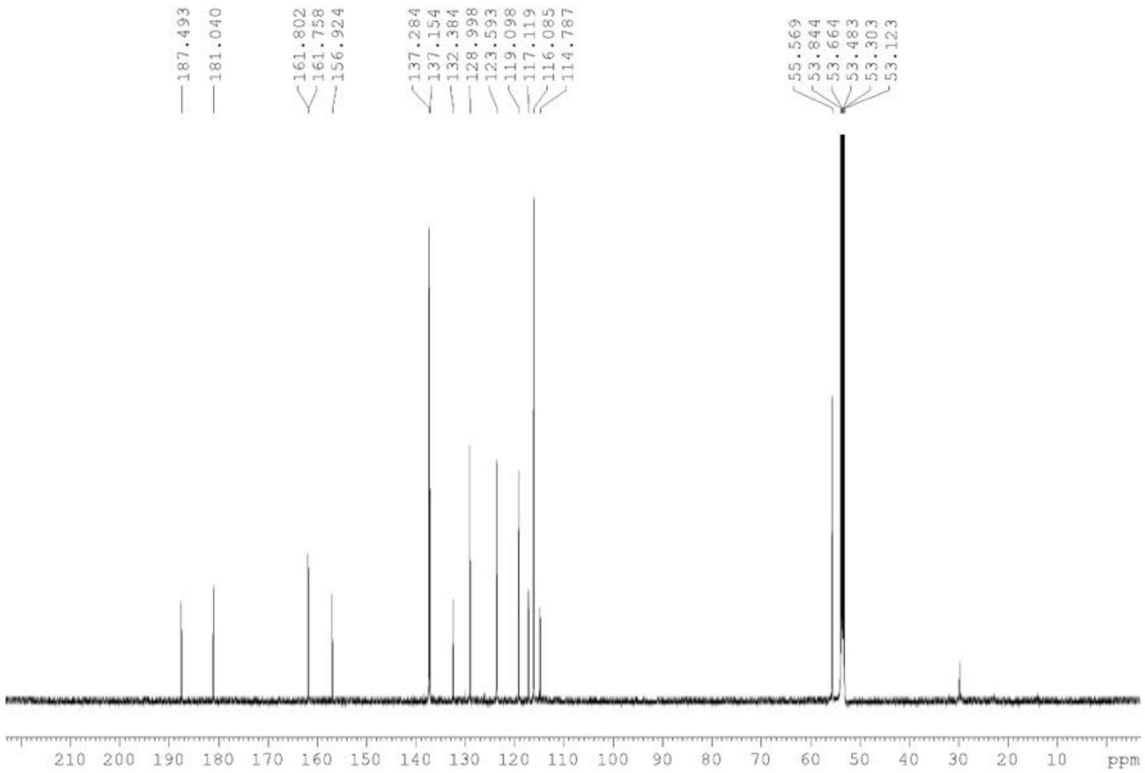

Fig. S8. ^{13}C NMR of **8** (CD_2Cl_2, 150 MHz)

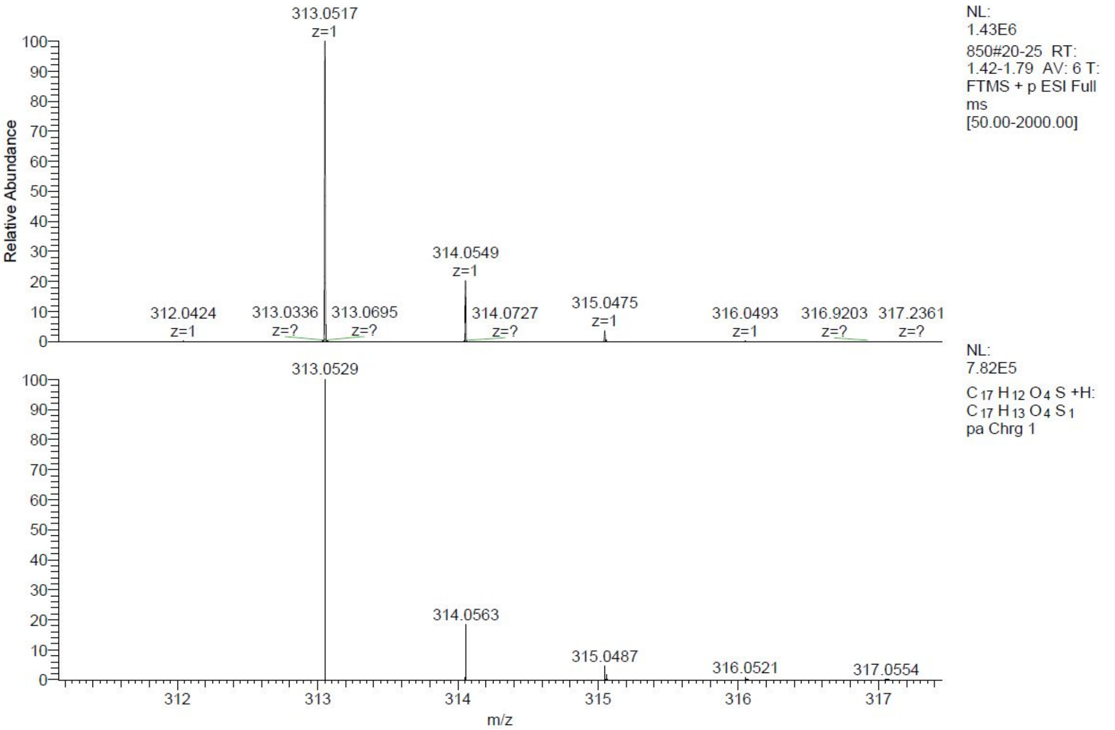

Fig. S9. HR-ESI-MS (+) spectrum of **8**. Upper: found MS; Lower: calculated MS.

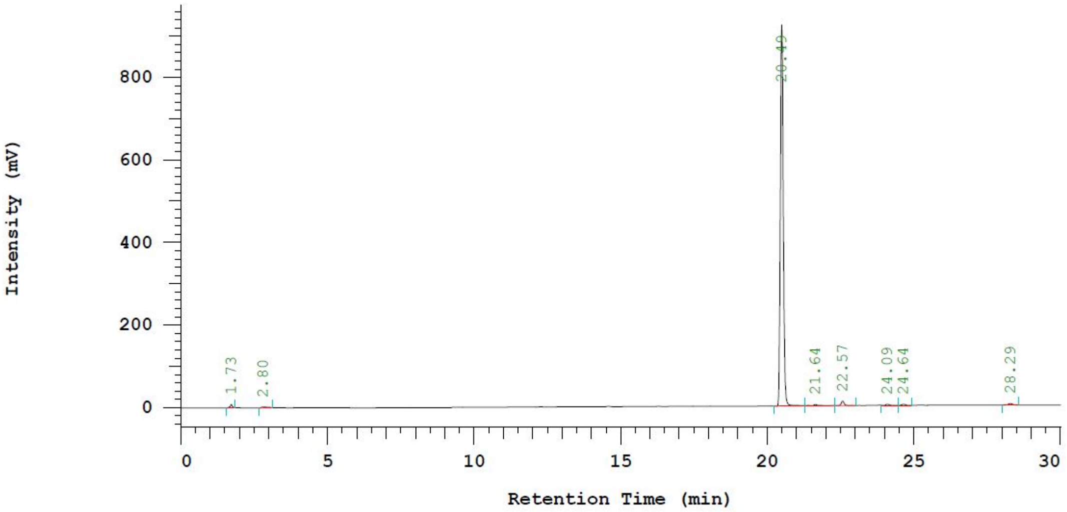

No.	RT	Area	Conc 1	BC
1	1.73	40052	0.605	BB
2	2.80	24009	0.363	BB
3	20.49	6354050	95.949	BB
4	21.64	43701	0.660	BB
5	22.57	81641	1.233	BB
6	24.09	18271	0.276	BB
7	24.64	22744	0.343	BB
8	28.29	37831	0.571	BB
		6622299	100.000	

Fig. S10. HPLC analysis of **8**.

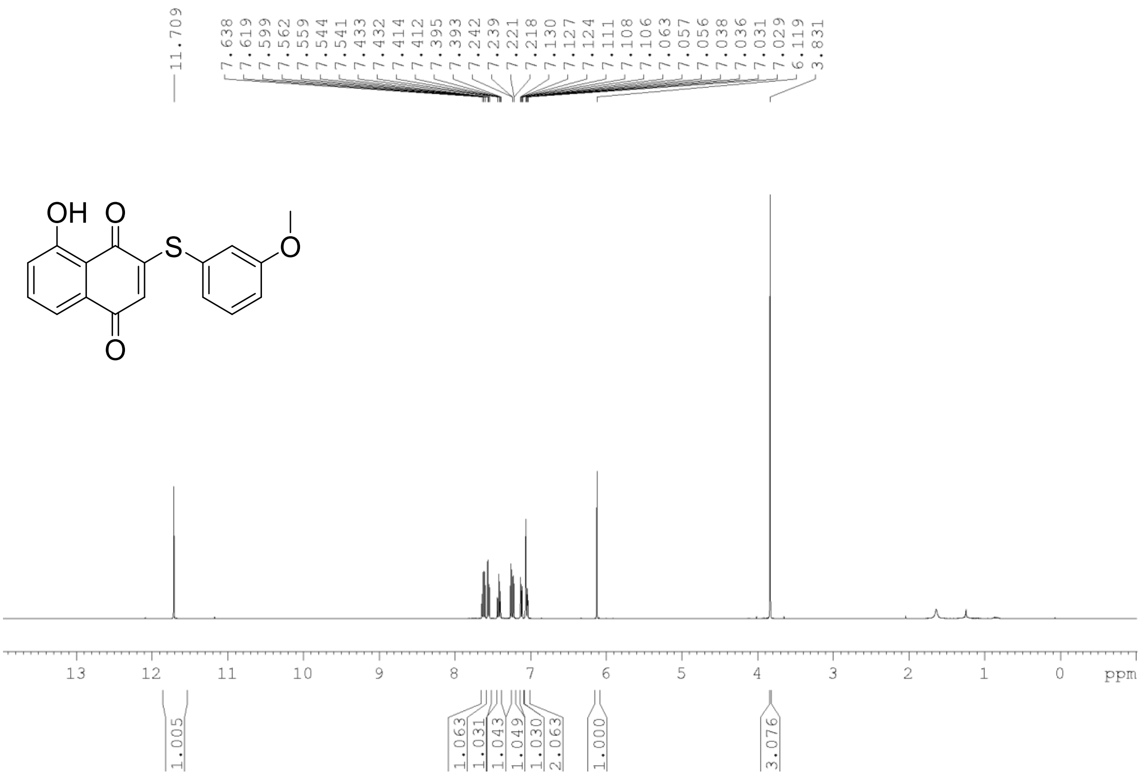

Fig. S11. ^{1}H NMR spectrum of **9** ($CDCl_3$, 400 MHz).

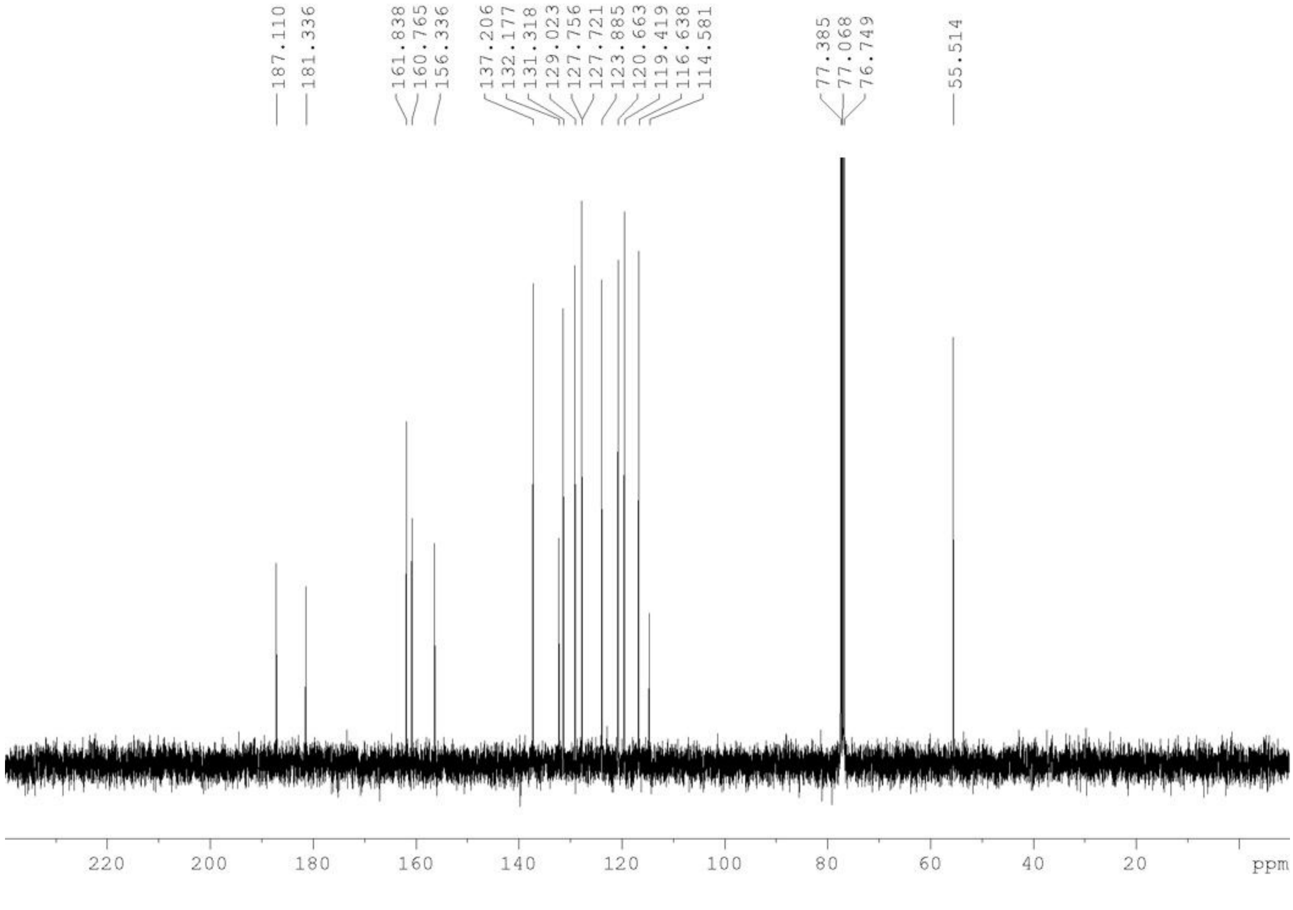

Fig. S12. ^{13}C NMR spectrum of **9** ($CDCl_3$, 100 MHz).

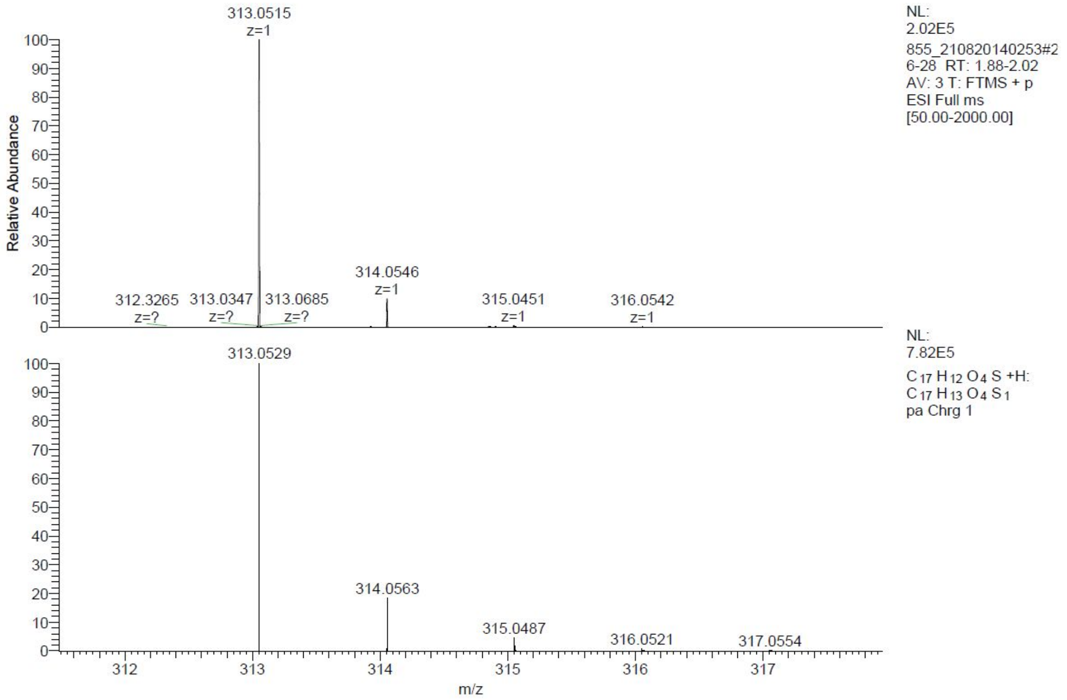

Fig. S13. HR-ESI-MS (+) spectrum of **9.** Upper: found MS; Lower: calculated MS.

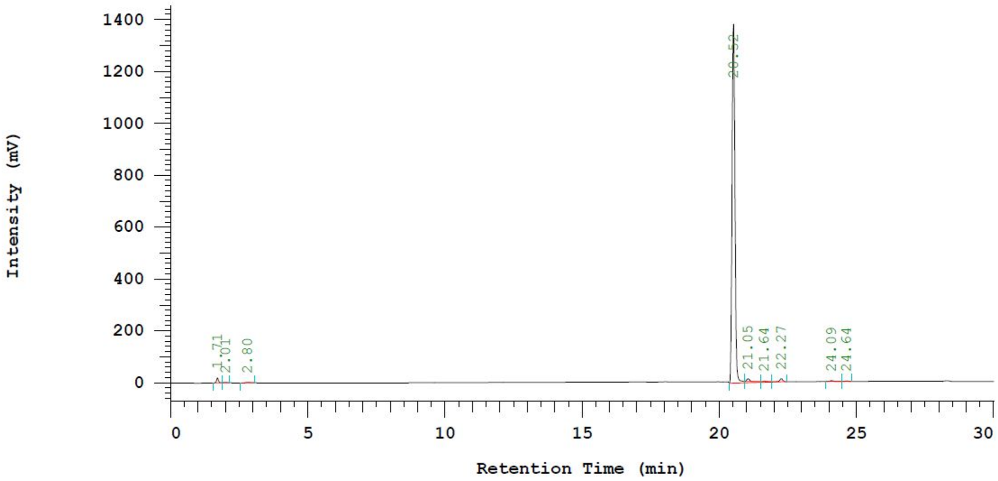

No.	RT	Area	Conc 1	BC
1	1.71	95413	0.900	BB
2	2.01	14075	0.133	BB
3	2.80	19318	0.182	BB
4	20.52	10247515	96.654	BV
5	21.05	69032	0.651	TBB
6	21.64	15218	0.144	TBB
7	22.27	105168	0.992	BB
8	24.09	24317	0.229	BB
9	24.64	12207	0.115	BB
		10602263	100.000	

Fig. S14. HPLC analysis of **9**.

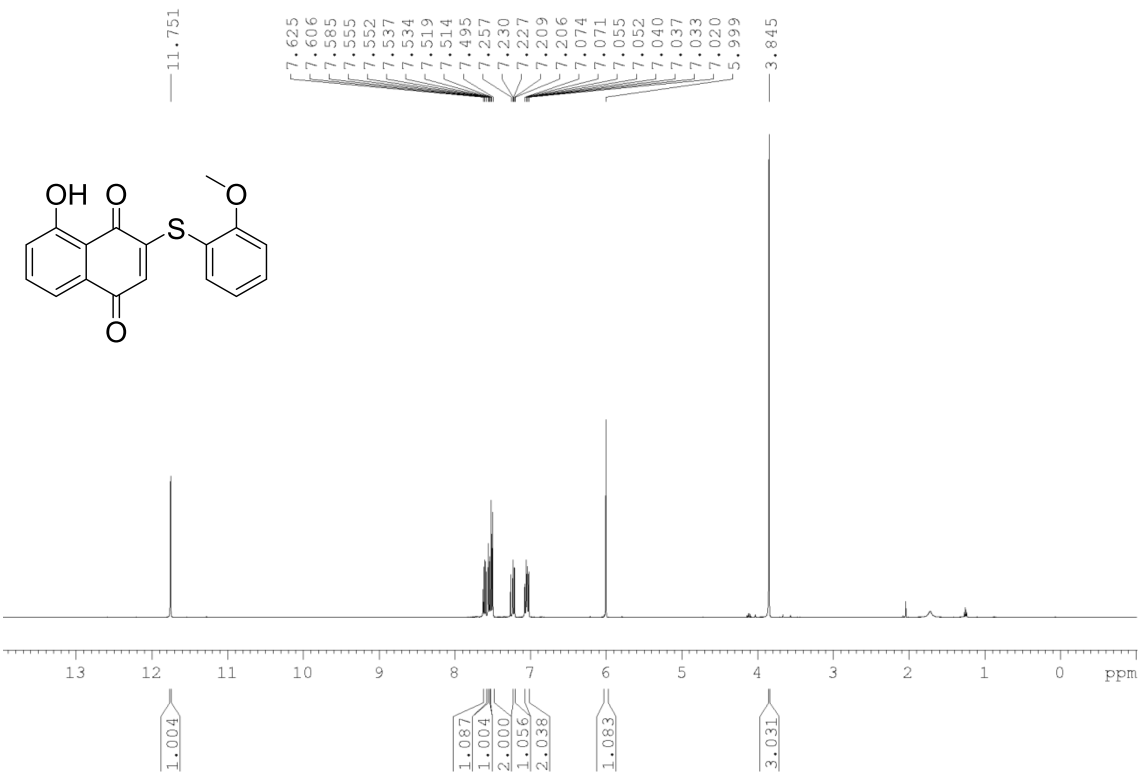

Fig. S15. ^{1}H NMR spectrum of **10** ($CDCl_3$, 600 MHz).

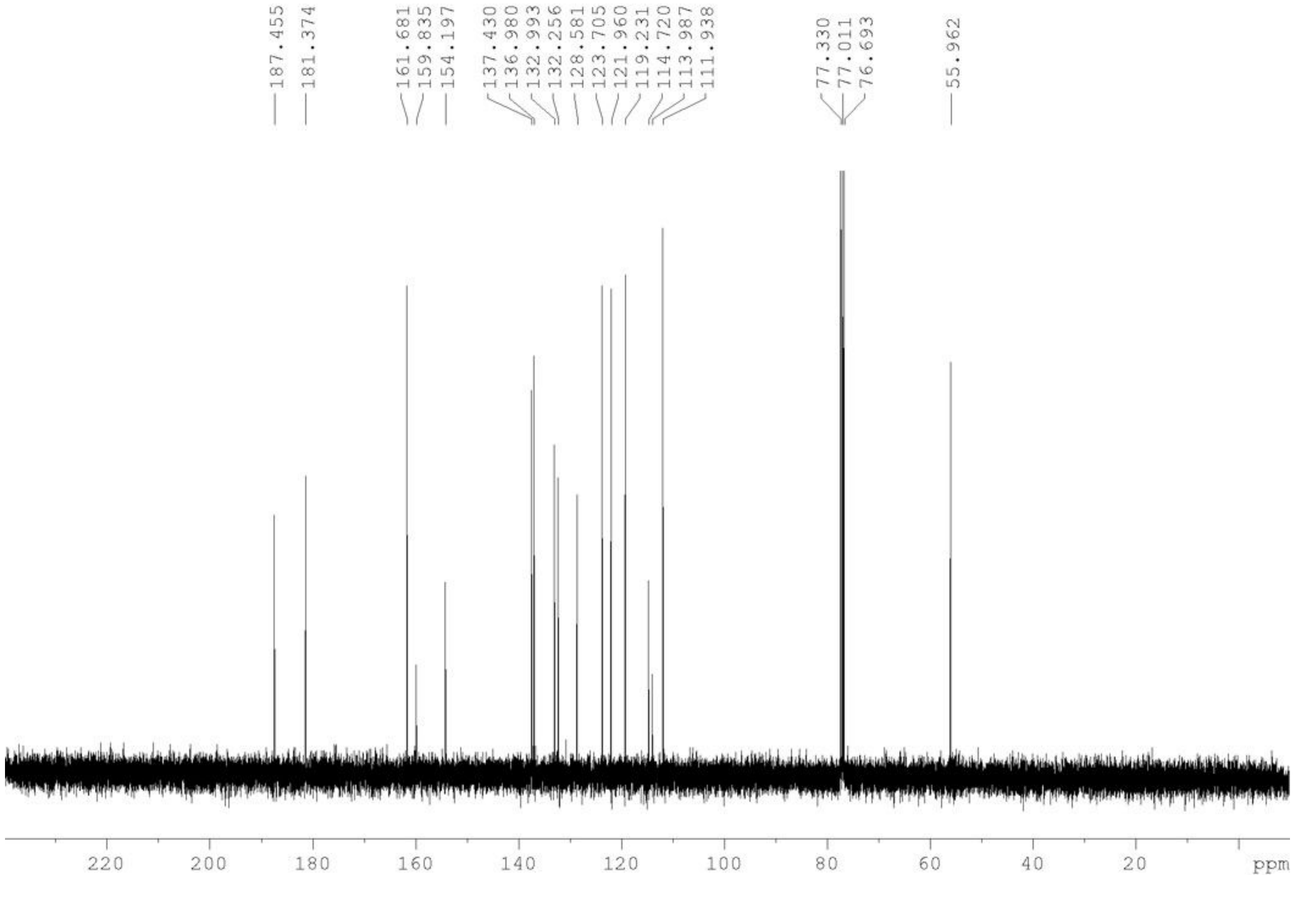

Fig. S16. ^{13}C NMR spectrum of **10** ($CDCl_3$, 150 MHz).

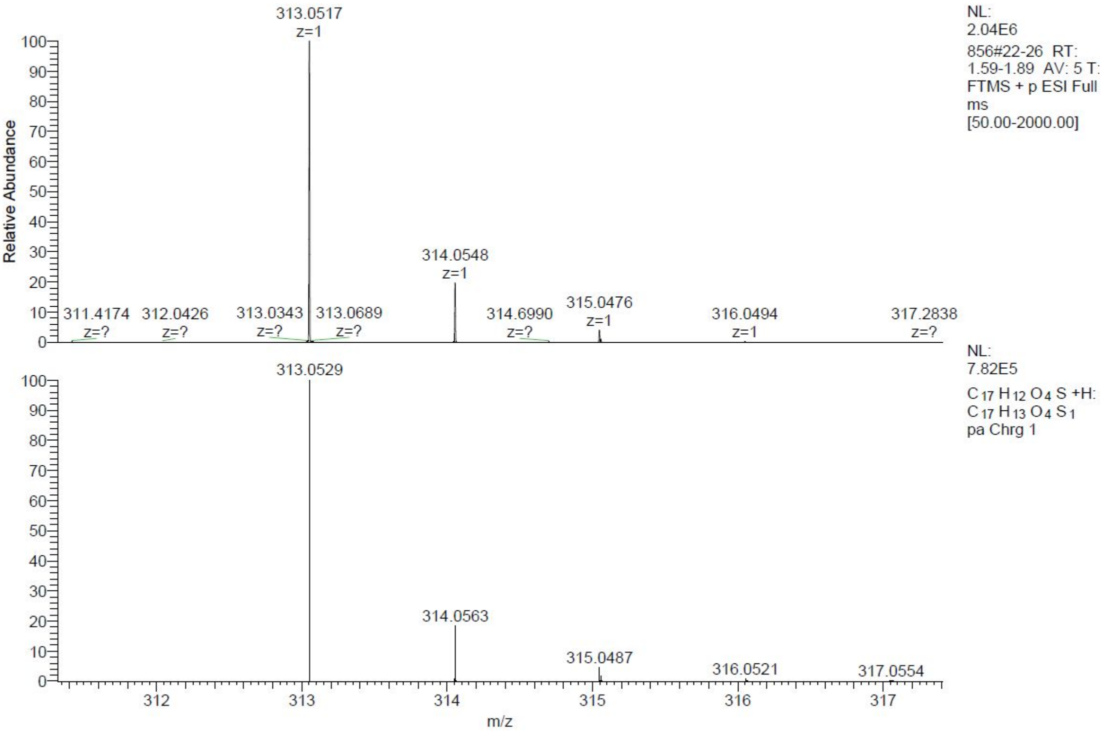

Fig. S17. HR-ESI-MS (+) spectrum of **10.** Upper: found MS; Lower: calculated MS.

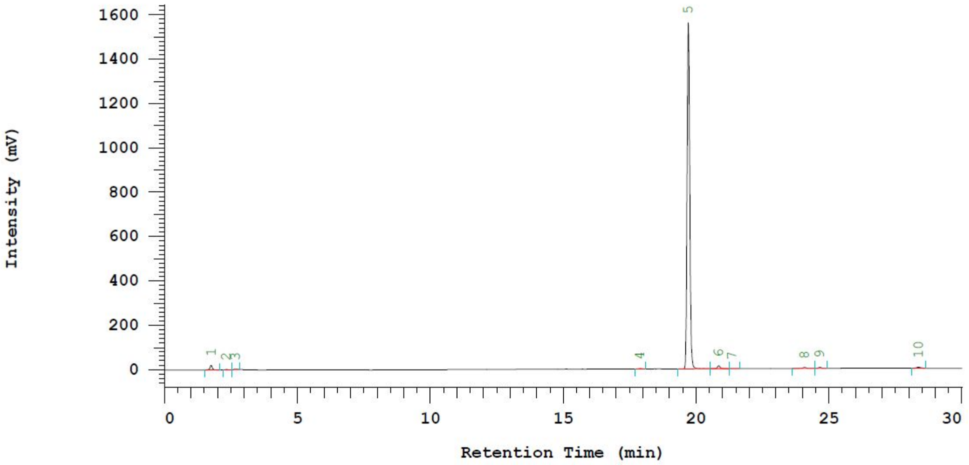

No.	RT	Area	Conc 1	BC
1	1.76	132426	1.177	BB
2	2.33	10538	0.094	BB
3	2.67	23993	0.213	BB
4	17.89	14950	0.133	BB
5	19.71	10836290	96.352	BB
6	20.85	89773	0.798	TBB
7	21.36	11898	0.106	BB
8	24.08	39791	0.354	BB
9	24.65	35003	0.311	BB
10	28.37	51846	0.461	BB
		11246508	100.000	

Fig. S18. HPLC analysis of **10**.

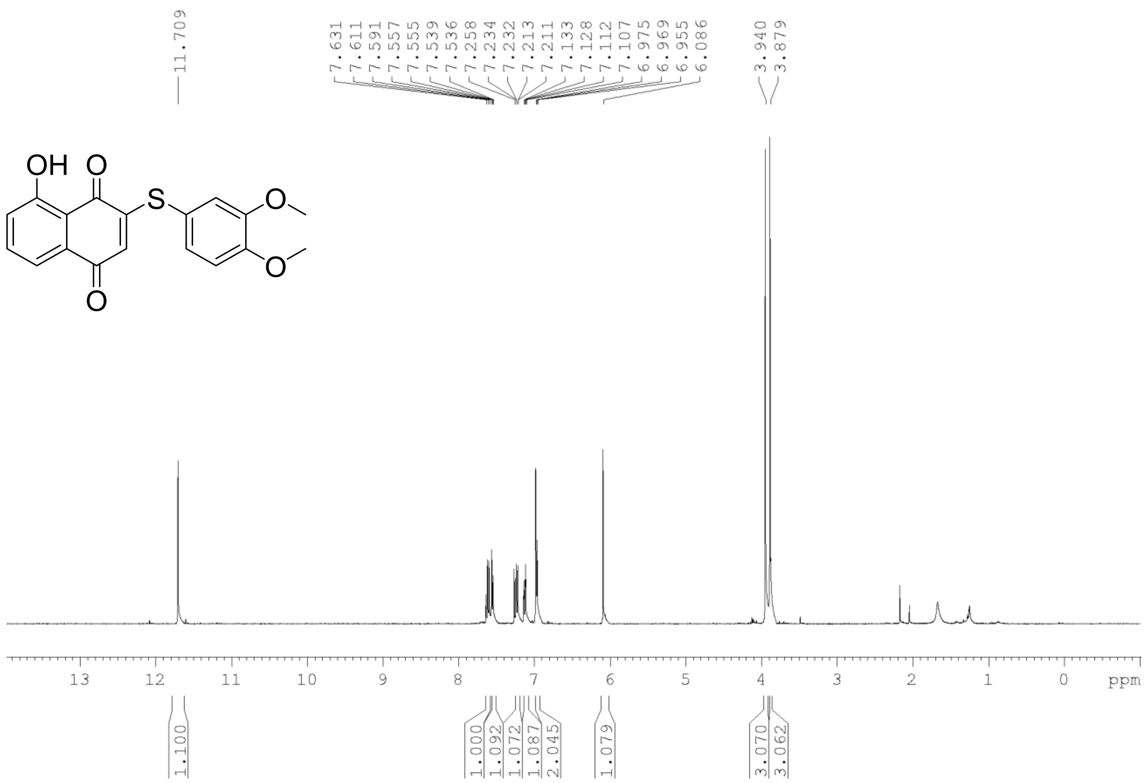

Fig. S19. ^{1}H NMR spectrum of **11** ($CDCl_3$, 400 MHz).

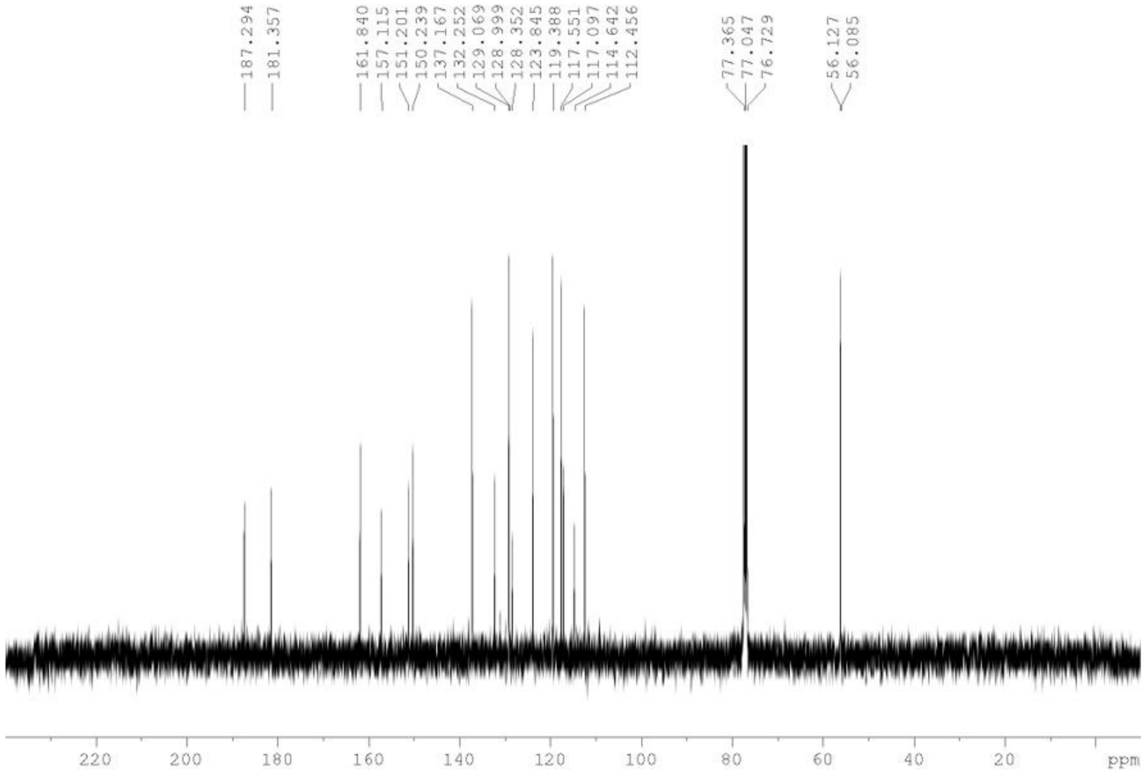

Fig. S20. ^{13}C NMR spectrum of **11** ($CDCl_3$, 100 MHz).

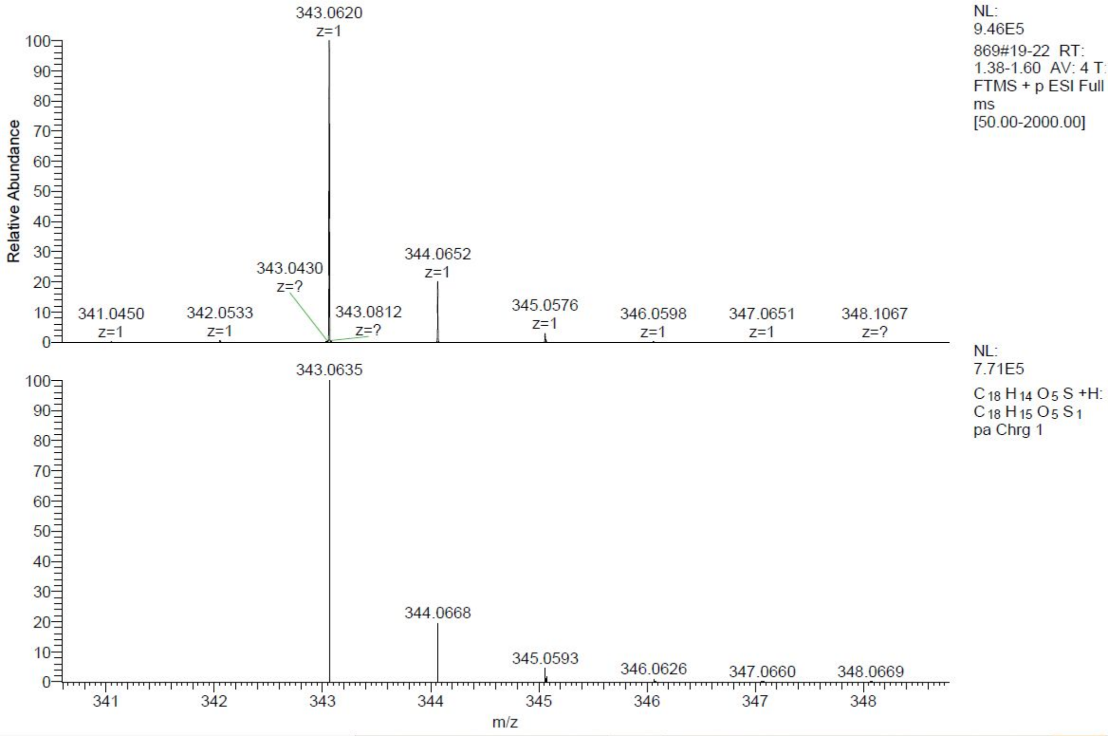

Fig. S21. HR-ESI-MS (+) spectrum of **11.** Upper: found MS; Lower: calculated MS.

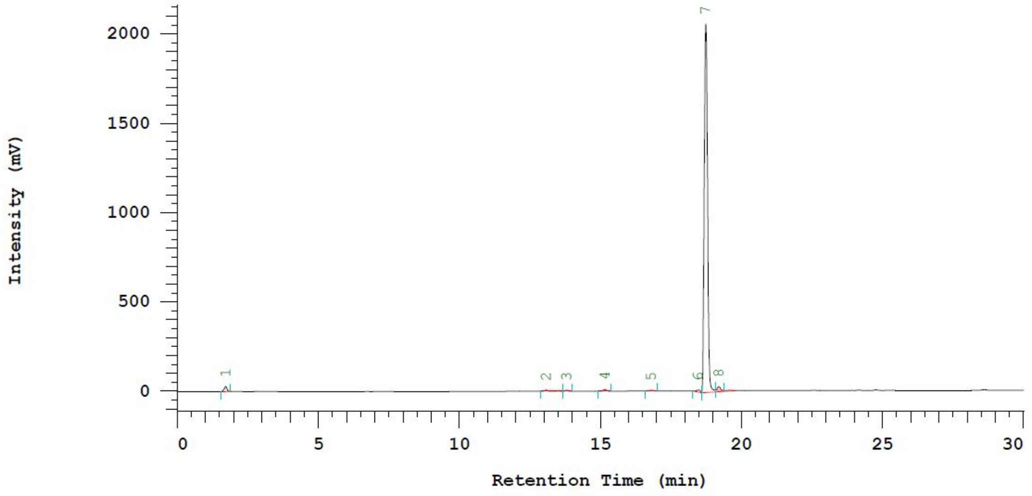

No.	RT	Area	Conc 1	BC
1	1.73	196215	1.112	BB
2	13.08	46375	0.263	BB
3	13.80	26437	0.150	BB
4	15.16	61212	0.347	BB
5	16.80	23654	0.134	BB
6	18.47	124363	0.705	BB
7	18.73	17012720	96.454	BV
8	19.20	147144	0.834	TBB
		17638120	100.000	

Fig. S22. . HPLC analysis of **11**.

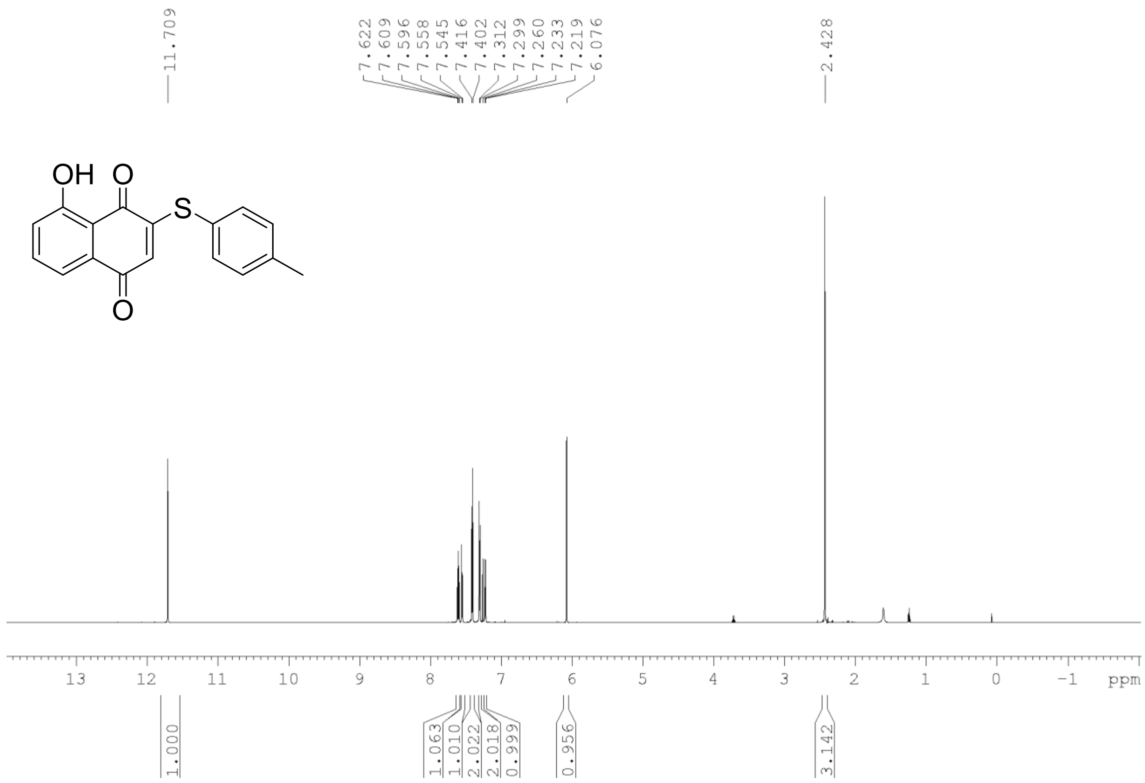

Fig. S23. ^{1}H NMR spectrum of **12** ($CDCl_3$, 600 MHz).

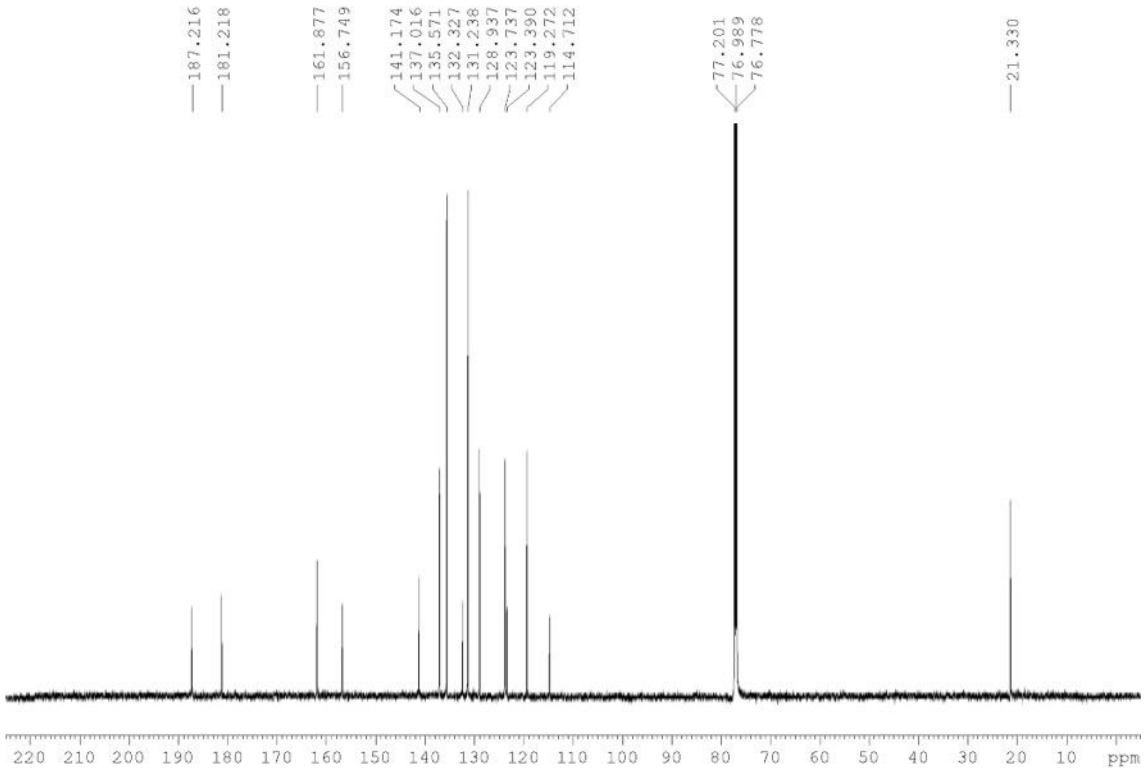

Fig. S24. ^{13}C NMR spectrum of **12** ($CDCl_3$, 150 MHz).

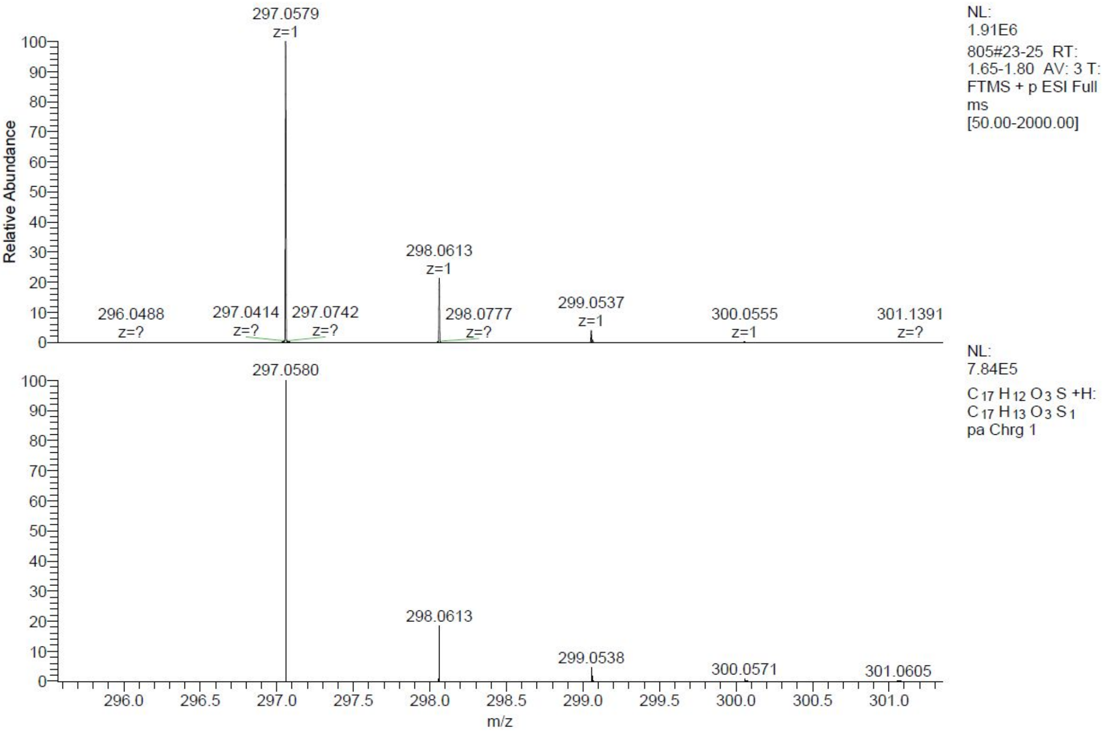

Fig. S25. HR-ESI-MS (+) spectrum of **12.** Upper: found MS; Lower: calculated MS.

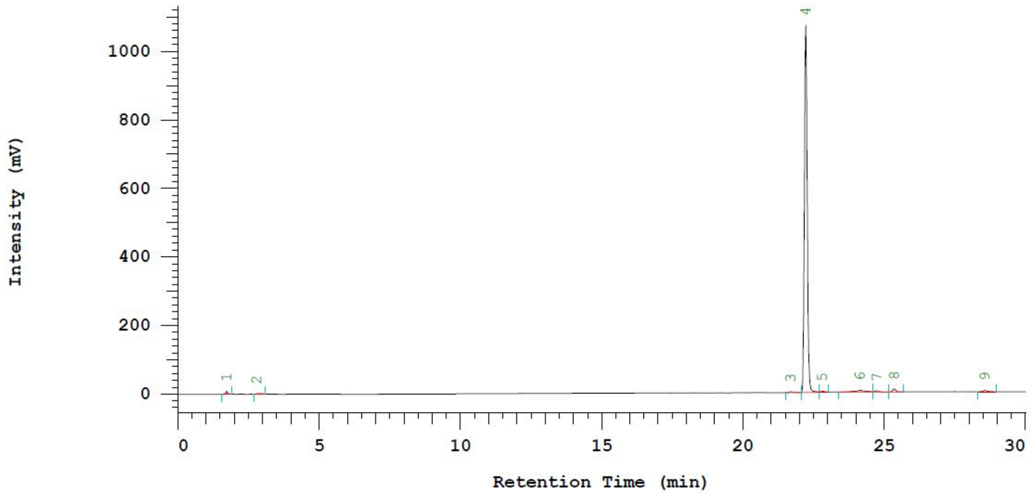

No.	RT	Area	Conc 1	BC
1	1.73	38741	0.492	BB
2	2.79	16761	0.213	BB
3	21.69	27192	0.345	BB
4	22.23	7495814	95.157	BB
5	22.81	17508	0.222	TBB
6	24.15	136448	1.732	BB
7	24.75	33139	0.421	BB
8	25.36	63766	0.809	BB
9	28.56	47957	0.609	BB
		7877326	100.000	

Fig. S26. HPLC analysis of **12**.

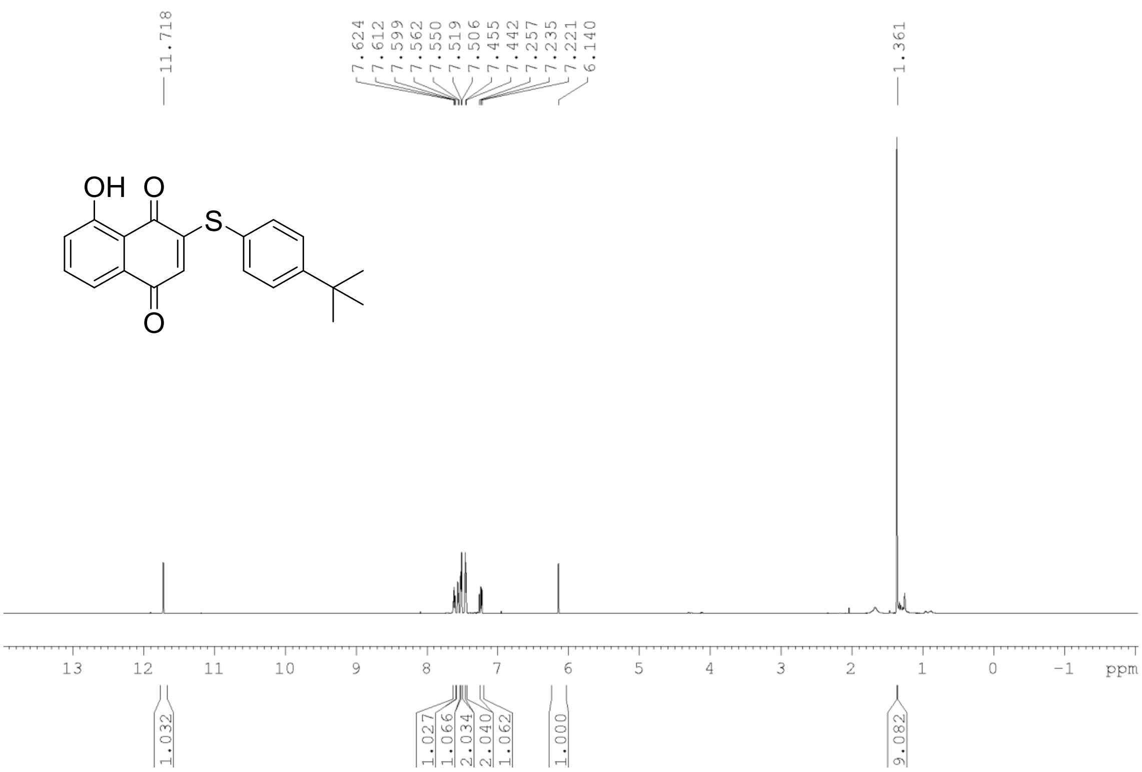

Fig. S27. ^{1}H NMR spectrum of **13** ($CDCl_3$, 400 MHz).

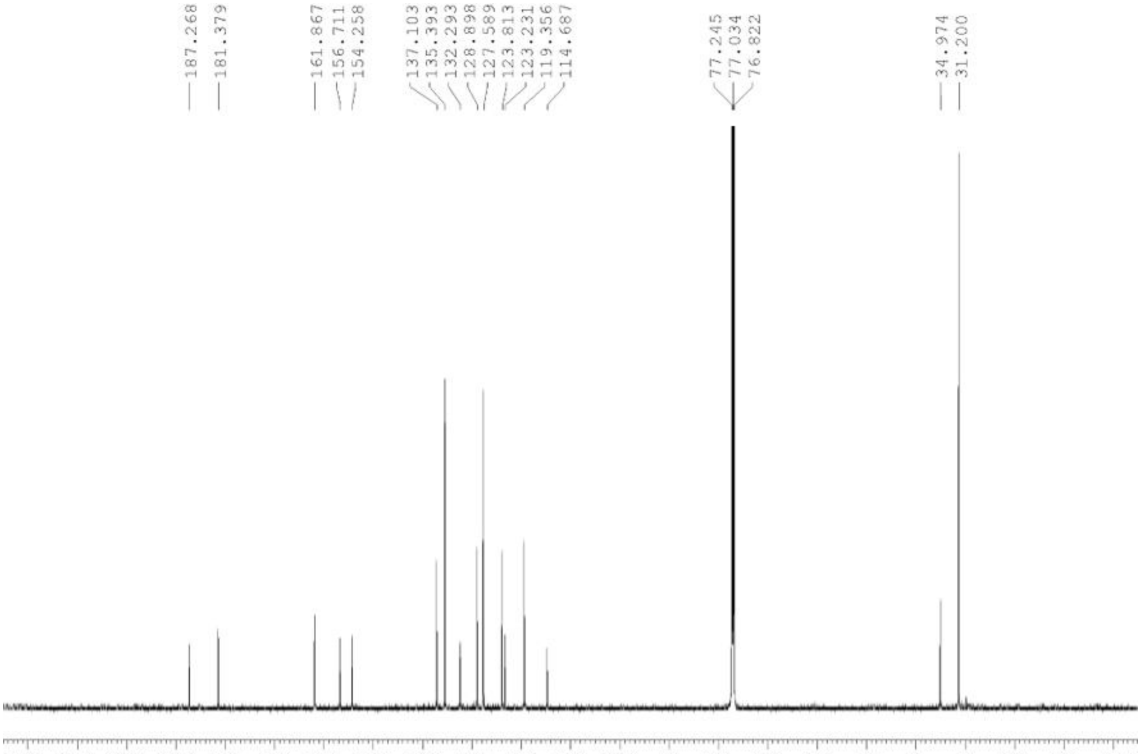

Fig. S28. ^{13}C NMR spectrum of **13** ($CDCl_3$, 100 MHz).

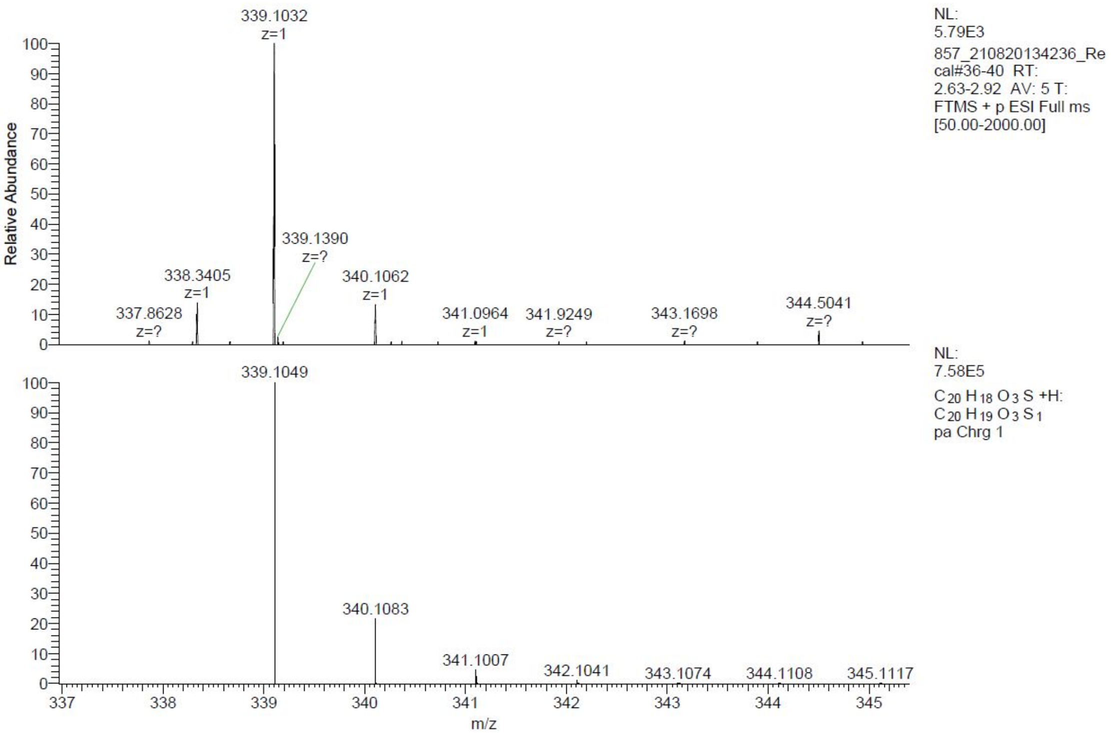

Fig. S29. HR-ESI-MS (+) spectrum of **13.** Upper: found MS; Lower: calculated MS.

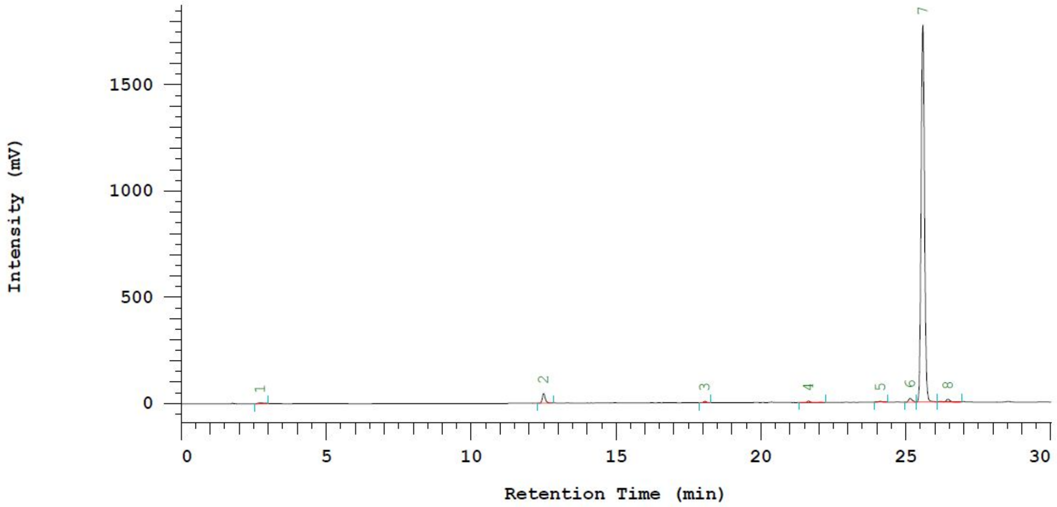

No.	RT	Area	Conc 1	BC
1	2.74	36434	0.238	BB
2	12.51	321015	2.095	BB
3	18.07	44990	0.294	BB
4	21.64	70954	0.463	BB
5	24.12	30845	0.201	BB
6	25.15	150775	0.984	BB
7	25.59	14585953	95.173	BB
8	26.45	84756	0.553	TBB
		15325722	100.000	

Fig. S30. HPLC analysis of **13**.

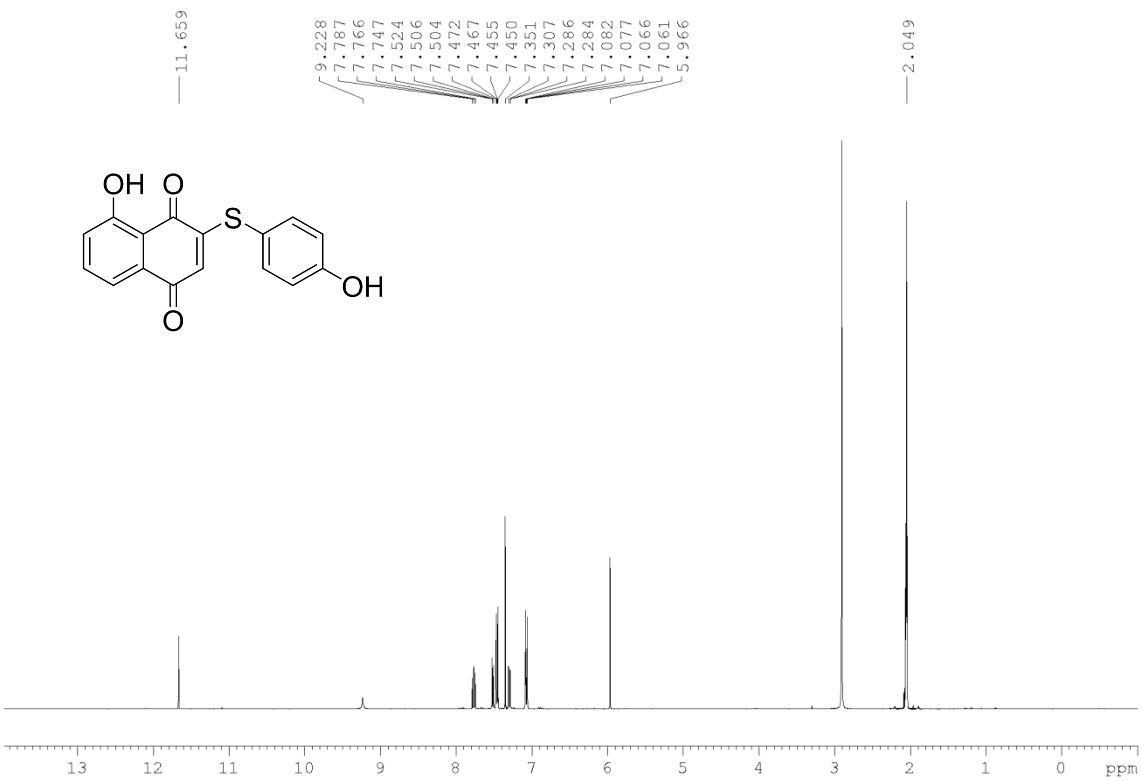

Fig. S31. ^{1}H NMR spectrum of **14** ($(CD_3)_2CO$, 400 MHz).

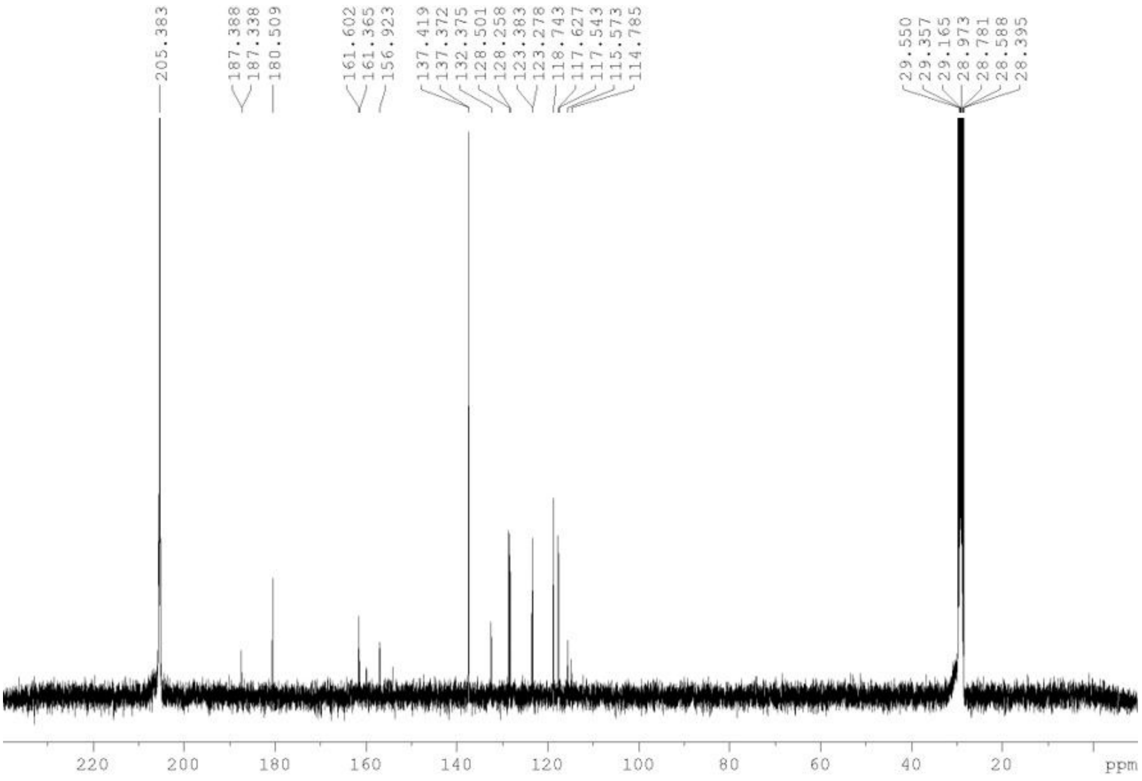

Fig. S32. ^{13}C NMR spectrum of **14** ($(CD_3)_2CO$, 100 MHz).

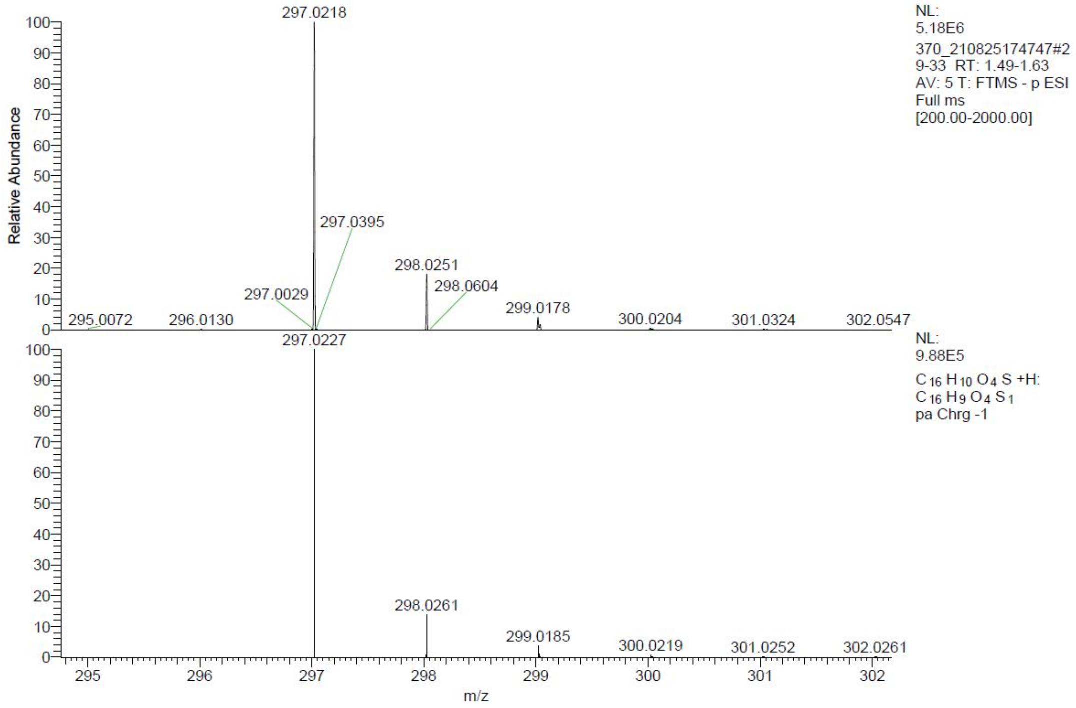

Fig. S33. HR-ESI-MS (+) spectrum of **14.** Upper: found MS; Lower: calculated MS.

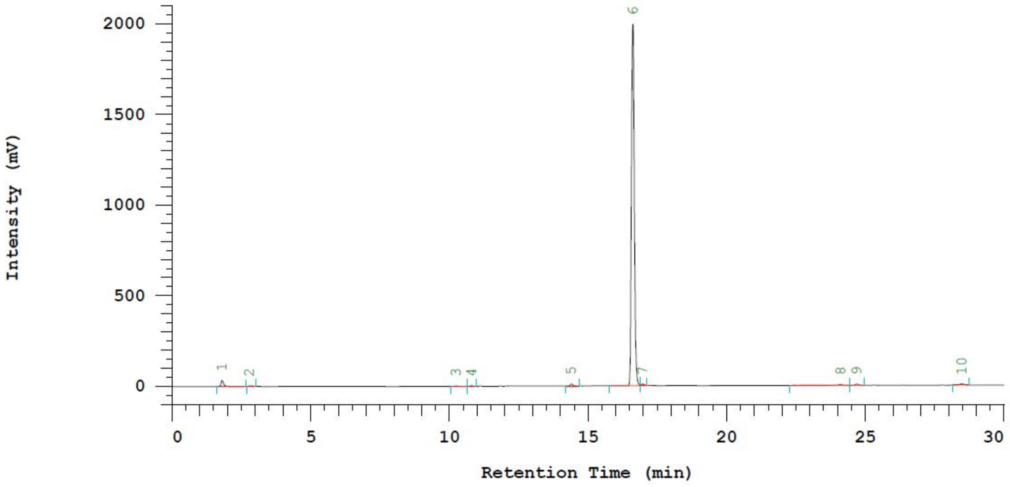

No.	RT	Area	Conc 1	BC
1	1.81	236347	1.489	BB
2	2.79	25647	0.162	BB
3	10.25	9260	0.058	BB
4	10.80	17073	0.108	BB
5	14.40	69514	0.438	BB
6	16.63	15264340	96.168	BV
7	16.97	34059	0.215	TBB
8	24.11	87274	0.550	BB
9	24.69	53400	0.336	BB
10	28.48	75676	0.477	BB
		15872590	100.000	

Fig. S34. HPLC analysis of **14**.

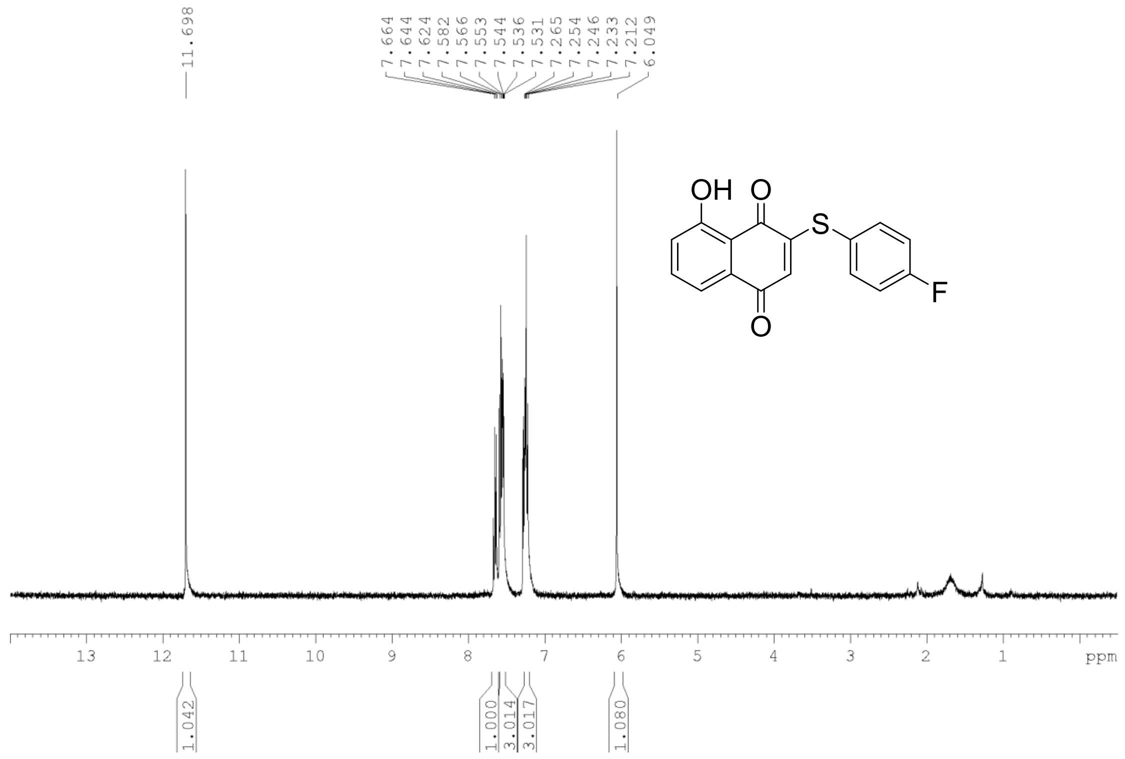

Fig. S35. ^{1}H NMR spectrum of **15** ($CDCl_3$, 400 MHz).

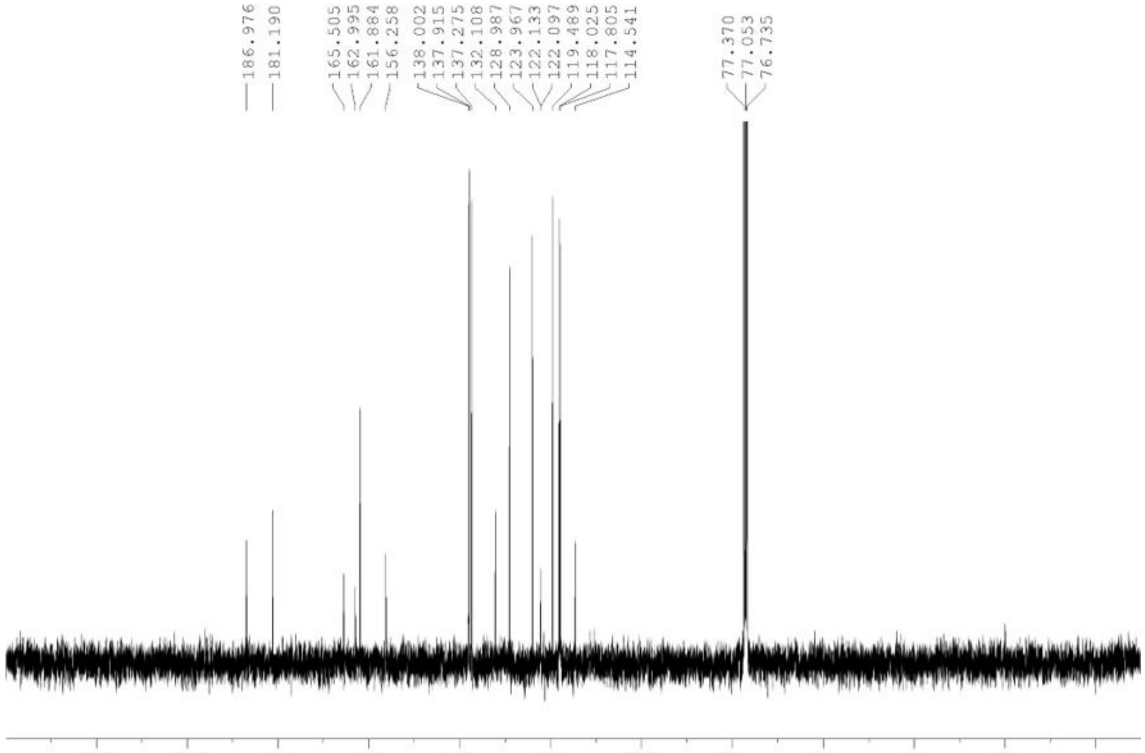

Fig. S36. ^{13}C NMR spectrum of **15** ($CDCl_3$, 100 MHz).

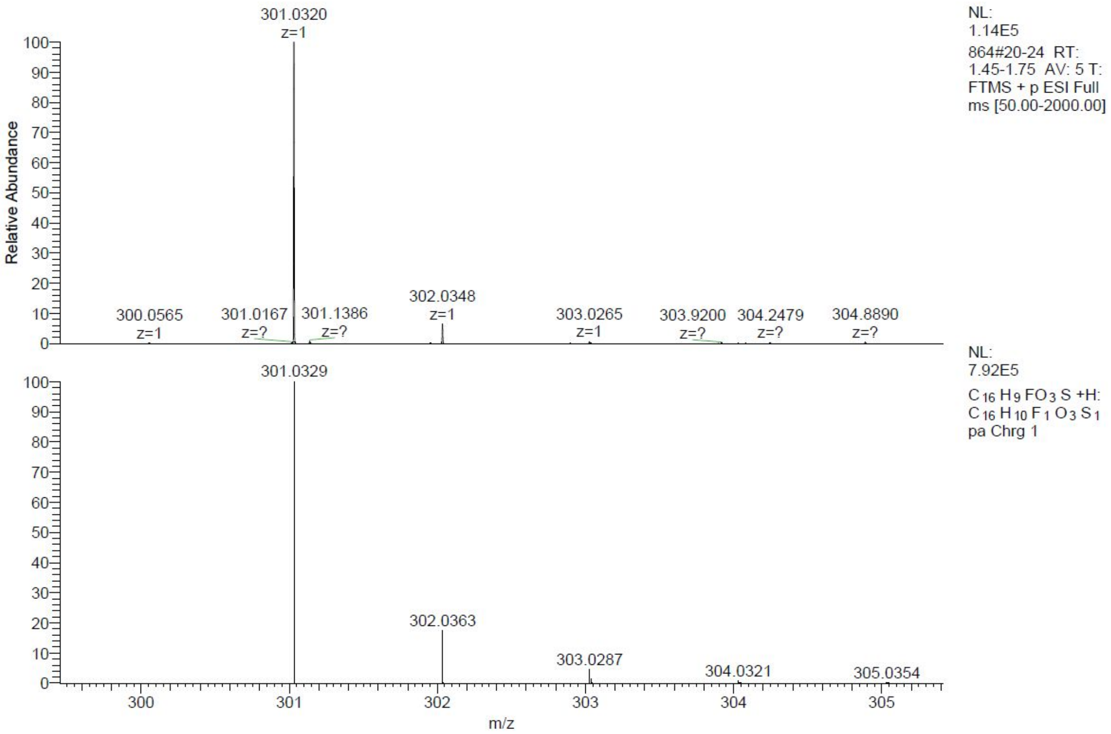

Fig. S37. HR-ESI-MS (+) spectrum of **15.** Upper: found MS; Lower: calculated MS.

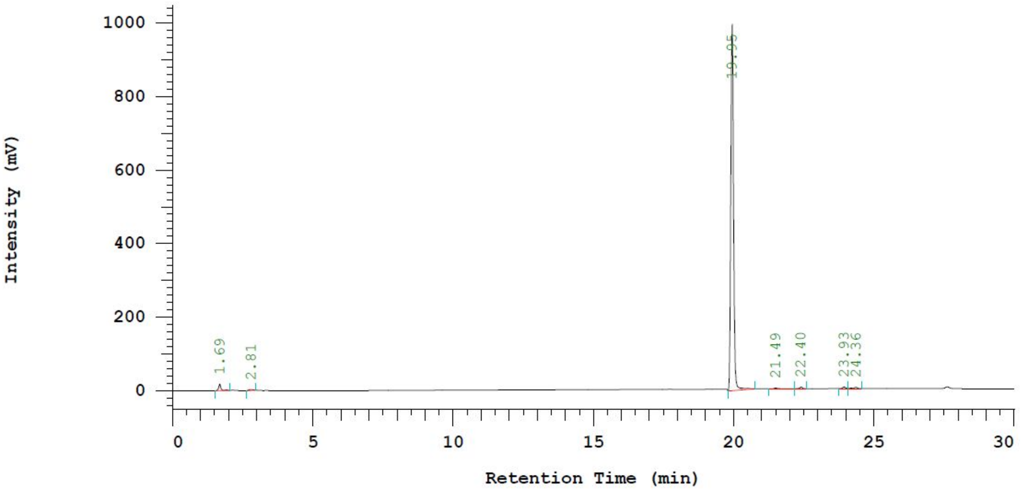

No.	RT	Area	Conc 1	BC
1	1.69	100205	1.372	BB
2	2.81	23442	0.321	BB
3	19.95	7043696	96.475	BB
4	21.49	25066	0.343	BB
5	22.40	37620	0.515	BB
6	23.93	32278	0.442	BB
7	24.36	38746	0.531	BB
		7301053	100.000	

Fig. S38. HPLC analysis of **15**.

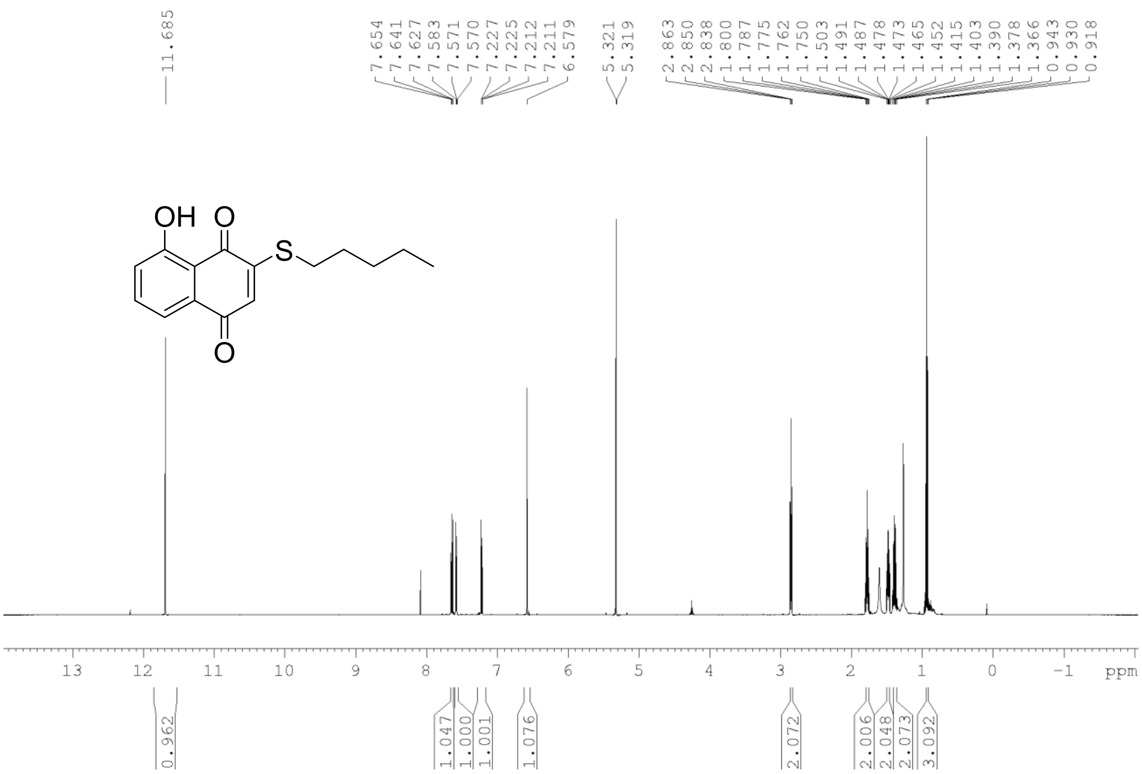

Fig. S39. ^{1}H NMR spectrum of **16** (CD_2Cl_2, 600 MHz).

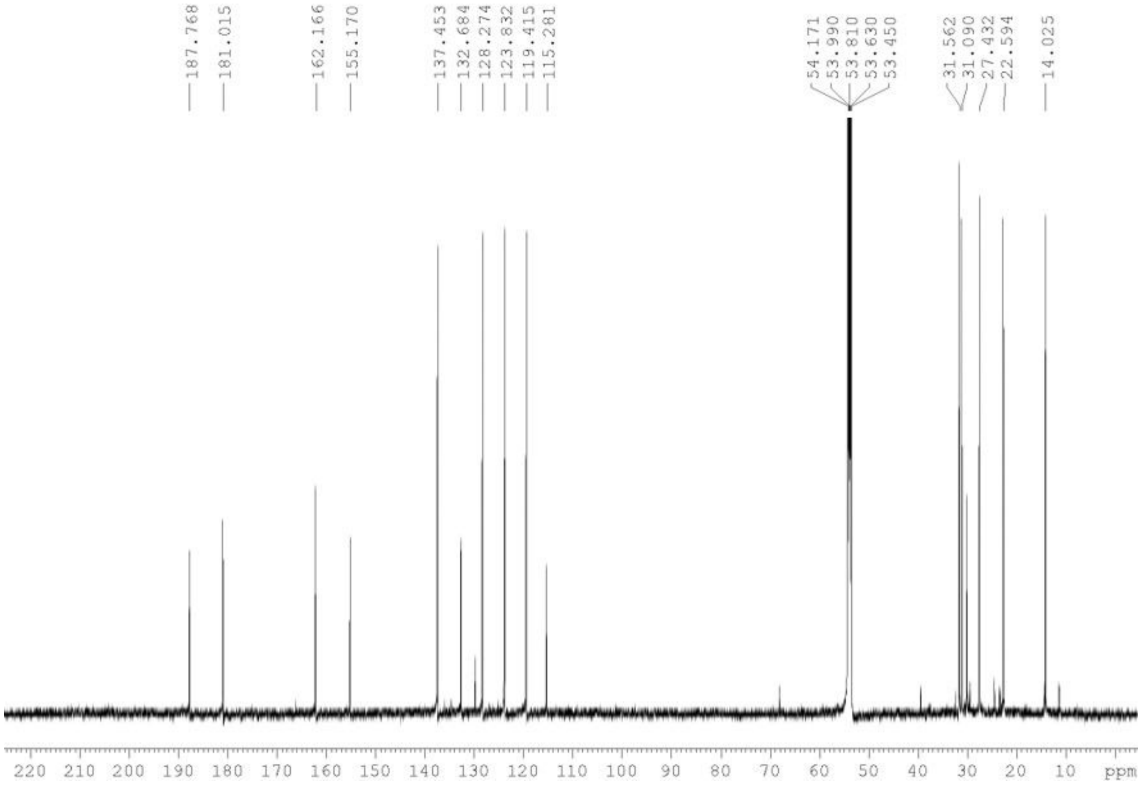

Fig. S40. ^{13}C NMR spectrum of **16** (CD_2Cl_2, 150 MHz).

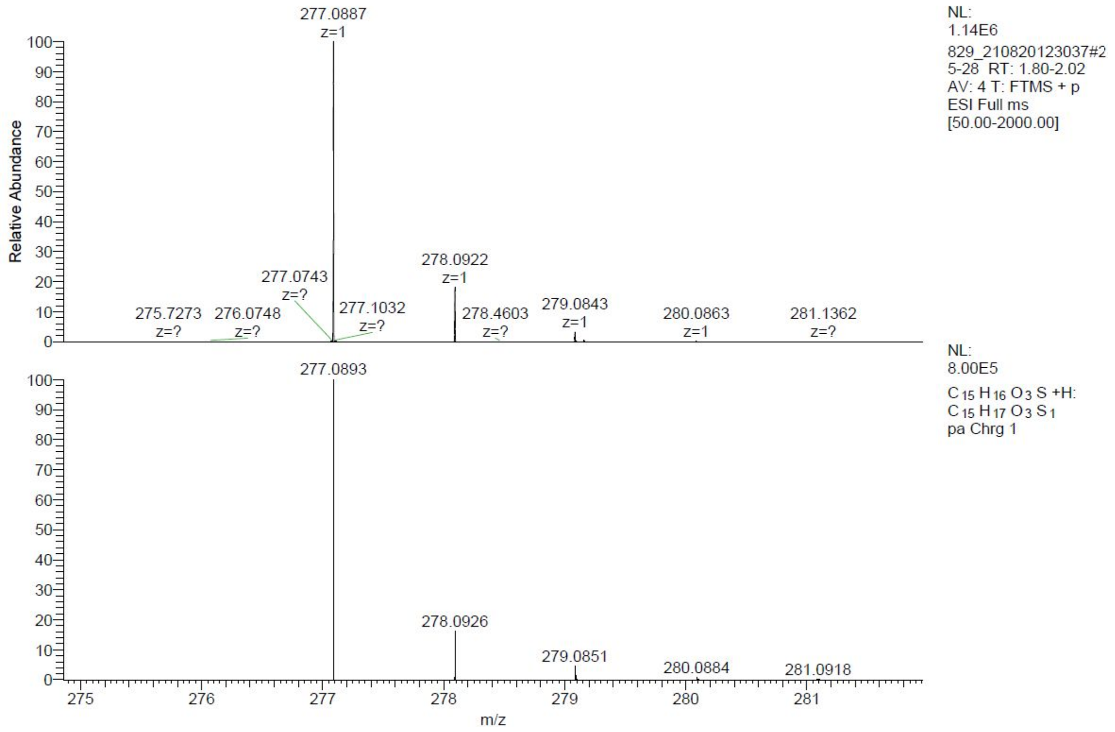

Fig. S41. HR-ESI-MS (+) spectrum of **16.** Upper: found MS; Lower: calculated MS.

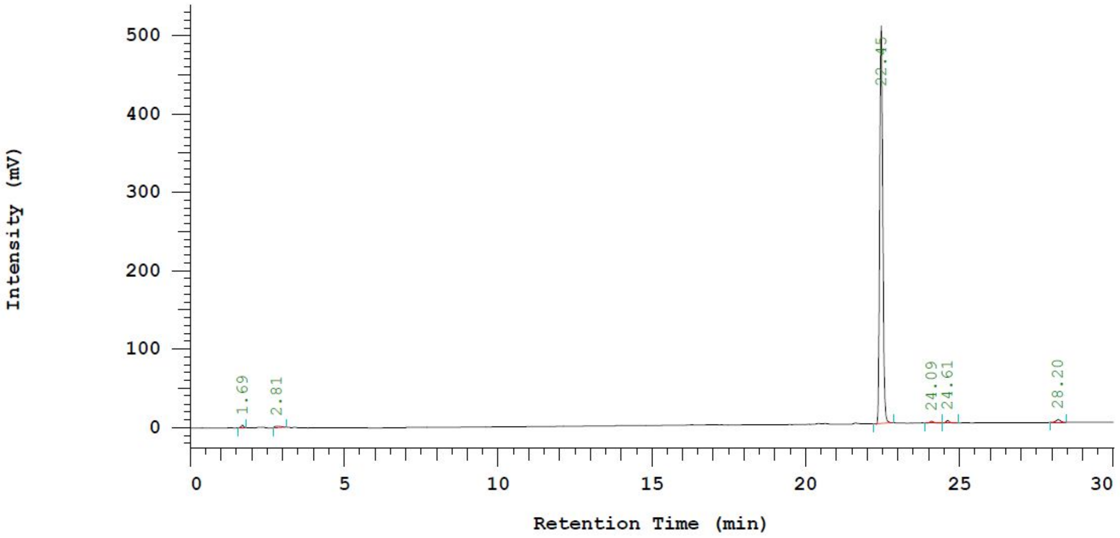

No.	RT	Area	Conc 1	BC
1	1.69	16865	0.467	BB
2	2.81	26401	0.731	BB
3	22.45	3491732	96.640	BB
4	24.09	18046	0.499	BB
5	24.61	22471	0.622	BB
6	28.20	37623	1.041	BB
		3613138	100.000	

Fig. S42. HPLC analysis of **16**.

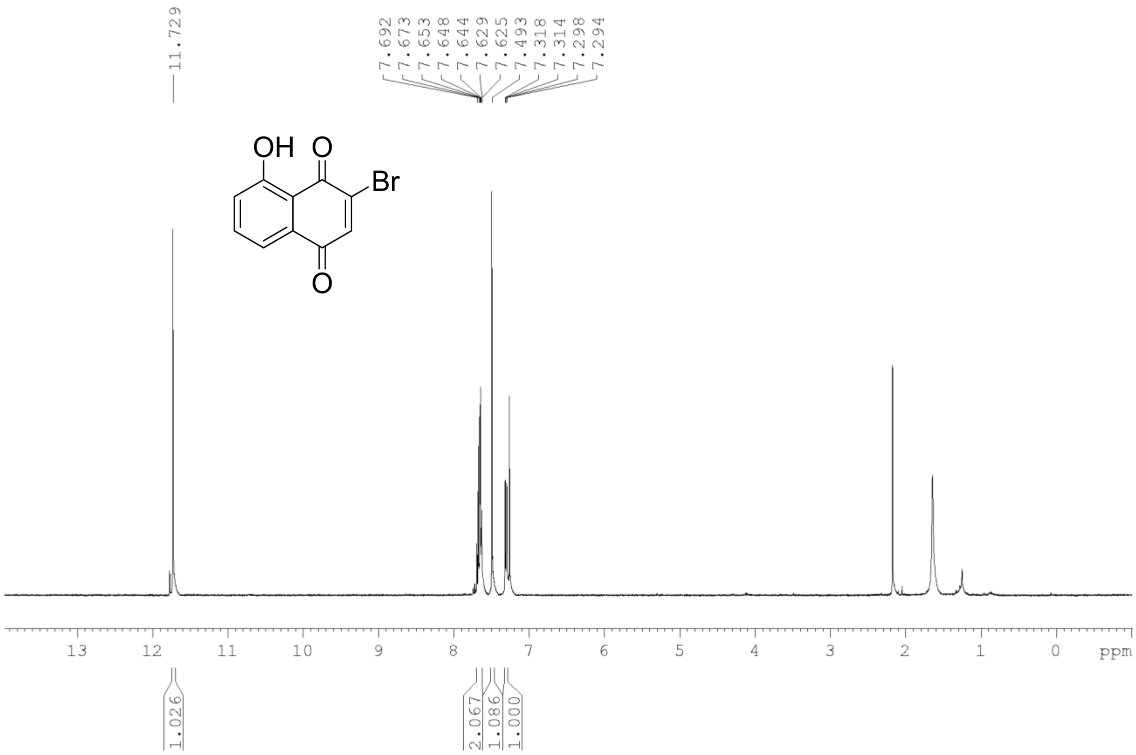

Fig. S43. ^{1}H NMR spectrum of **17** ($CDCl_3$, 400 MHz).

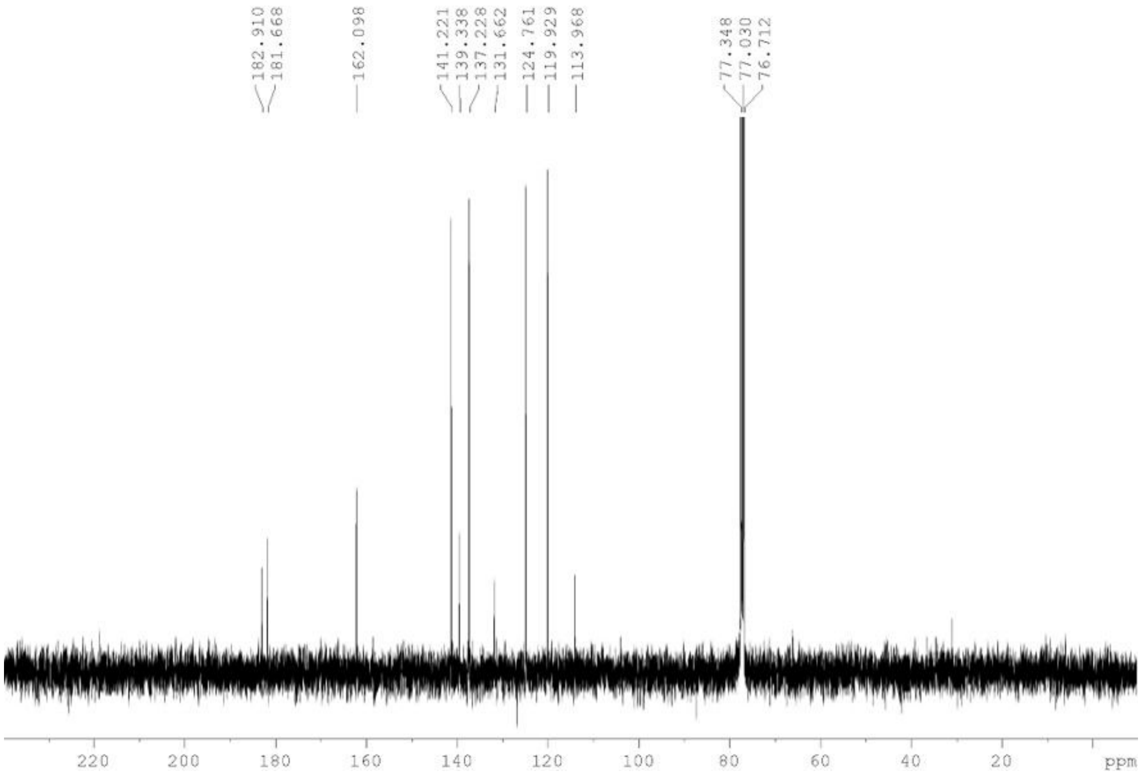

Fig. S44. ^{13}C NMR spectrum of **17** ($CDCl_3$, 100 MHz).

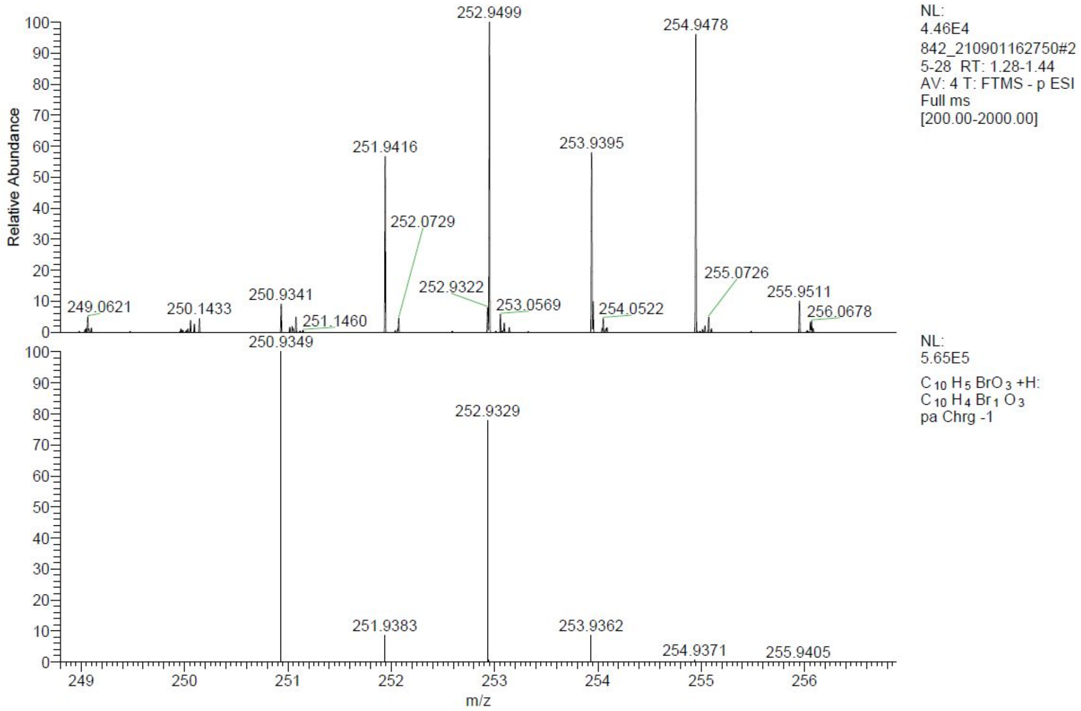

Fig. S45. HR-ESI-MS (+) spectrum of **17.** Upper: found MS; Lower: calculated MS.

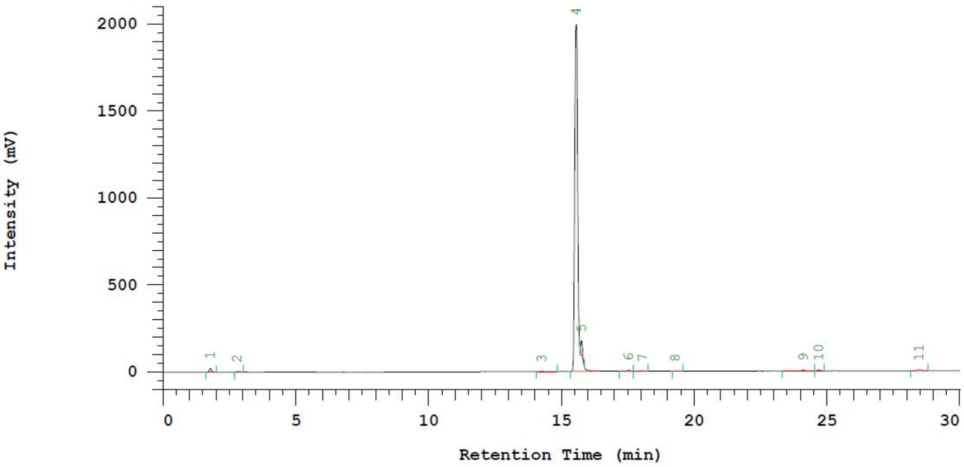

No.	RT	Area	Conc 1	BC
1	1.79	132796	0.774	BB
2	2.79	25405	0.148	BB
3	14.27	13984	0.082	BB
4	15.56	16333134	95.256	BV
5	15.76	416522	2.429	TBB
6	17.53	43969	0.256	BB
7	18.07	13099	0.076	BB
8	19.29	7440	0.043	BB
9	24.11	53405	0.311	BB
10	24.69	40828	0.238	BB
11	28.47	65907	0.384	BB
		17146489	100.000	

Fig. S46. HPLC analysis of **17**.

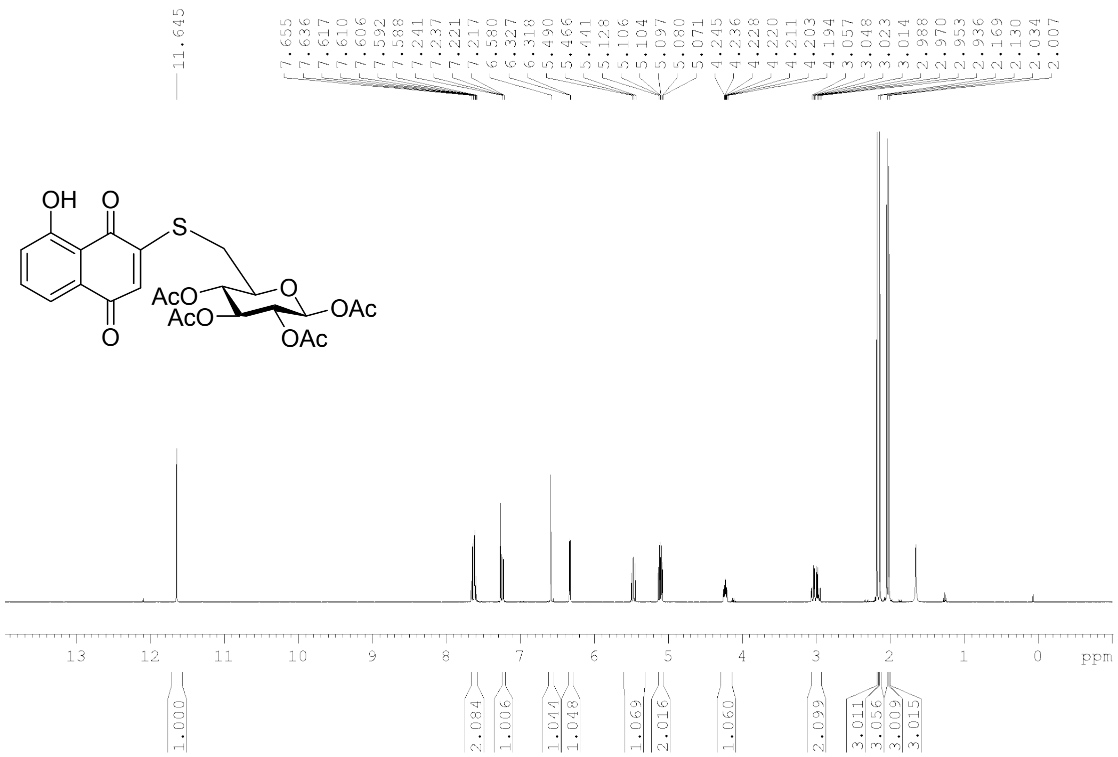

Fig. S47. ^{1}H NMR spectrum of **18** ($CDCl_3$, 400 MHz).

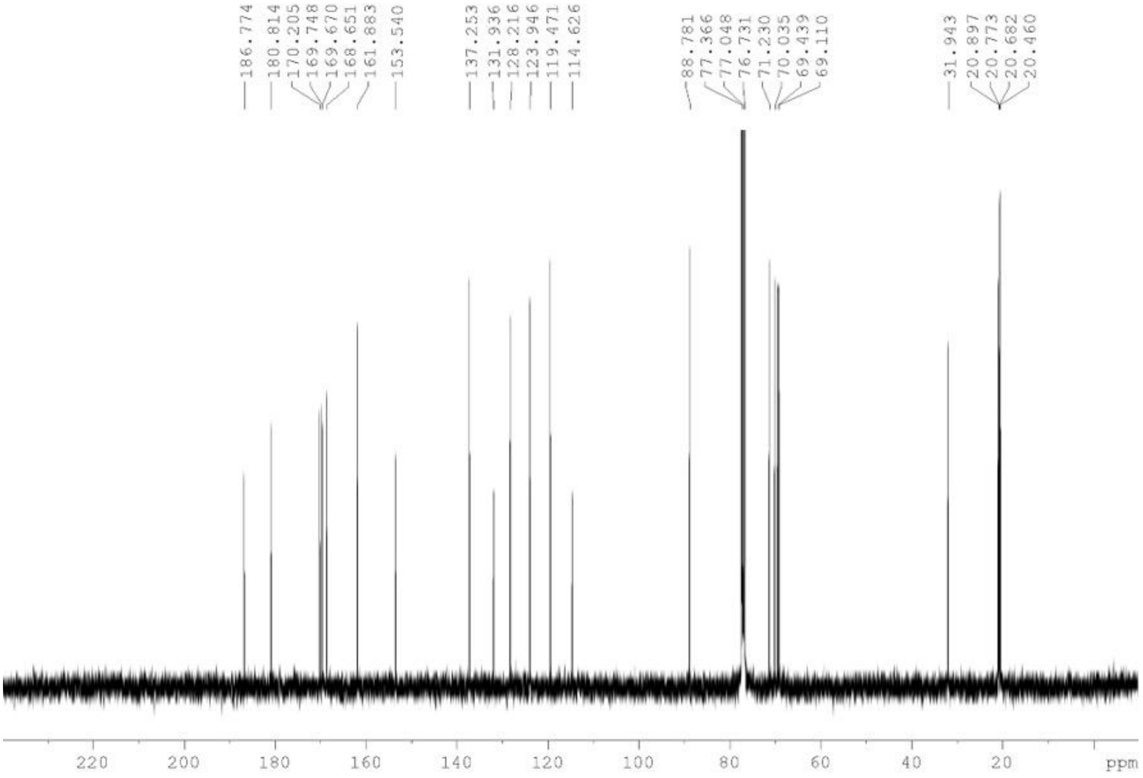

Fig. S48. ^{13}C NMR spectrum of **18** ($CDCl_3$, 100 MHz).

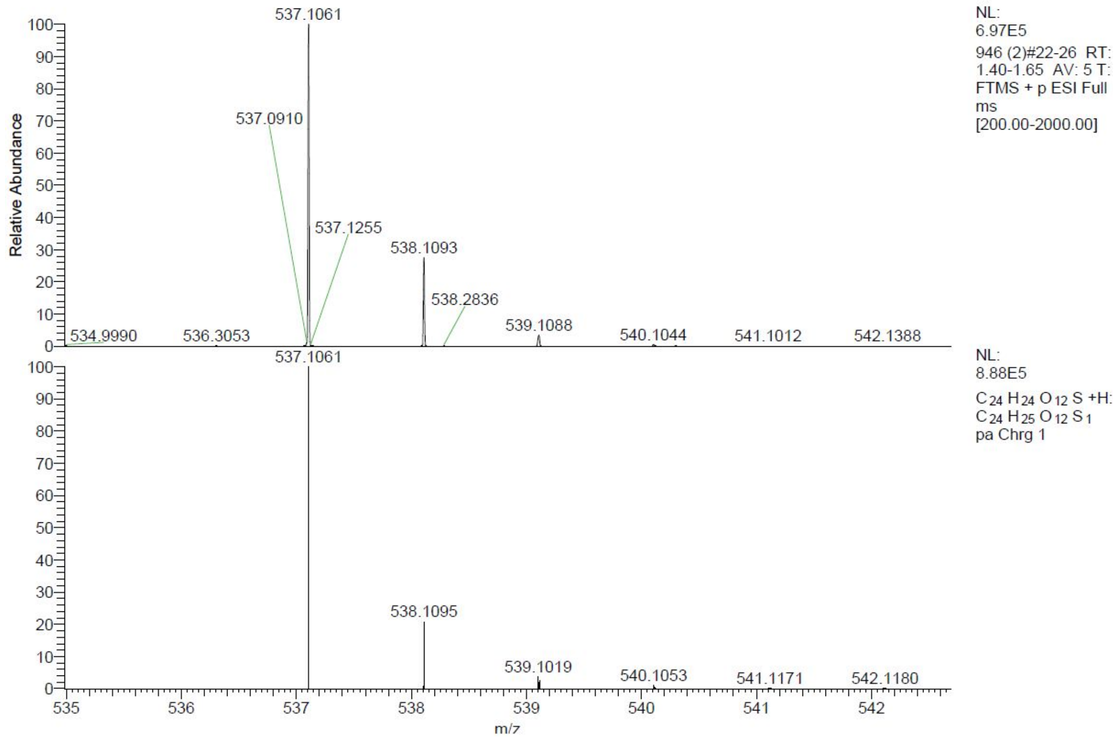

Fig. S49. HR-ESI-MS (+) spectrum of **18.** Upper: found MS; Lower: calculated MS.

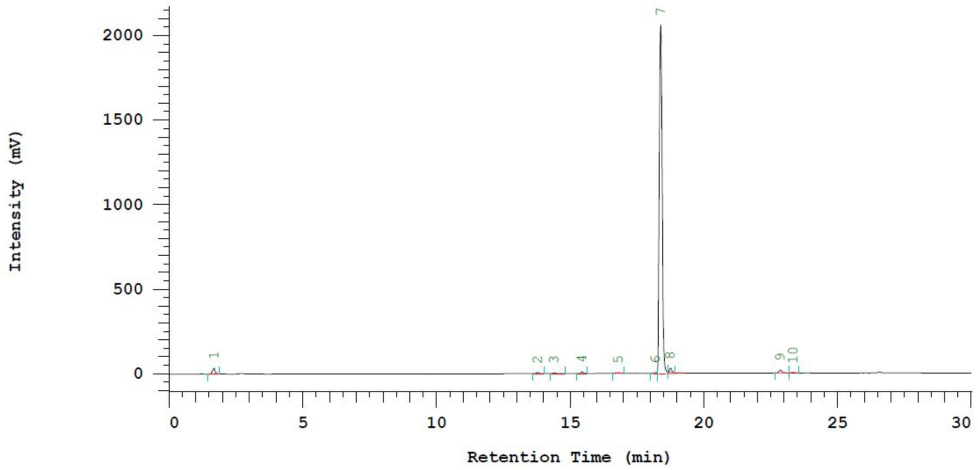

No.	RT	Area	Conc 1	BC
1	1.69	228430	1.357	BB
2	13.79	49735	0.295	BB
3	14.40	23903	0.142	BB
4	15.44	60399	0.359	BB
5	16.80	27993	0.166	BB
6	18.20	55768	0.331	BB
7	18.39	16060500	95.373	BV
8	18.75	156632	0.930	TBB
9	22.85	141803	0.842	BB
10	23.33	34444	0.205	BB
		16839607	100.000	

Fig. S50. HPLC analysis of **18**.

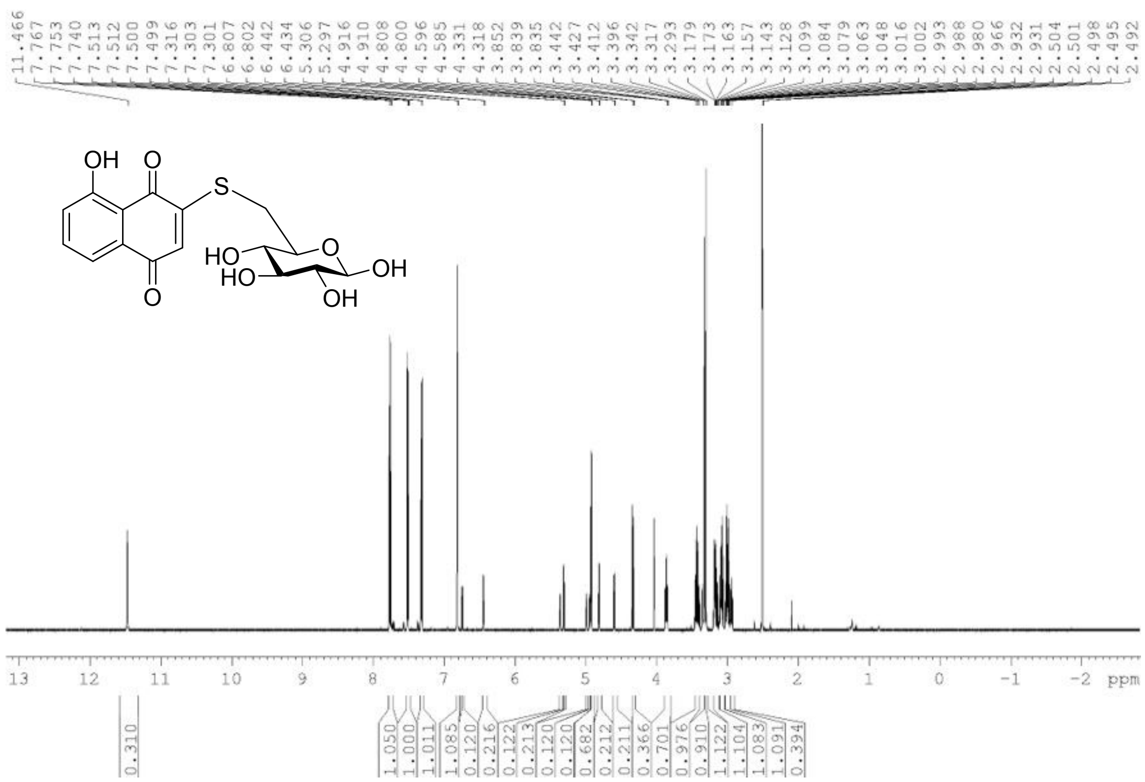

Fig. S51. ^{1}H NMR spectrum of **19** (α/β mixture) ($(CD_3)_2SO$, 400 MHz).

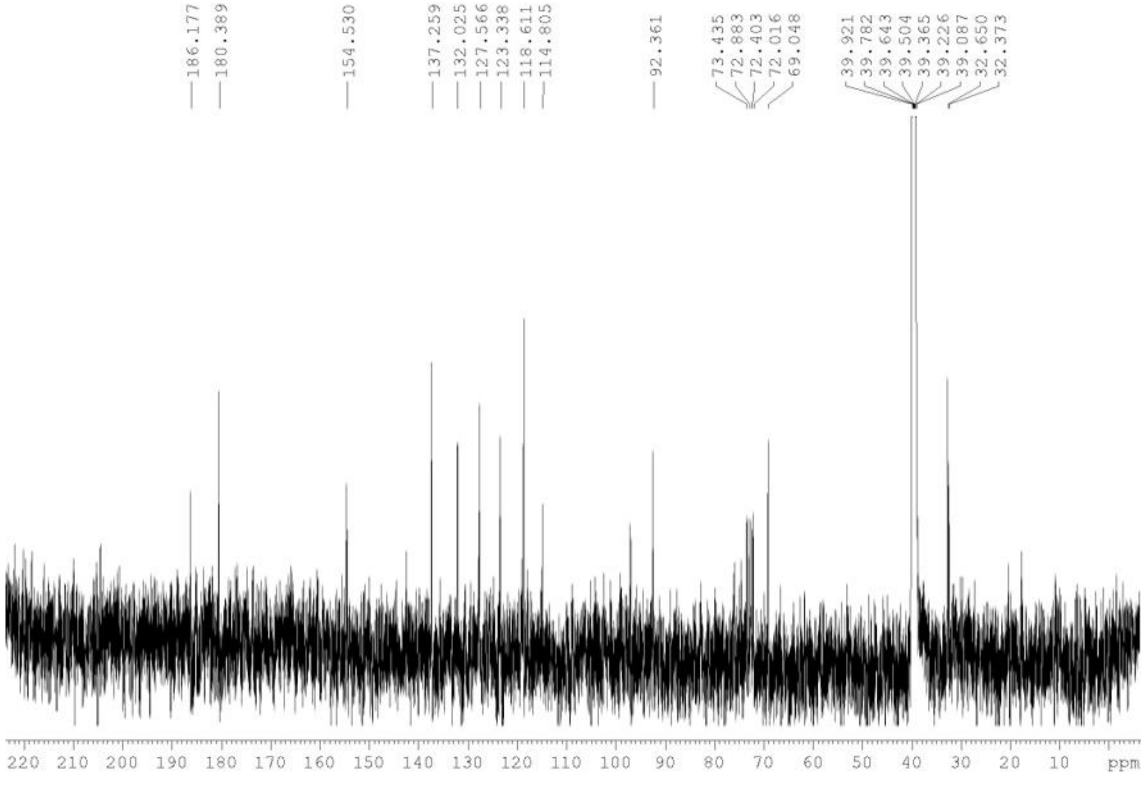

Fig. S52. ^{13}C NMR spectrum of **19** (α/β mixture) ($(CD_3)_2SO$, 100 MHz).

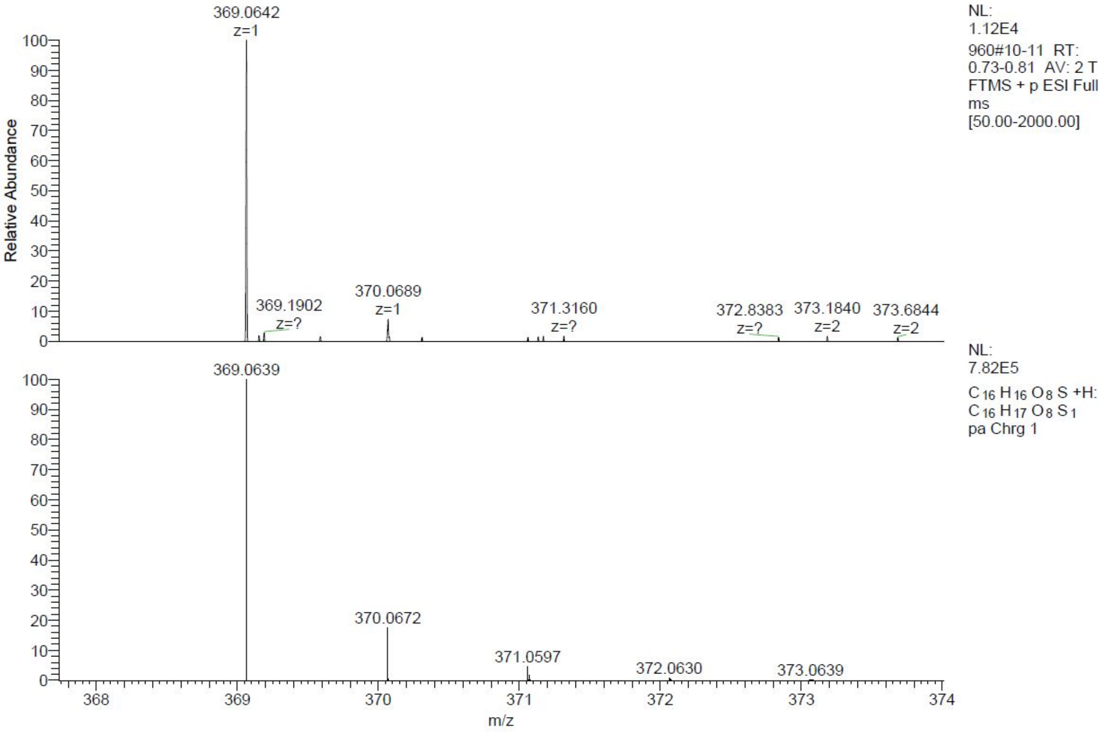

Fig. S53. HR-ESI-MS (+) spectrum of **19** (α/β mixture)**.** Upper: found MS; Lower: calculated MS.

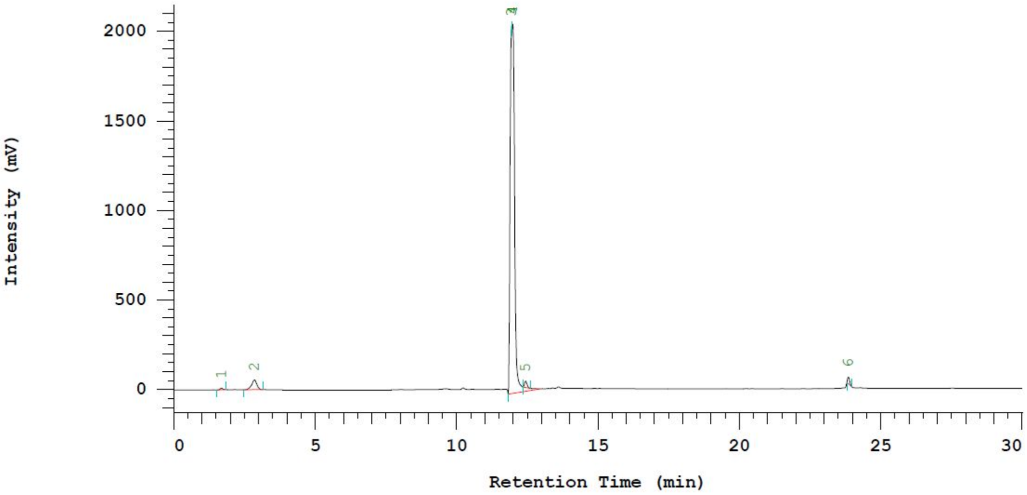

No.	RT	Area	Conc 1	BC
1	1.69	54764	0.228	BB
2	2.85	686621	2.864	BB
3	11.95	9918215	41.372	BV
4	11.99	12904084	53.827	VV
5	12.44	213538	0.891	TBB
6	23.85	195856	0.817	BB
		23973078	100.000	

Fig. S54. HPLC analysis of **19** (α/β mixture).

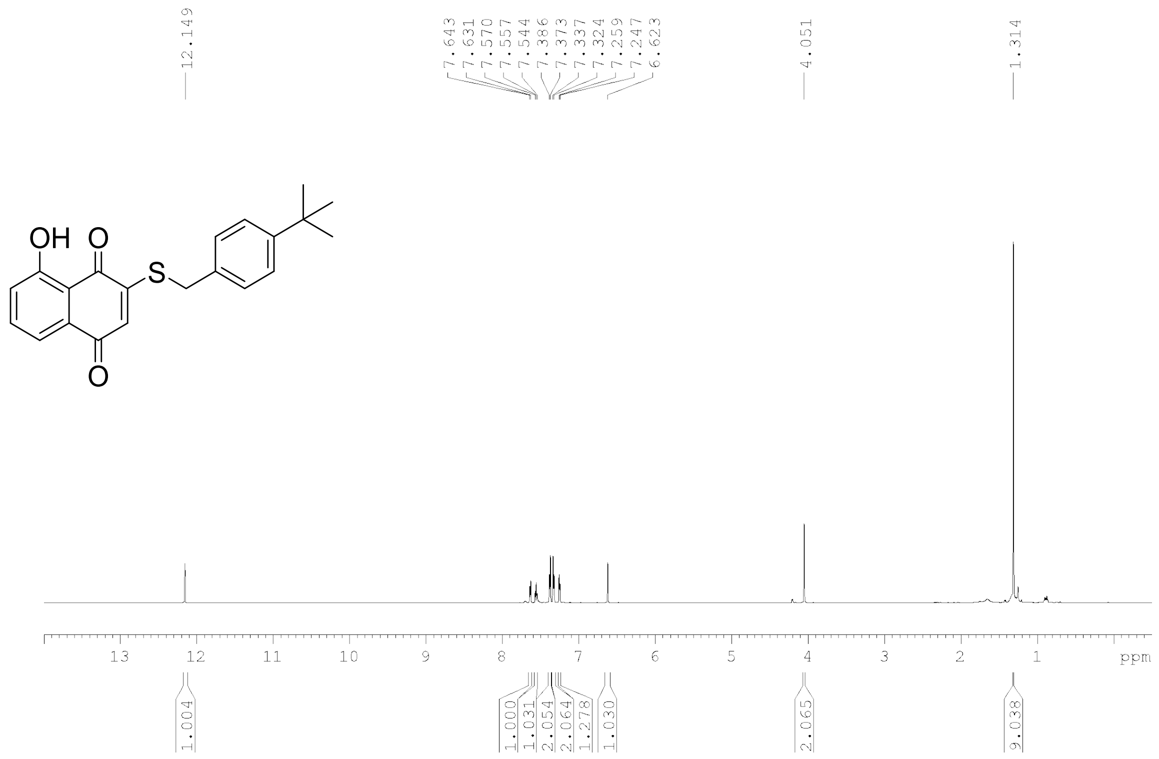

Fig. S55. ^{1}H NMR spectrum of **20** ($CDCl_3$, 400 MHz).

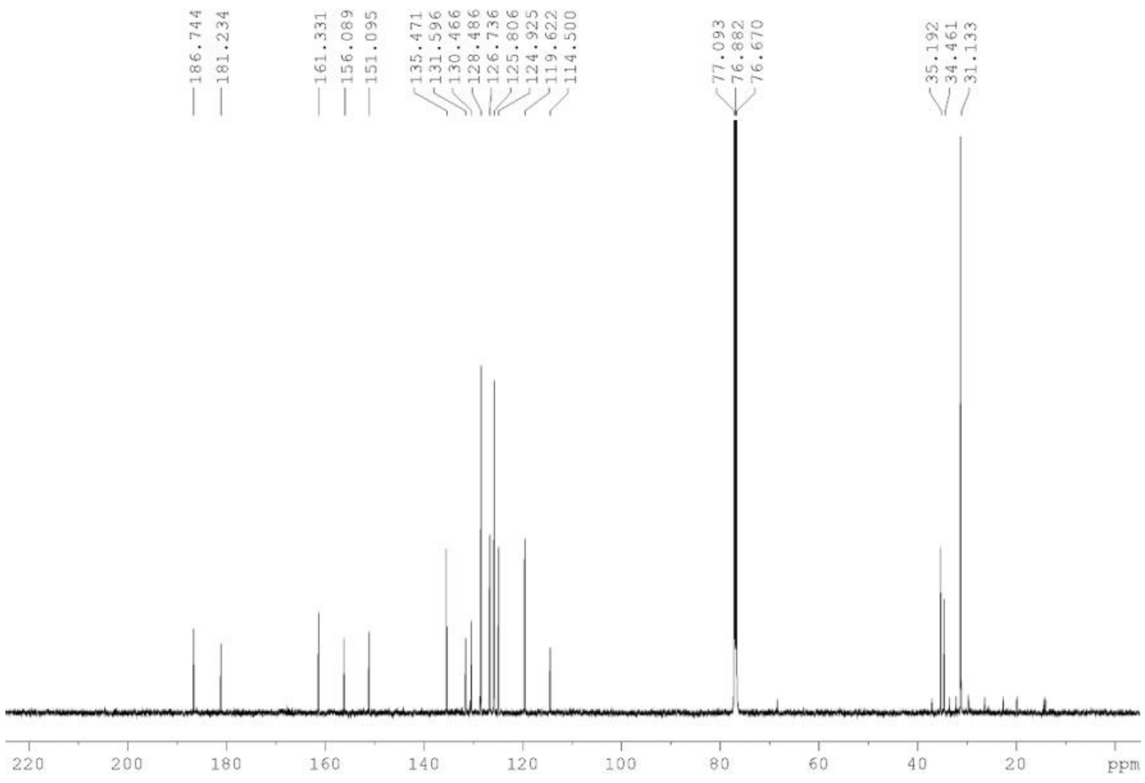

Fig. S56. ^{13}C NMR spectrum of **20** ($CDCl_3$, 100 MHz).

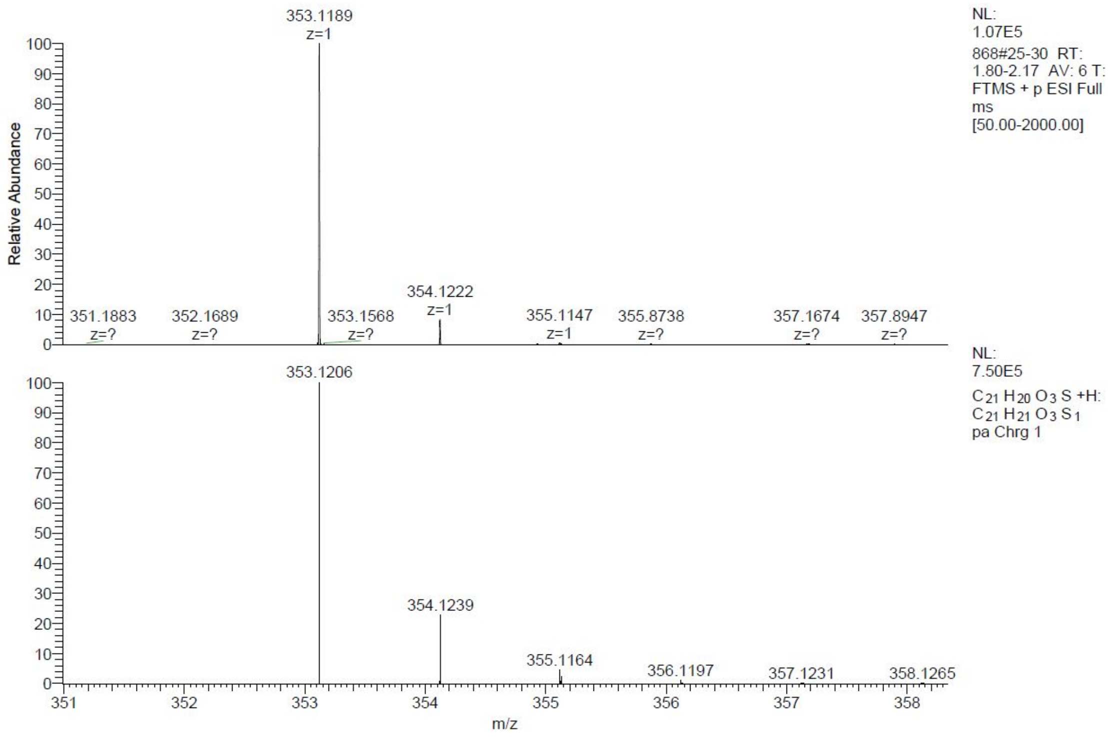

Fig. S57. HR-ESI-MS (-) spectrum of **20.** Upper: found MS; Lower: calculated MS.

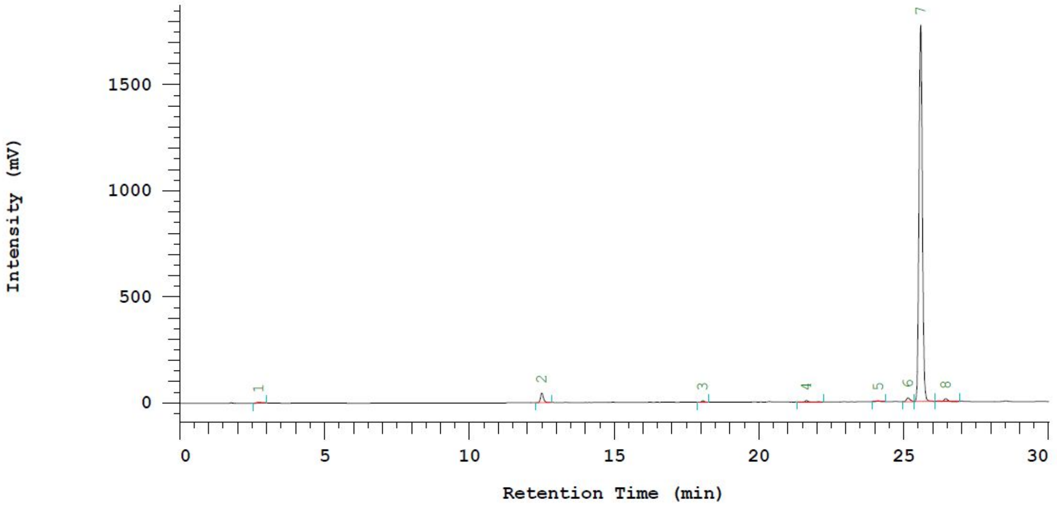

No.	RT	Area	Conc 1	BC
1	2.74	36434	0.238	BB
2	12.51	321015	2.095	BB
3	18.07	44990	0.294	BB
4	21.64	70954	0.463	BB
5	24.12	30845	0.201	BB
6	25.15	150775	0.984	BB
7	25.59	14585953	95.173	BB
8	26.45	84756	0.553	TBB
		15325722	100.000	

Fig. S58. HPLC analysis of **20**.

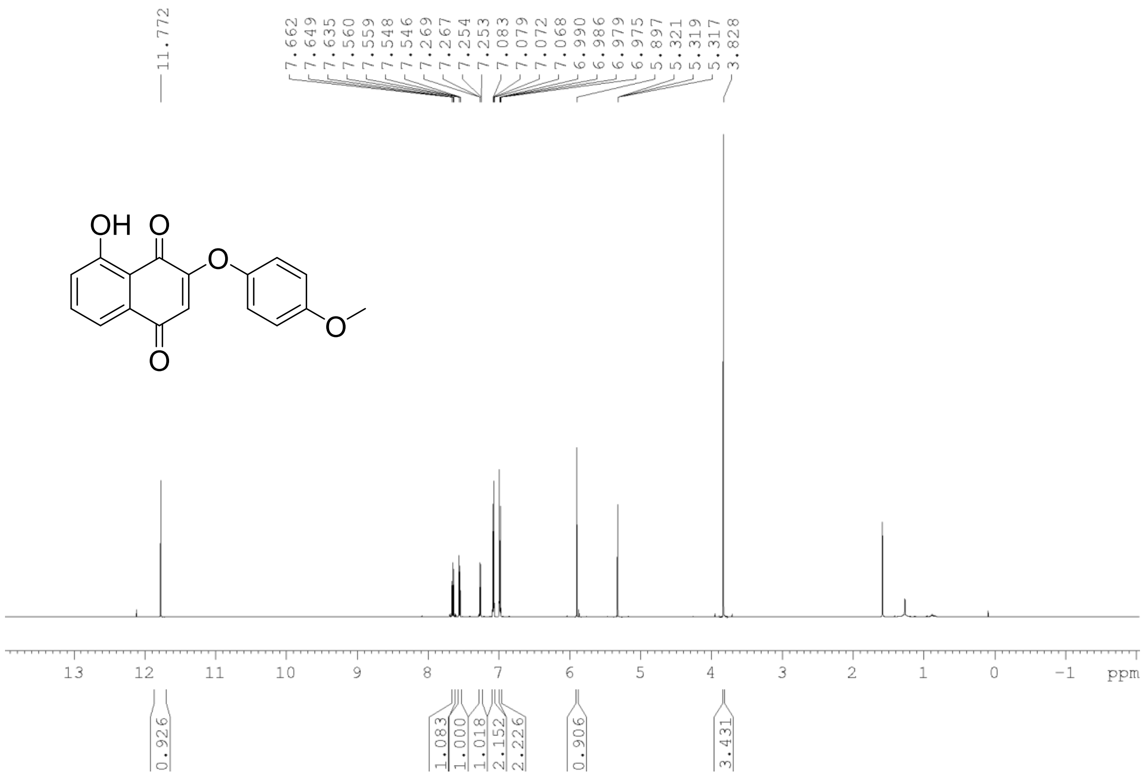

Fig. S59. ^{1}H NMR spectrum of **21** (CD_2Cl_2, 600 MHz).

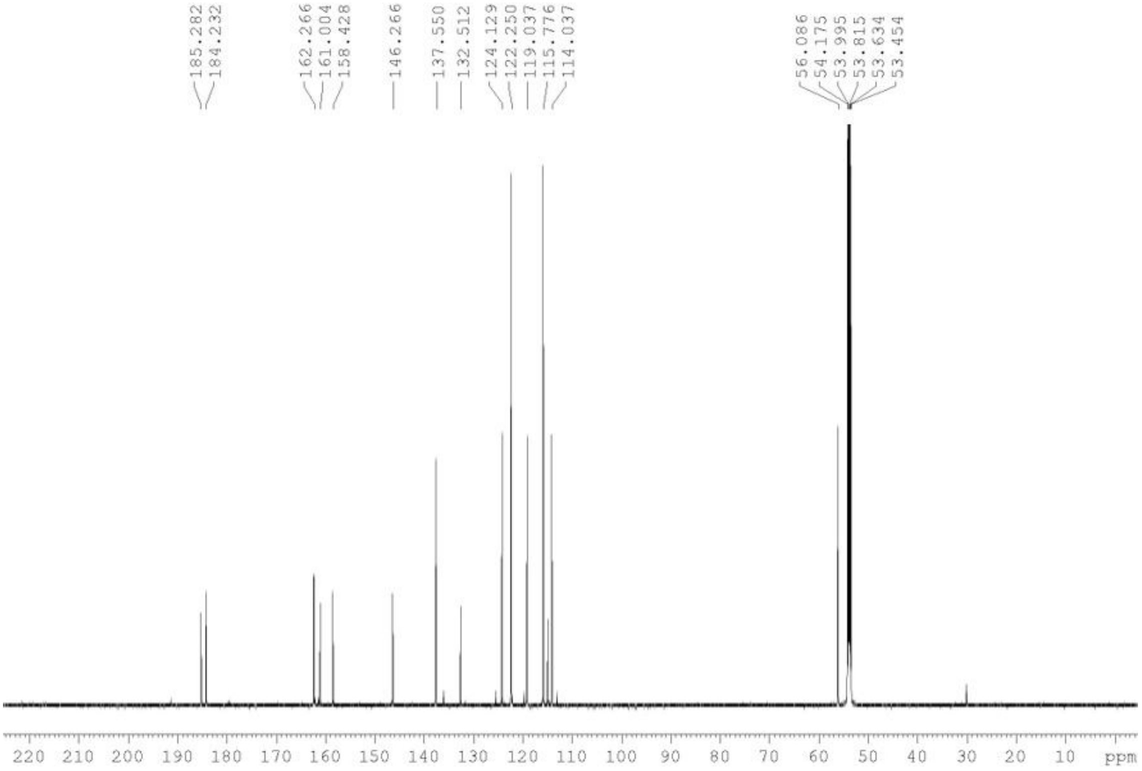

Fig. S60. ^{13}C NMR spectrum of **21** (CD_2Cl_2, 150 MHz).

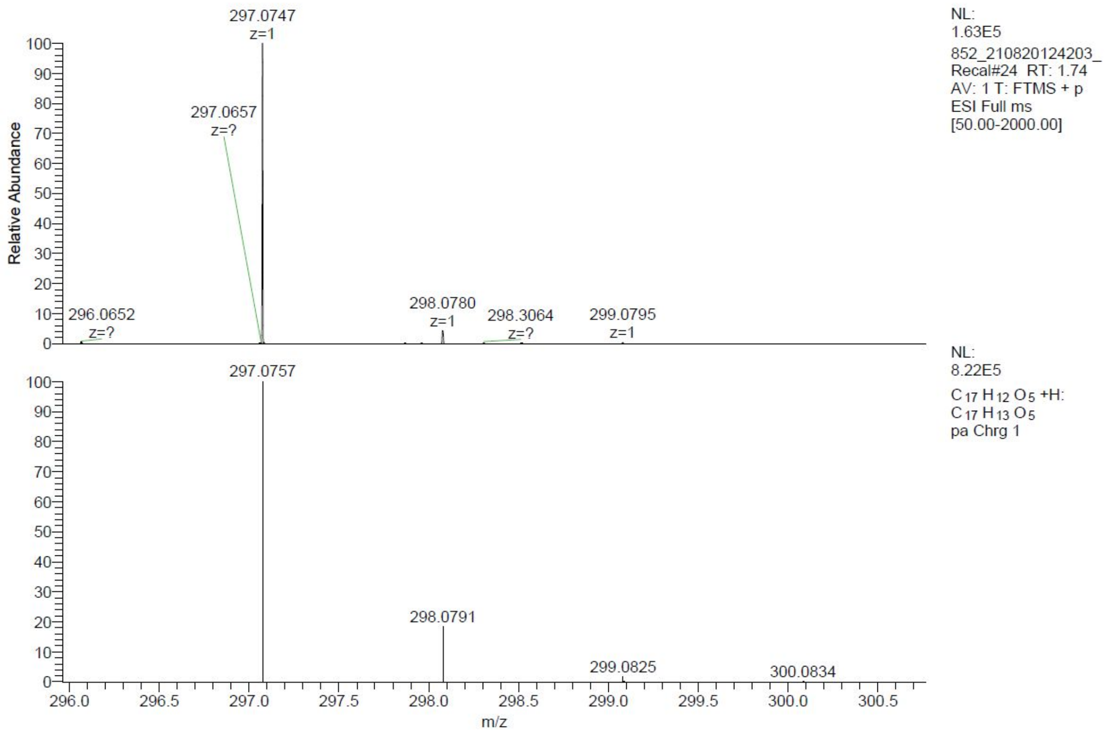

Fig. S61. HR-ESI-MS (+) spectrum of **21.** Upper: found MS; Lower: calculated MS.

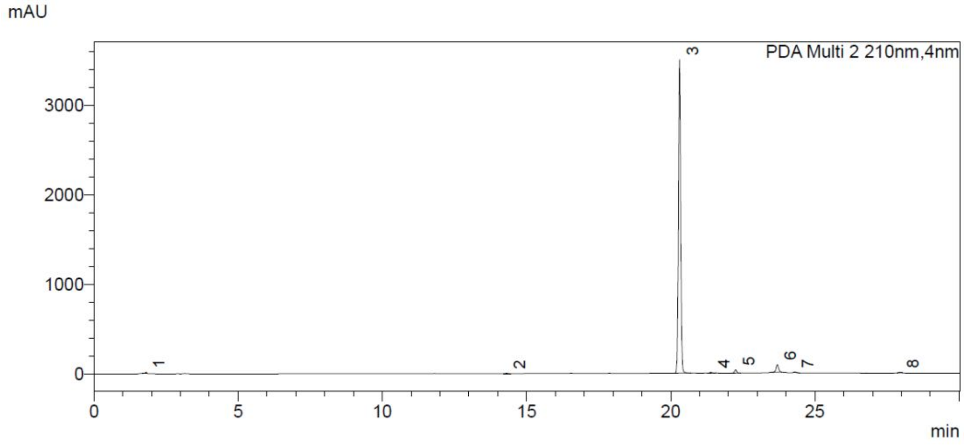

<Peak Table>

PDA Ch2 210nm

Peak#	Ret. Time	Area	Height	Conc.	Mark	Area%
1	1.806	56710	14388	0.000	M	0.290
2	14.310	26245	4275	0.000	M	0.134
3	20.308	18665310	3506474	0.000	M	95.390
4	21.393	31300	8264	0.000	M	0.160
5	22.251	191953	36085	0.000	M	0.981
6	23.697	558254	84597	0.000	M	2.853
7	24.303	726	2565	0.000	M	0.004
8	27.951	36772	5318	0.000	M	0.188
Total		19567271	3661966			100.000

Fig. S62. HPLC analysis of **21**.

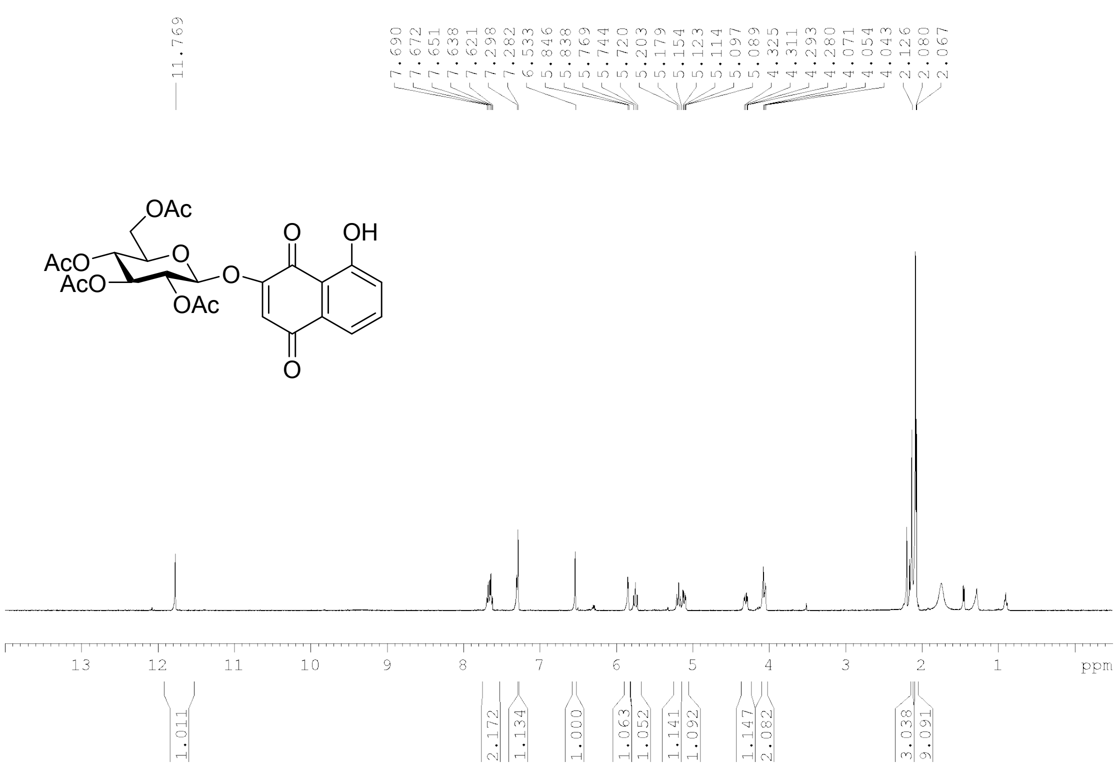

Fig. S63. ^{1}H NMR spectrum of **22** ($CDCl_3$, 400 MHz).

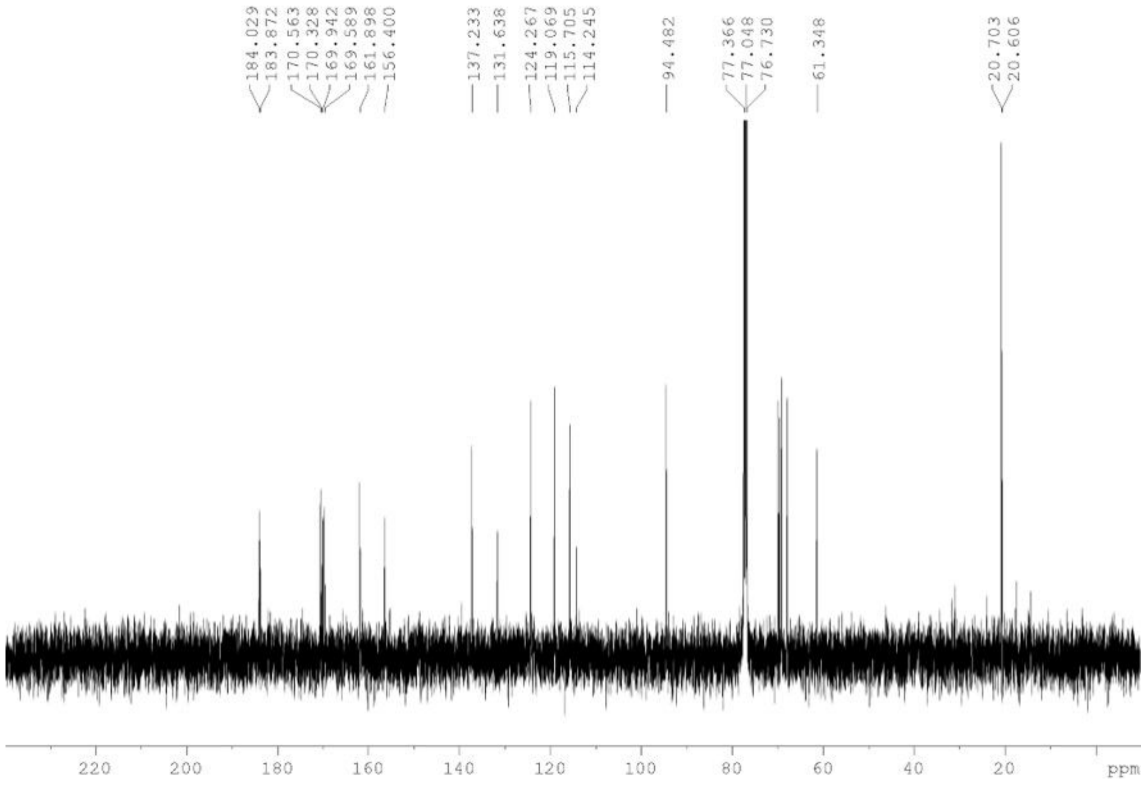

Fig. S64. ^{13}C NMR spectrum of **22** ($CDCl_3$, 100 MHz).

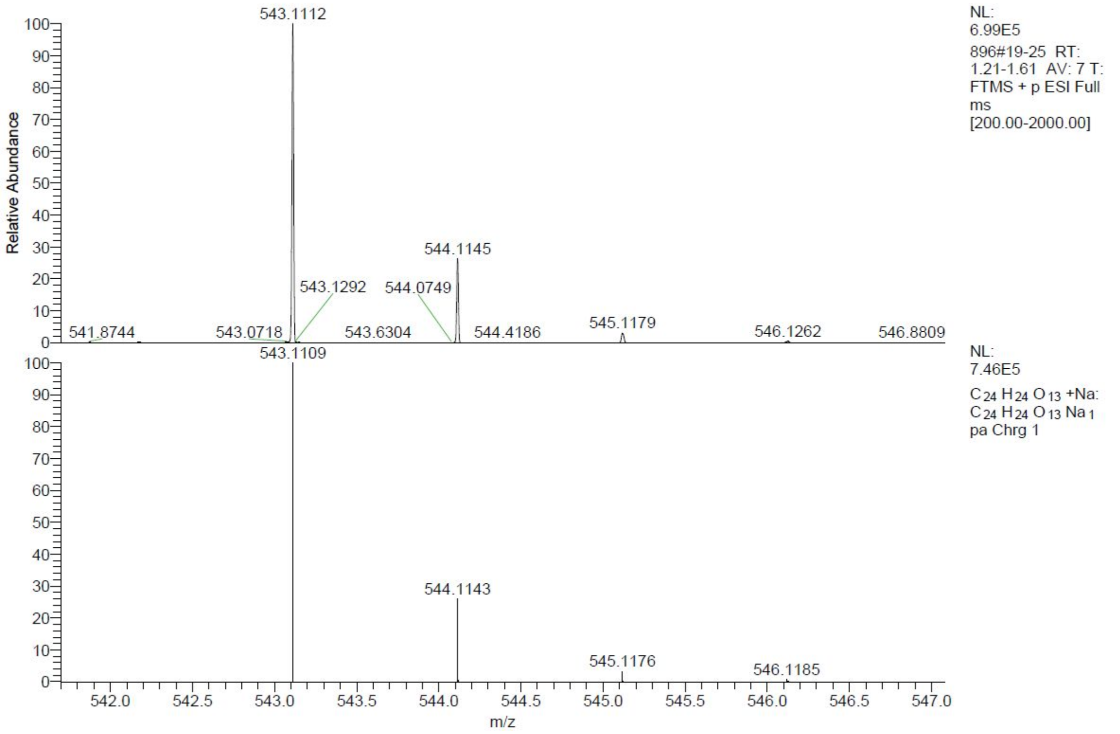

Fig. S65. HR-ESI-MS (+) spectrum of **22.** Upper: found MS; Lower: calculated MS.

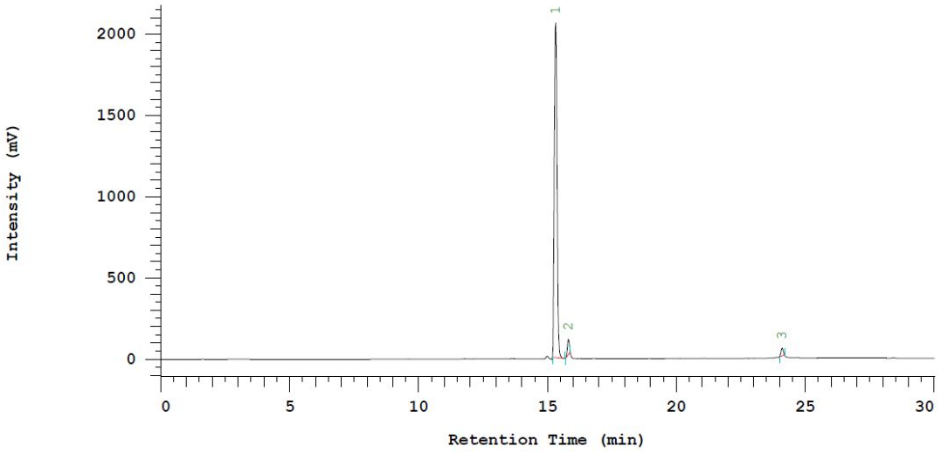

No.	RT	Area	Conc 1	BC
1	15.32	15392203	95.372	BB
2	15.81	470635	2.916	BB
3	24.09	276212	1.711	BB
		16139050	100.000	

Fig. S66. HPLC analysis of **22**.

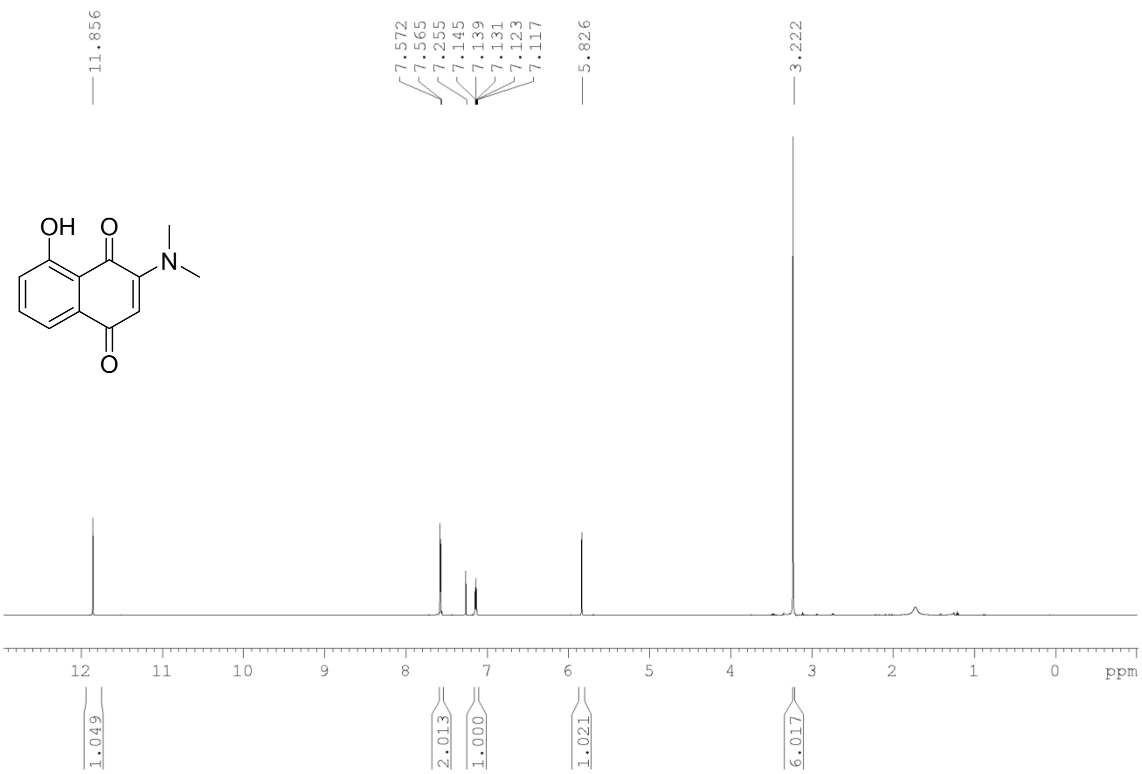

Fig. S67. ^{1}H NMR spectrum of **23** ($CDCl_3$, 600 MHz).

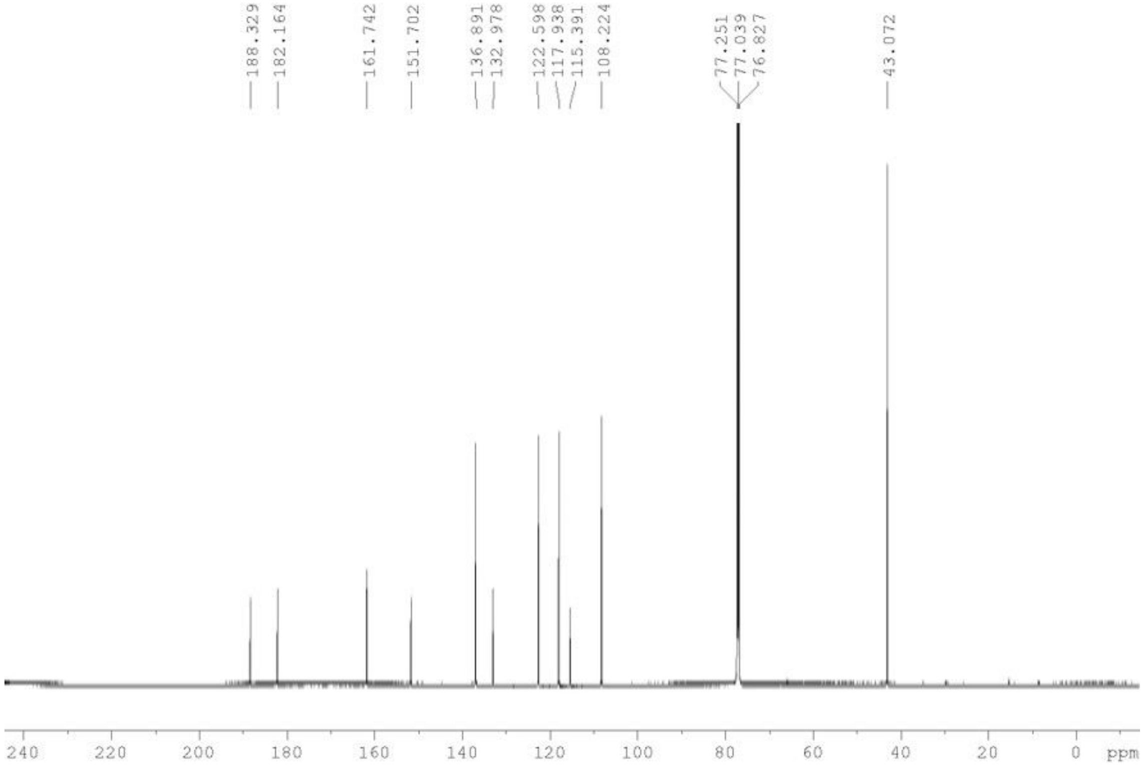

Fig. S68. ^{13}C NMR spectrum of **23** ($CDCl_3$, 150 MHz).

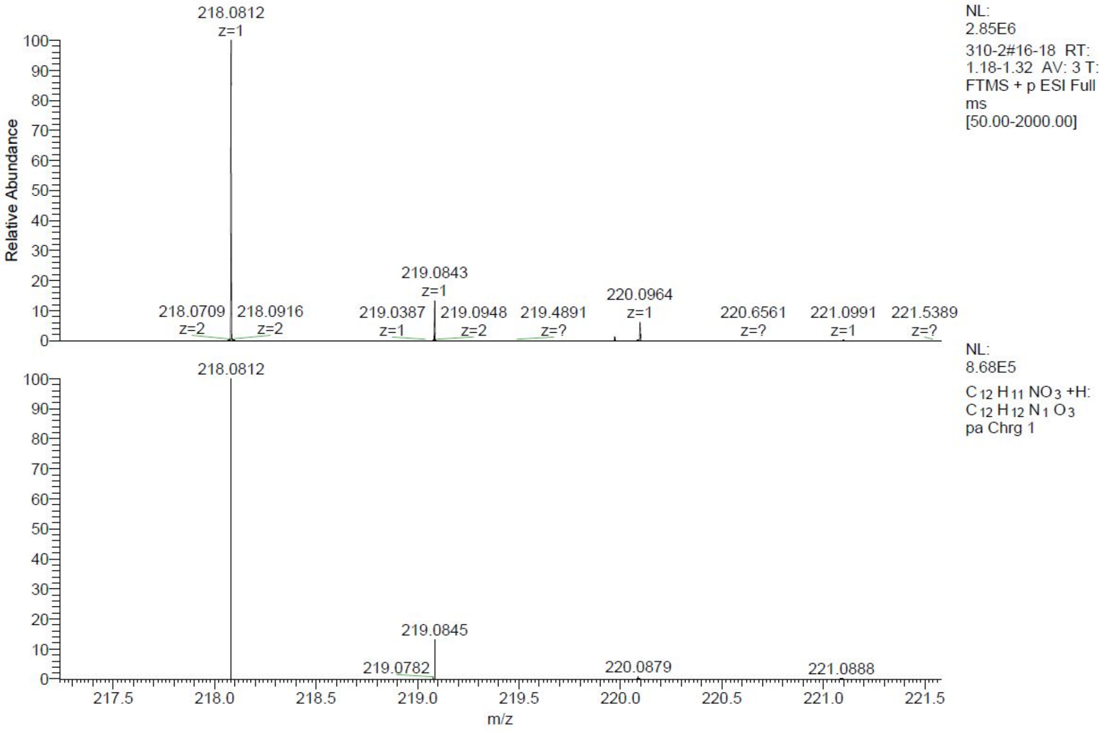

Fig. S69. HR-ESI-MS (+) spectrum of **23.** Upper: found MS; Lower: calculated MS.

<Chromatogram>

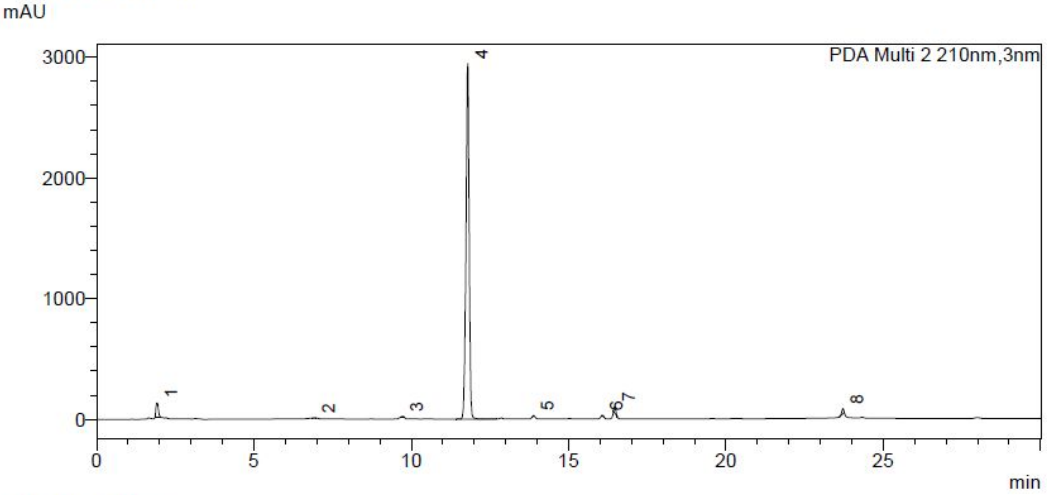

<Peak Table>

PDA Ch2 210nm

Peak#	Ret. Time	Area	Height	Conc.	Mark	Area%
1	1.919	627273	118998	2.862	M	2.862
2	6.929	7439	1568	0.034	M	0.034
3	9.730	7144	3016	0.033	M	0.033
4	11.796	20862229	2945626	95.178	M	95.178
5	13.883	8012	43	0.037	M	0.037
6	16.073	13545	6272	0.062	M	0.062
7	16.466	201854	53636	0.921	M	0.921
8	23.718	191694	45225	0.875	M	0.875
Total		21919190	3174384			100.000

Fig. S70. HPLC analysis of **23**.

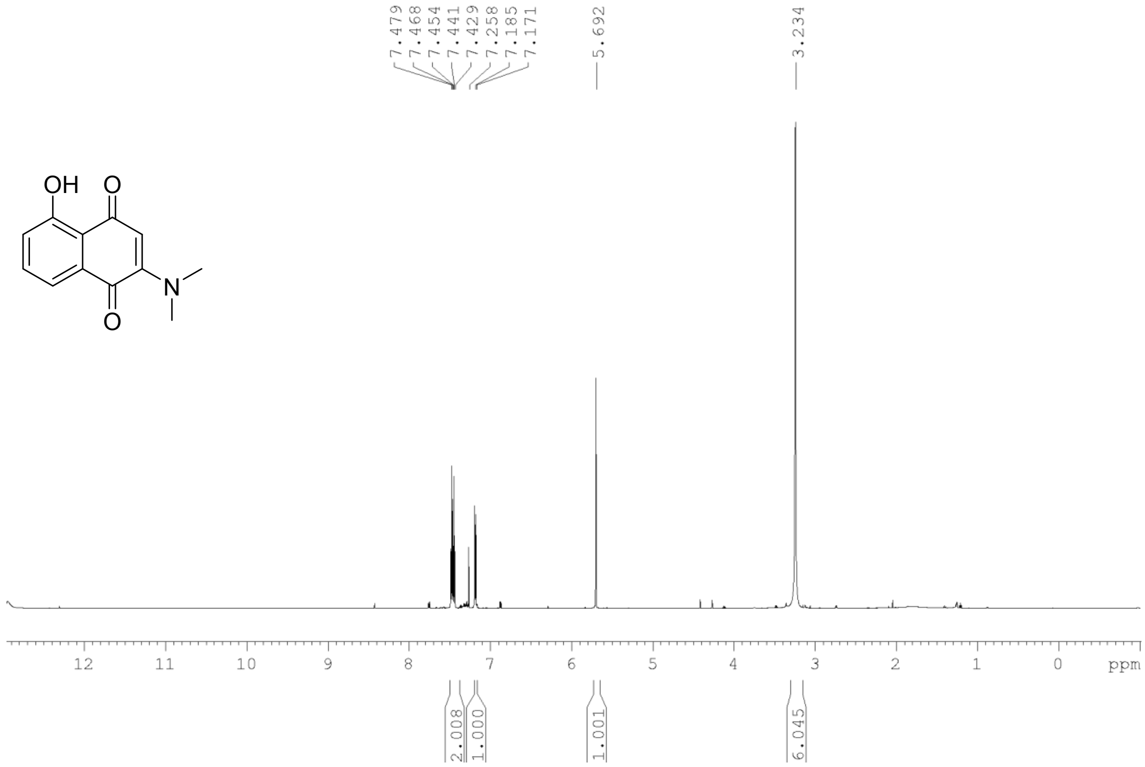

Fig. S71. ^{1}H NMR spectrum of **24** ($CDCl_3$, 600 MHz).

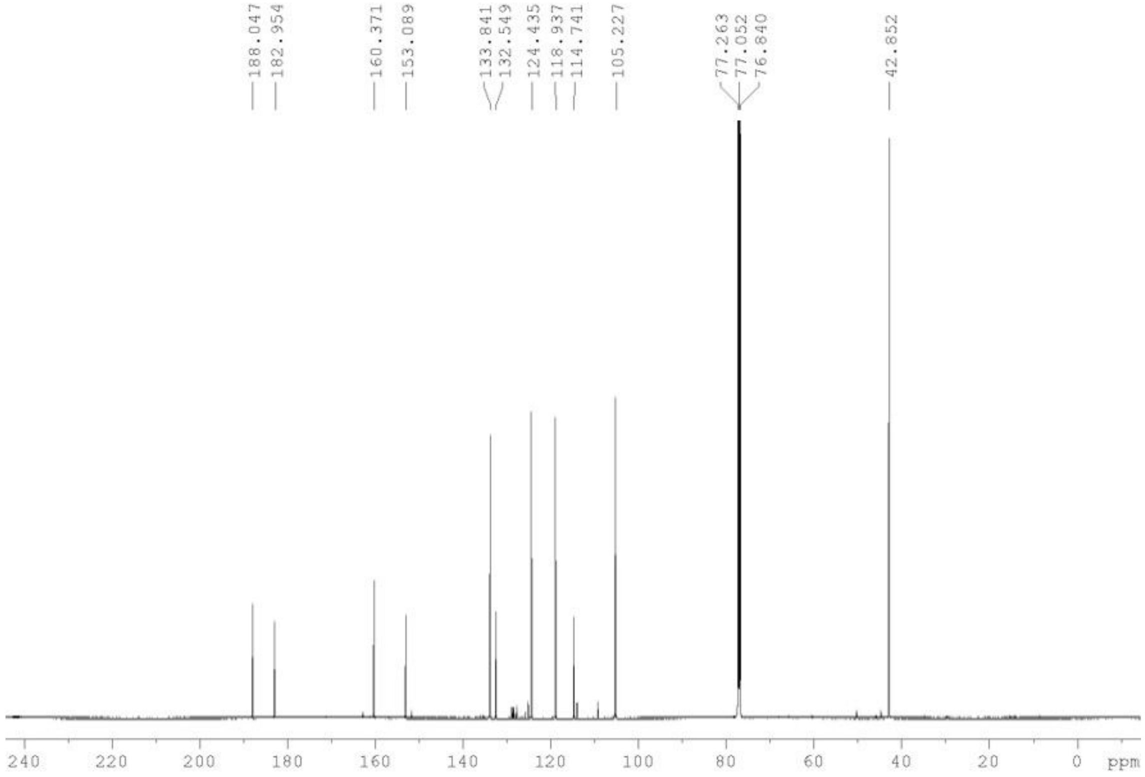

Fig. S72. ^{13}C NMR spectrum of **24** ($CDCl_3$, 150 MHz).

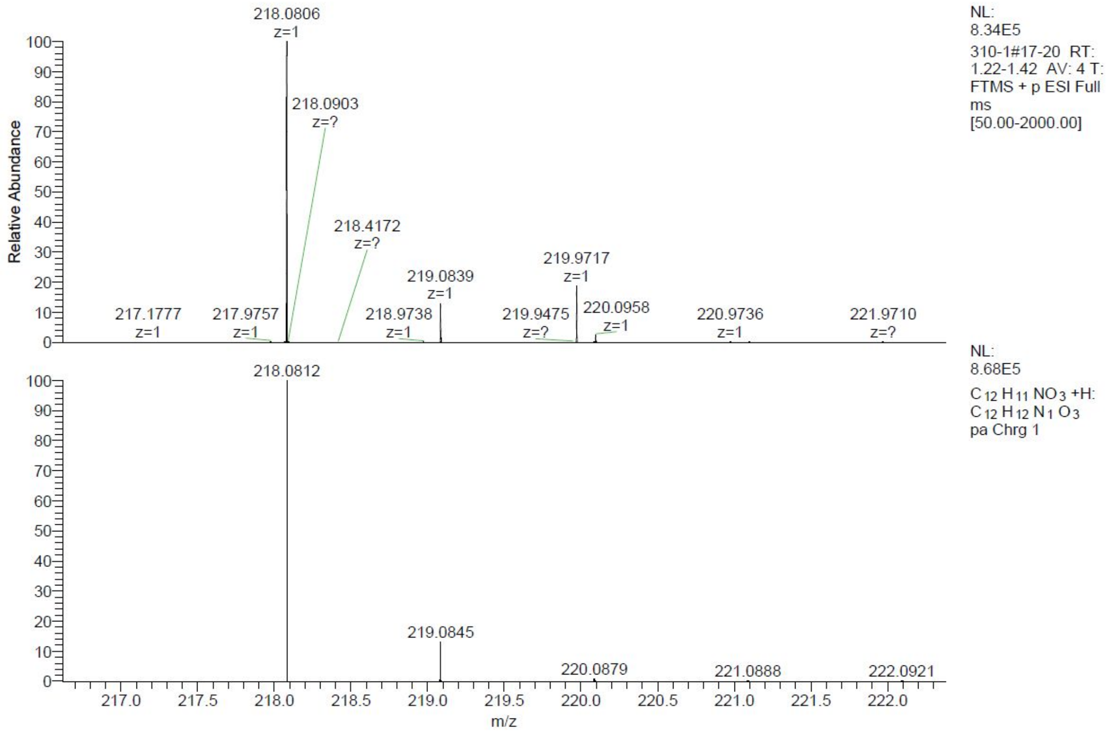

Fig. S73. HR-ESI-MS (+) spectrum of **24.** Upper: found MS; Lower: calculated MS.

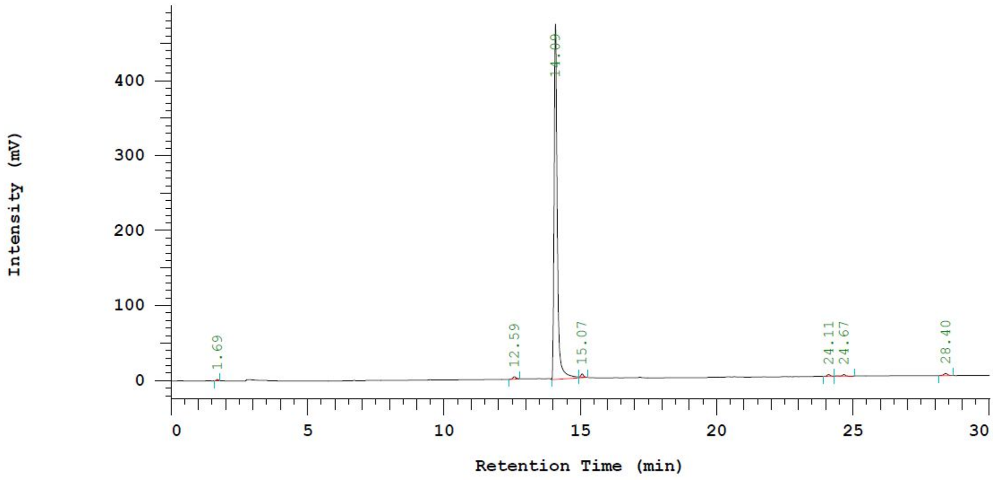

No.	RT	Area	Conc 1	BC
1	1.69	8785	0.223	BB
2	12.59	23933	0.607	BB
3	14.09	3817030	96.811	BB
4	15.07	28736	0.729	TBB
5	24.11	16609	0.421	BB
6	24.67	18866	0.479	BB
7	28.40	28817	0.731	BB
		3942776	100.000	

Fig. S74. HPLC analysis of **24**.

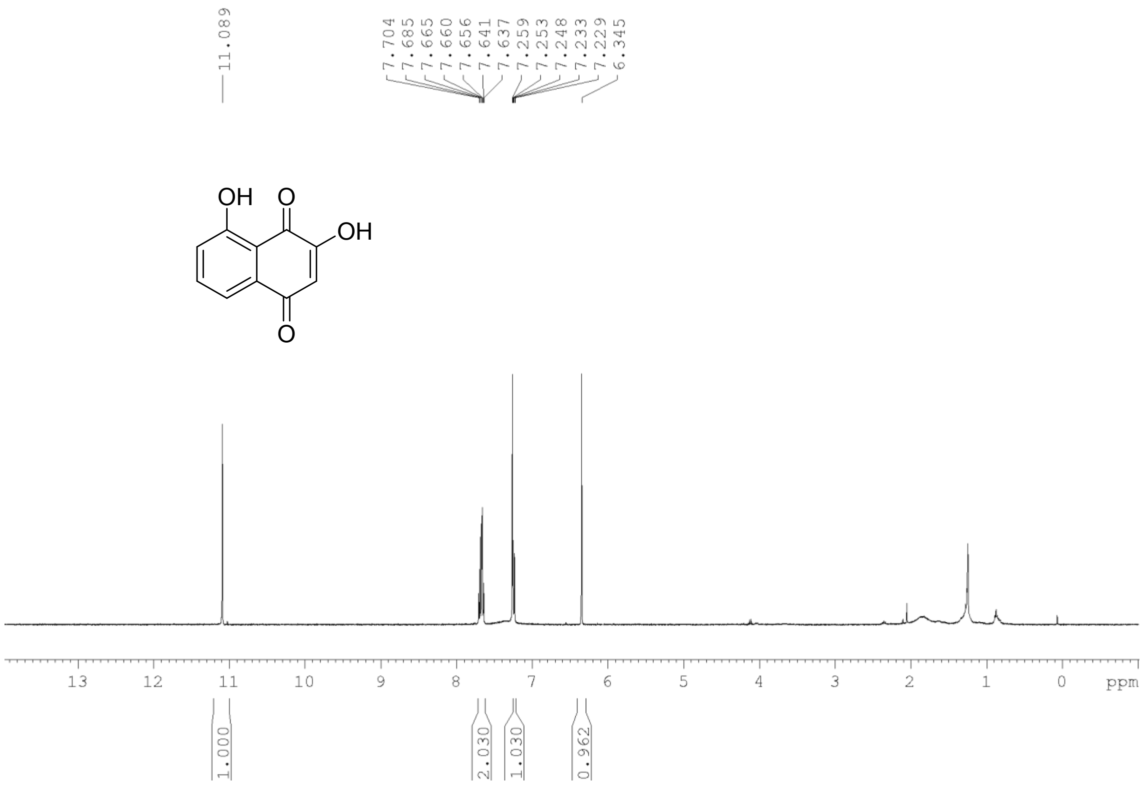

Fig. S75. ^{1}H NMR spectrum of **25** ($CDCl_3$, 400 MHz).

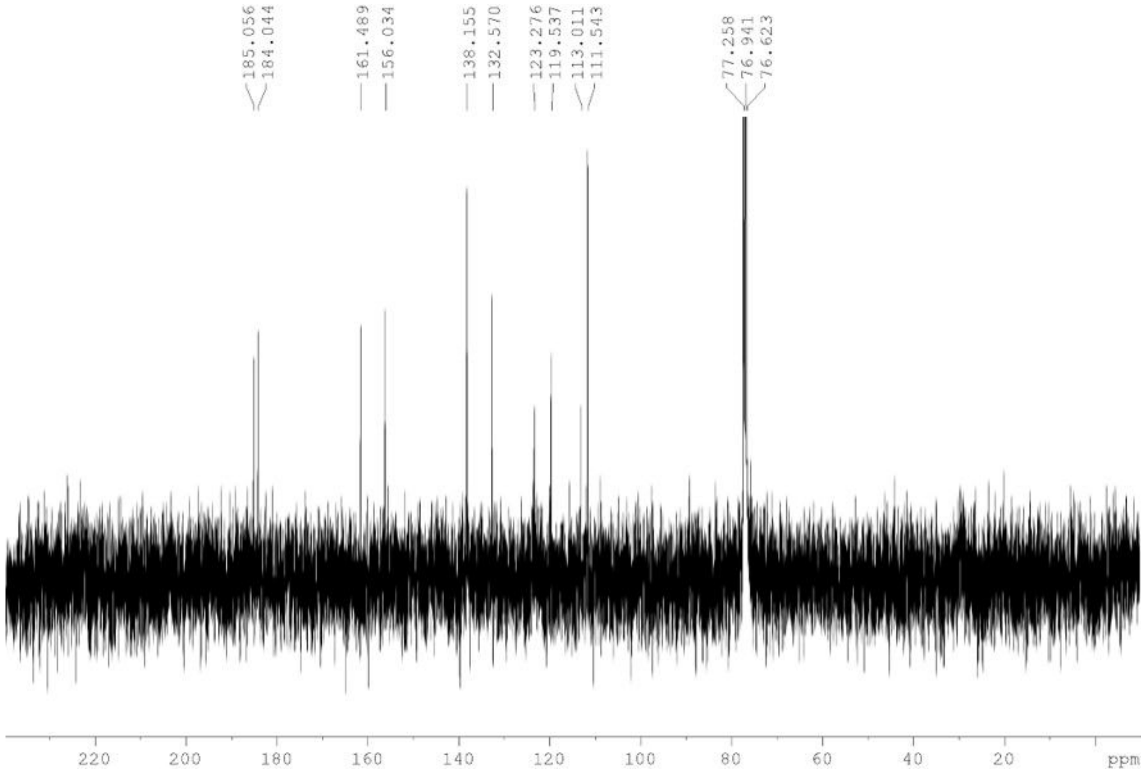

Fig. S76. ^{13}C NMR spectrum of **25** ($CDCl_3$, 100 MHz).

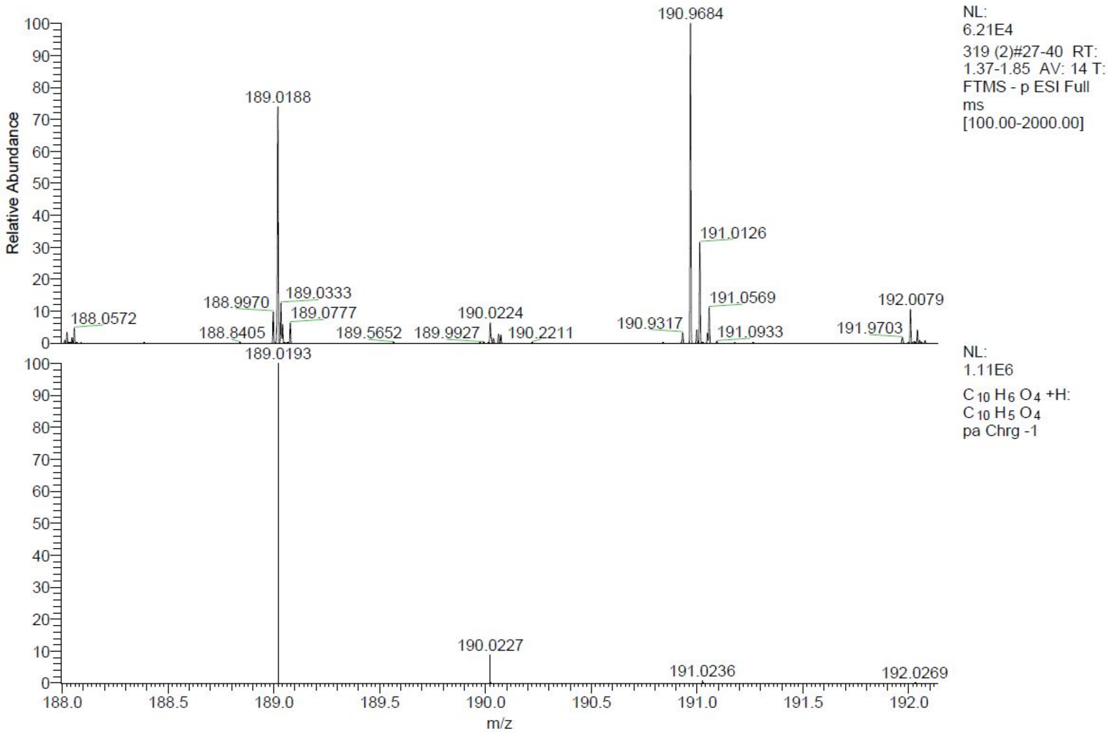

Fig. S77. HR-ESI-MS (+) spectrum of **25.** Upper: found MS; Lower: calculated MS.

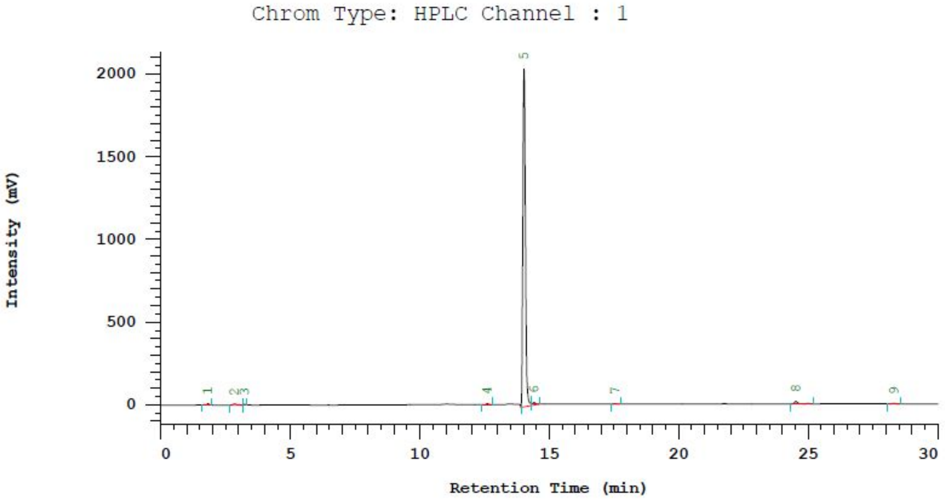

No.	RT	Area	Conc 1	BC
1	1.83	48184	0.331	BB
2	2.84	65754	0.452	BB
3	3.23	15387	0.106	BB
4	12.60	31928	0.220	BB
5	14.03	14115491	97.074	BV
6	14.41	47269	0.325	TBB
7	17.55	23178	0.159	BB
8	24.52	153821	1.058	BB
9	28.31	39883	0.274	BB
		14540895	100.000	

Fig. S78. HPLC analysis of **25**.

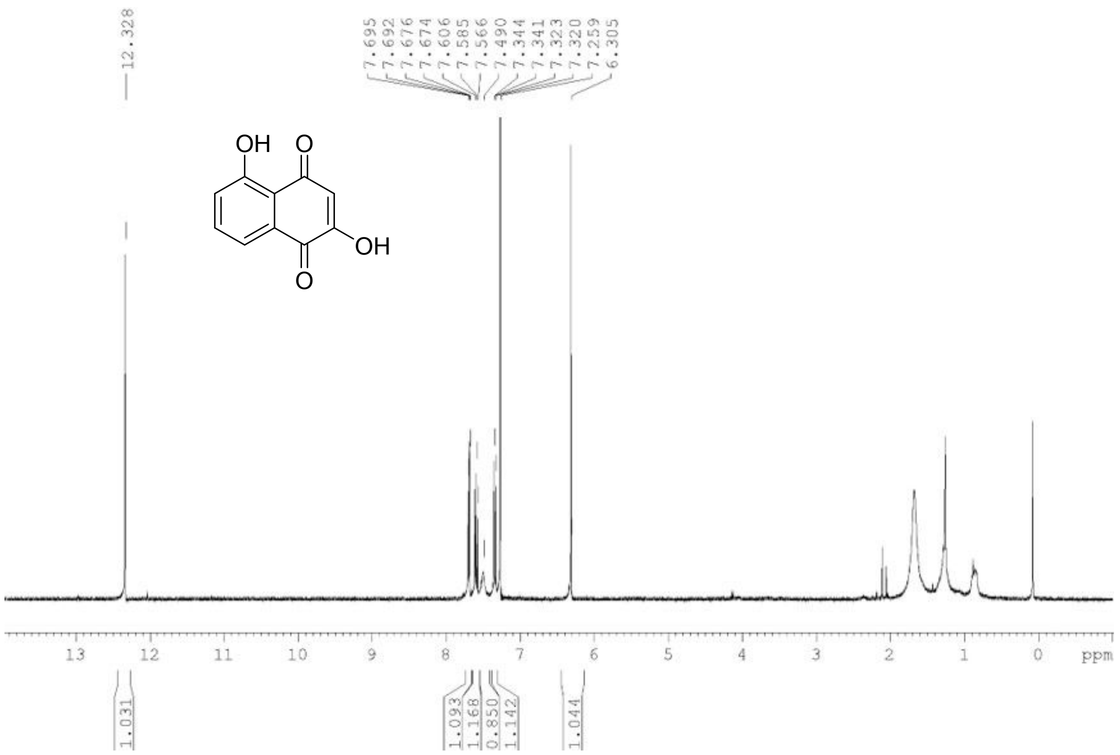

Fig. S79. ^{1}H NMR spectrum of **26** ($CDCl_3$, 400 MHz).

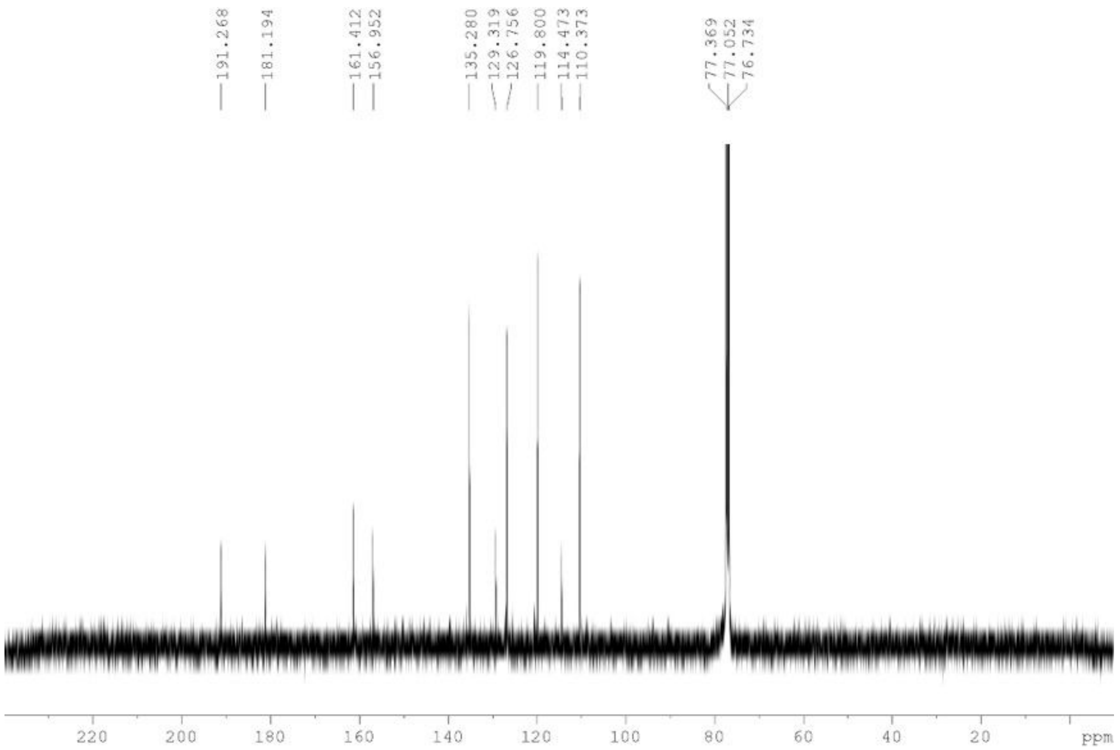

Fig. S80. ^{13}C NMR spectrum of **26** ($CDCl_3$, 100 MHz).

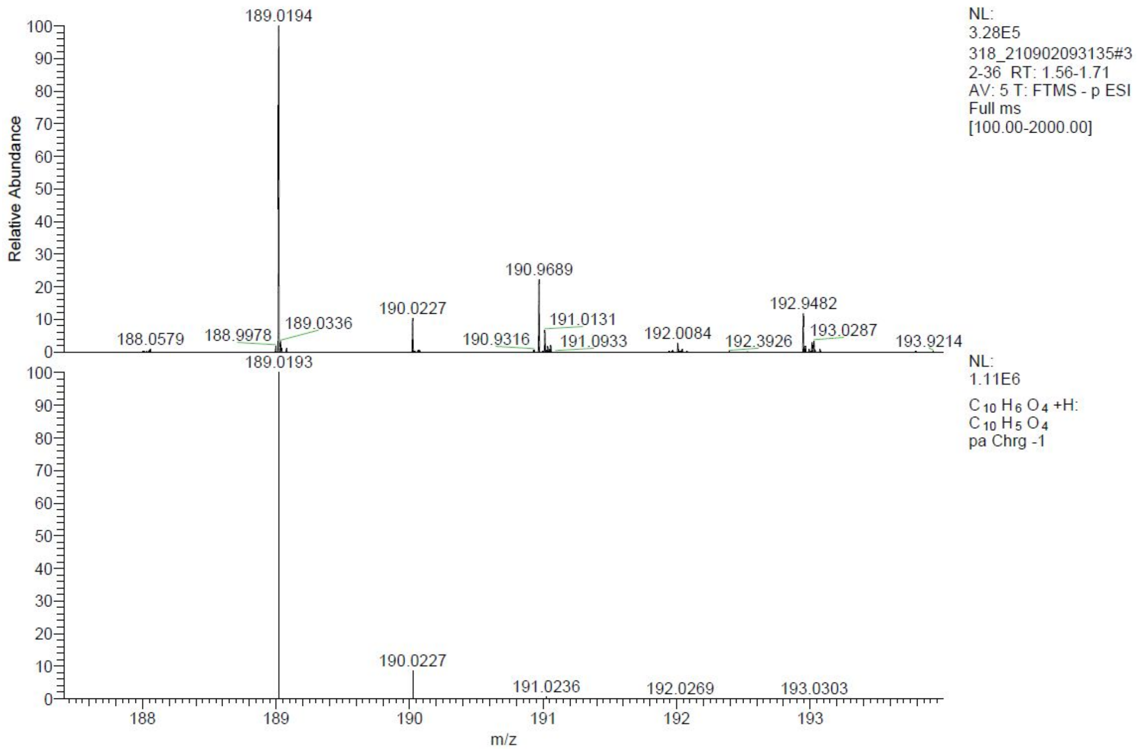

Fig. S81. HR-ESI-MS (+) spectrum of **26.** Upper: found MS; Lower: calculated MS.

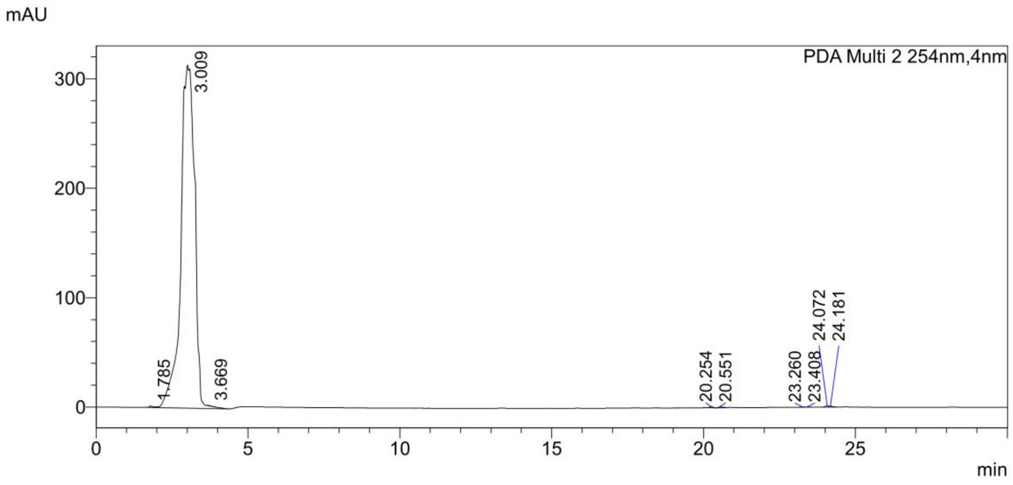

<Peak Table>

PDA Ch2 254nm

Peak#	Ret. Time	Area	Height	Conc.	Unit	Mark	Name
1	1.785	14438	1484	0.144			
2	3.009	9987275	313474	99.514		SV	
3	3.669	3031	330	0.030		T	
4	20.254	6719	973	0.067			
5	20.551	7289	970	0.073			
6	23.260	2205	407	0.022			
7	23.408	1837	328	0.018		V	
8	24.072	7439	1279	0.074			
9	24.181	5855	970	0.058		V	
Total		10036089	320215				

Fig. S82. HPLC analysis of **26**.

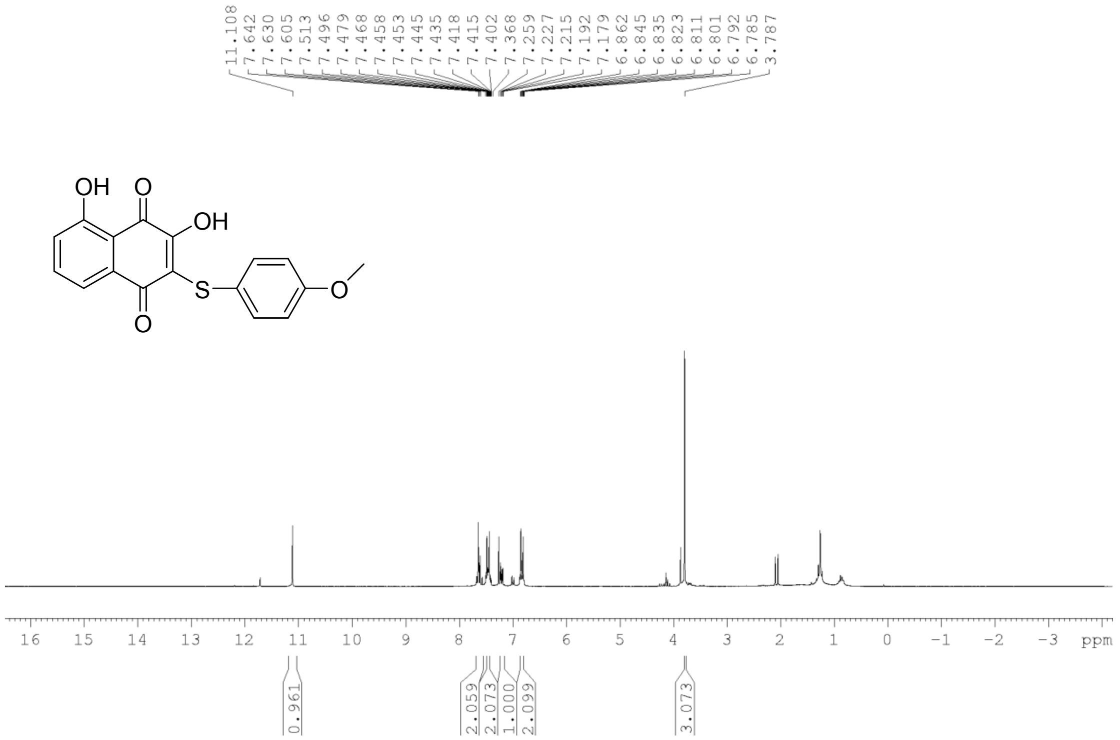

Fig. S83. ^{1}H NMR spectrum of **27** ($CDCl_3$, 400 MHz).

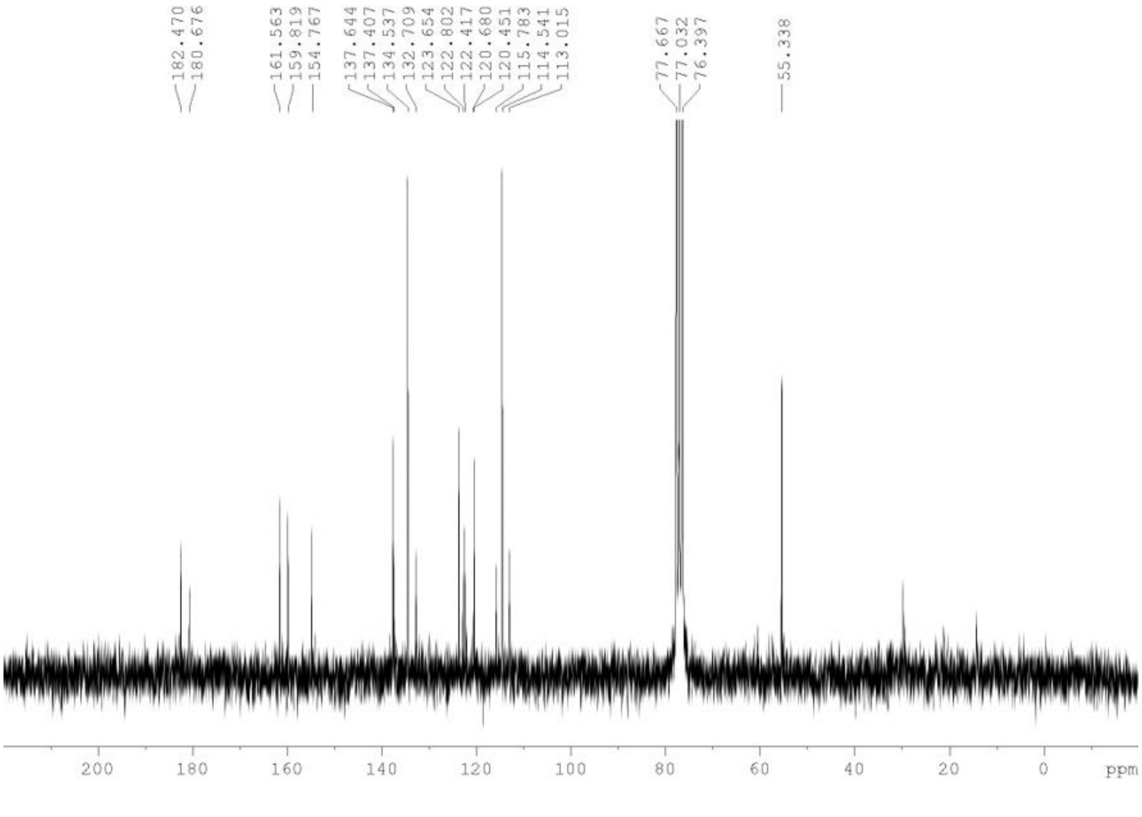

Fig. S84. ^{13}C NMR spectrum of **27** ($CDCl_3$, 100 MHz).

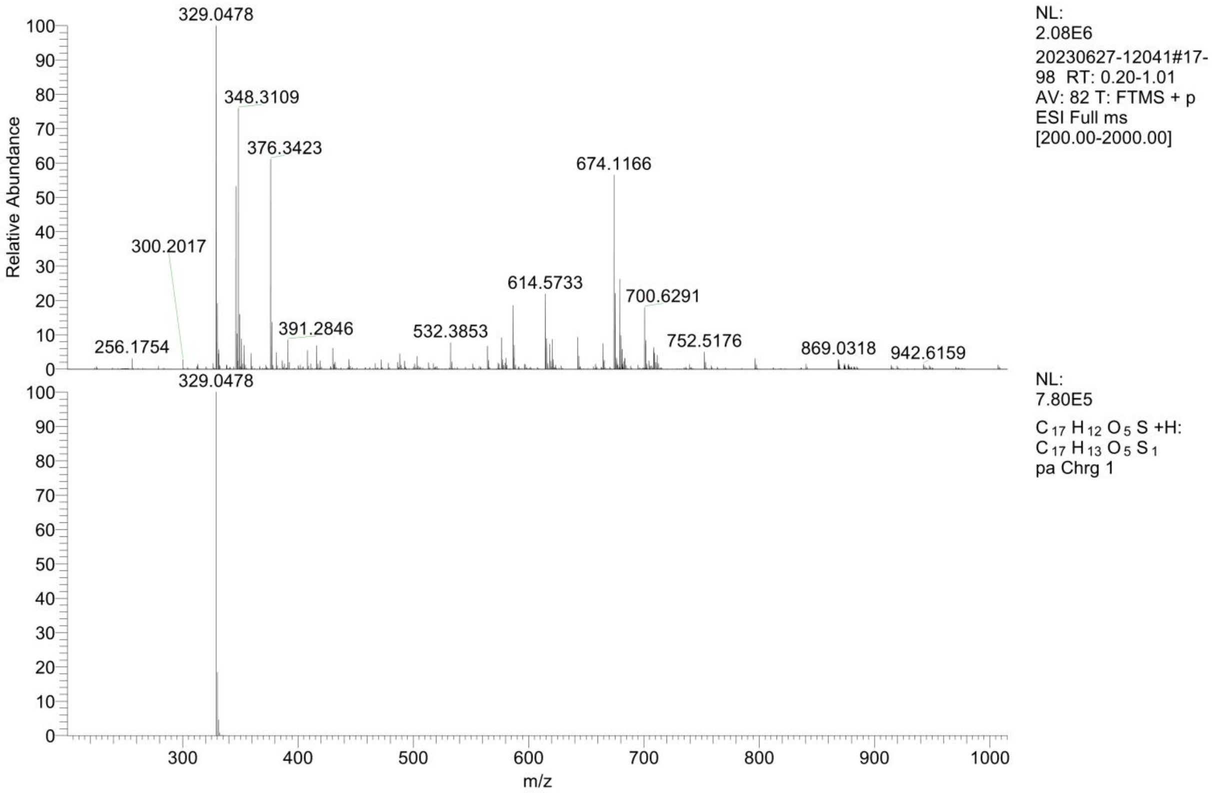

Fig. S85. HR-ESI-MS (+) spectrum of **27.** Upper: found MS; Lower: calculated MS.

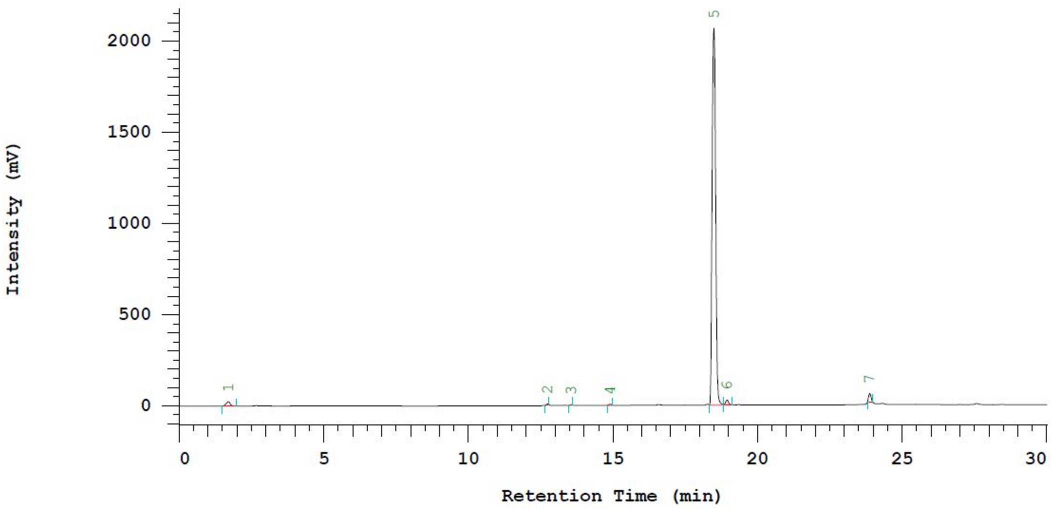

No.	RT	Area	Conc 1	BC
1	1.71	230352	1.262	BB
2	12.75	21114	0.116	BB
3	13.56	3870	0.021	BB
4	14.91	38227	0.209	BB
5	18.49	17508320	95.949	BV
6	18.95	168756	0.925	TBB
7	23.88	276804	1.517	BB
		18247443	100.000	

Fig. S86. HPLC analysis of **27**.

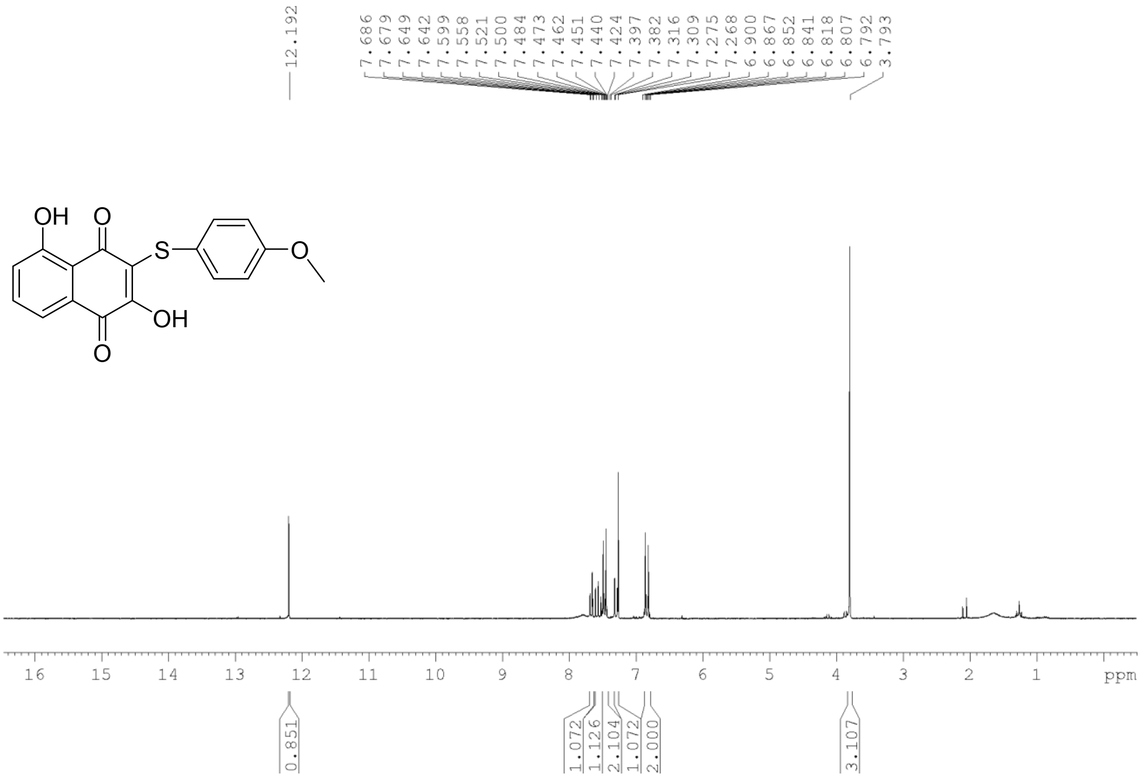

Fig. S87. ^{1}H NMR spectrum of **28** ($CDCl_3$, 400 MHz).

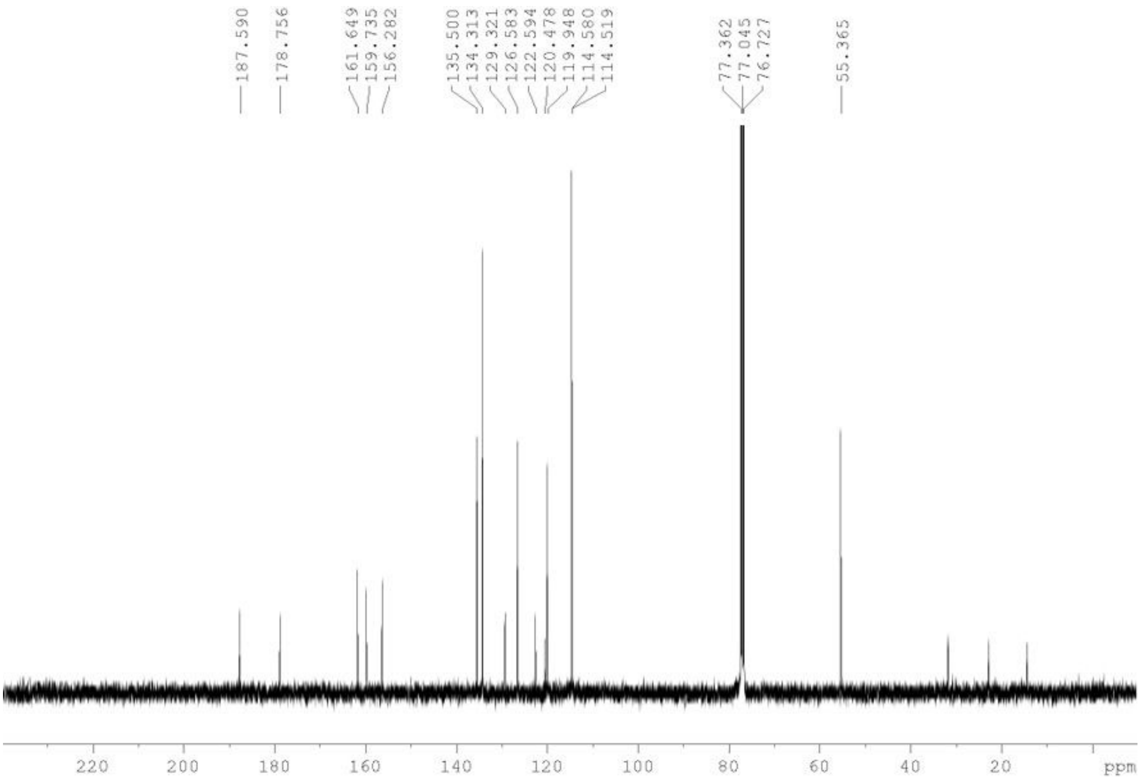

Fig. S88. ^{13}C NMR spectrum of **28** ($CDCl_3$, 100 MHz).

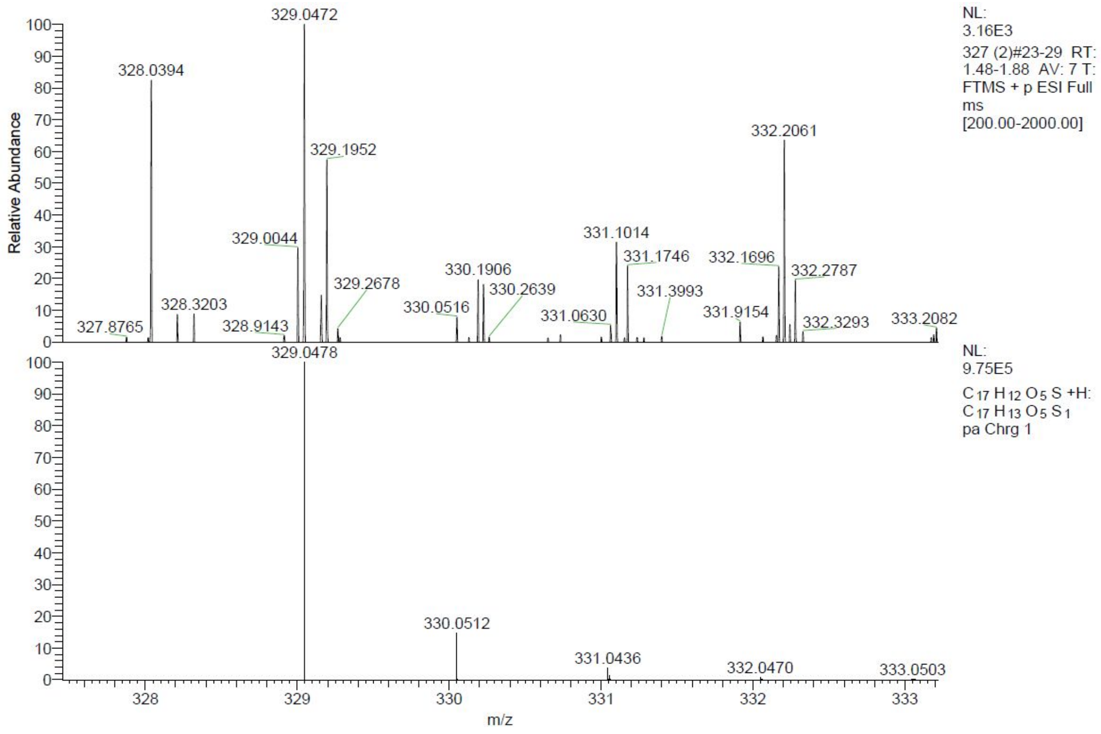

Fig. S89. HR-ESI-MS (+) spectrum of **28.** Upper: found MS; Lower: calculated MS.

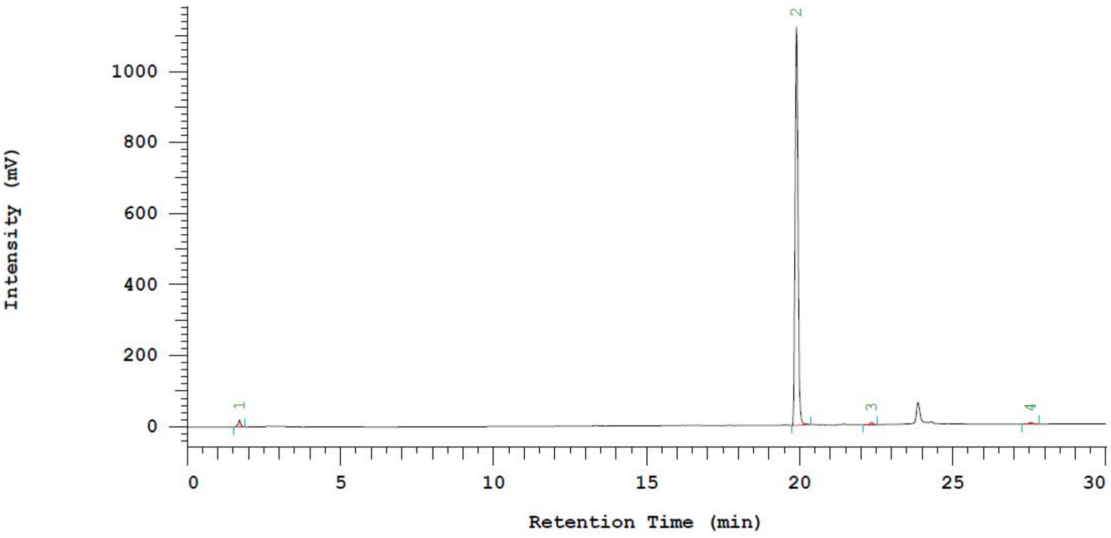

No.	RT	Area	Conc 1	BC
1	1.71	116836	1.504	BB
2	19.89	7558030	97.305	BB
3	22.35	45960	0.592	BB
4	27.55	46521	0.599	BB
		7767347	100.000	

Fig. S90. HPLC analysis of **28**.

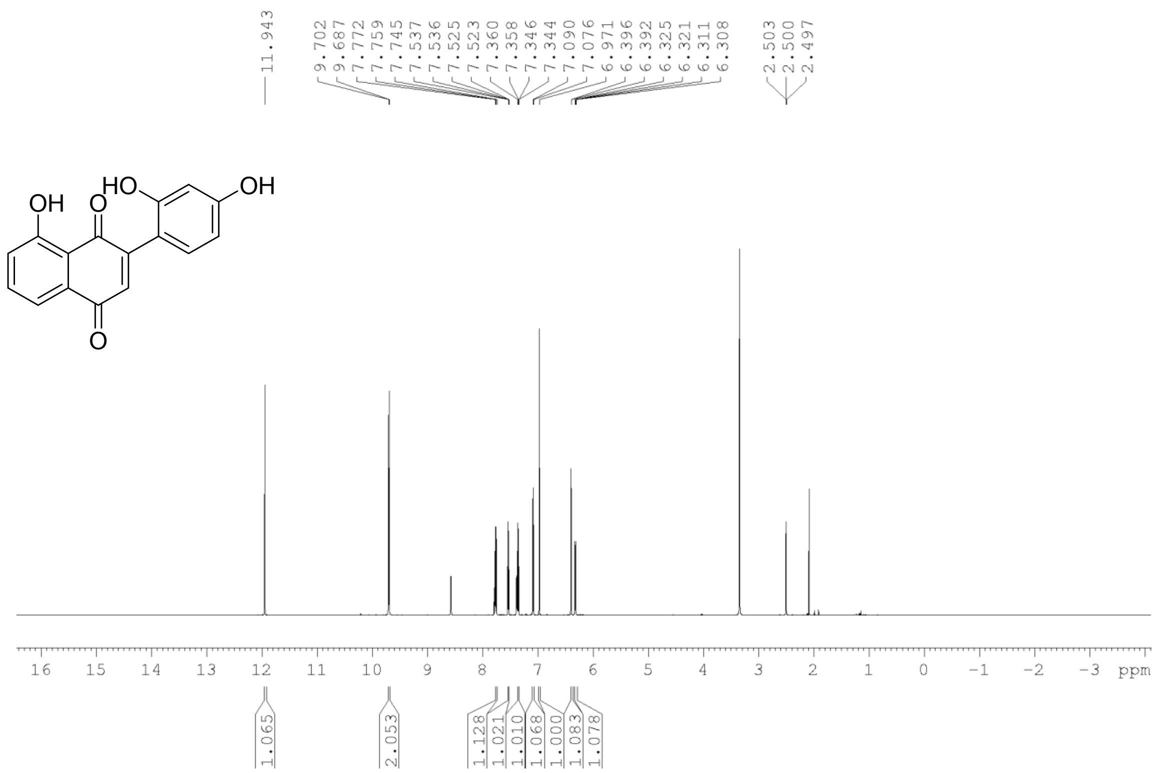

Fig. S91. ^{1}H NMR spectrum of **29** ($(CD_3)_2SO$, 600 MHz).

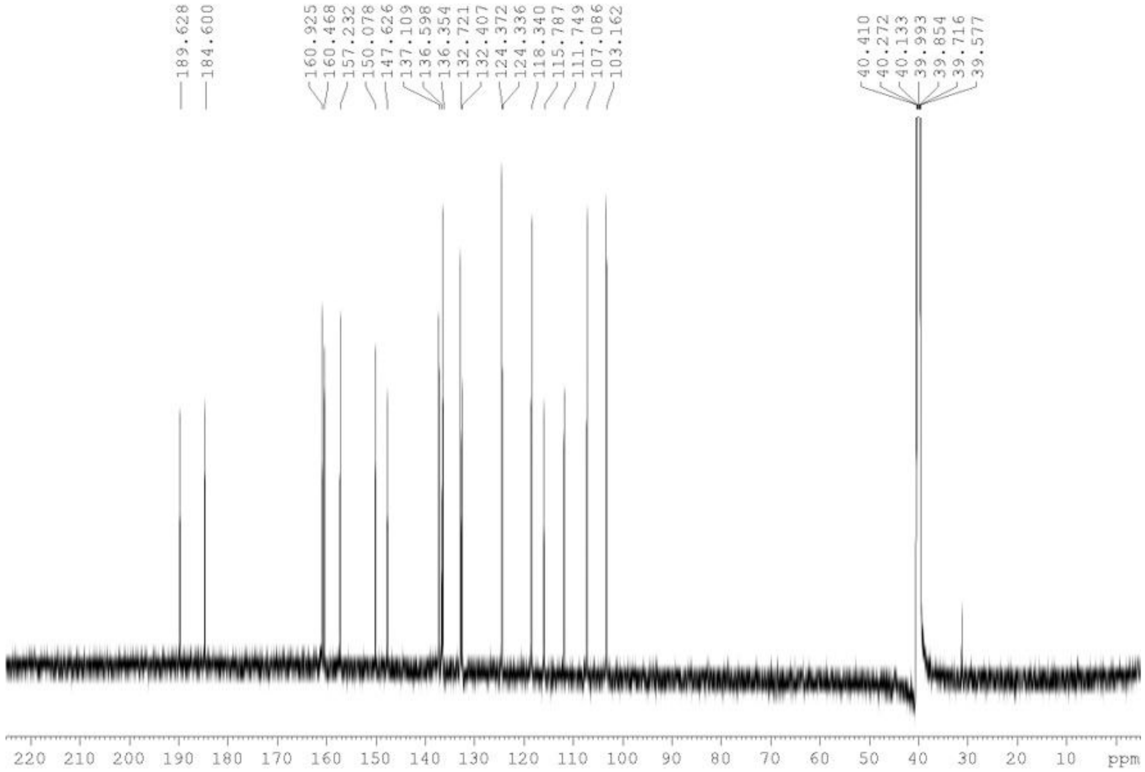

Fig. S92. ^{13}C NMR spectrum of **29** ($(CD_3)_2SO$, 150 MHz).

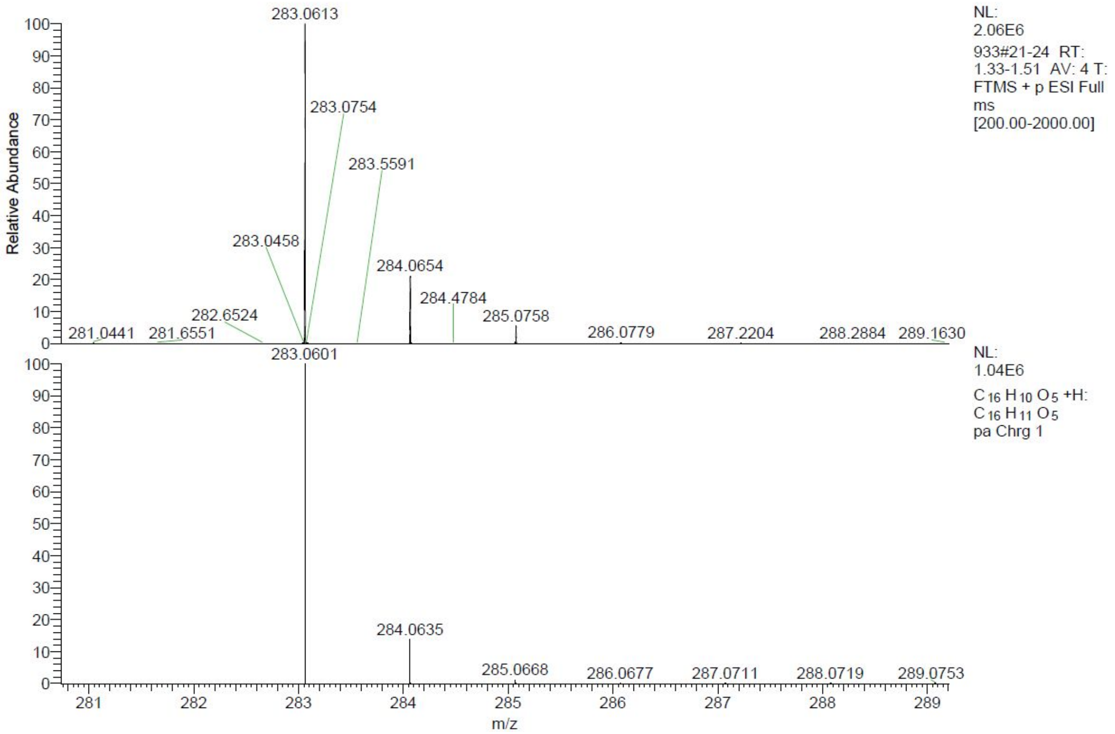

Fig. S93. HR-ESI-MS (+) spectrum of **29.** Upper: found MS; Lower: calculated MS.

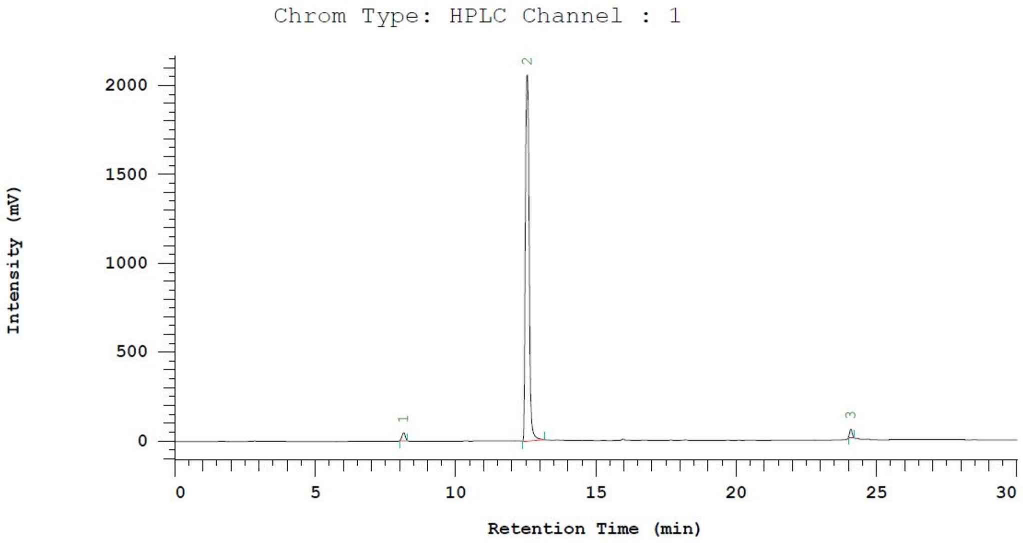

No.	RT	Area	Conc 1	BC
1	8.15	366302	1.841	BB
2	12.56	19257552	96.766	BB
3	24.08	277245	1.393	BB
		19901099	100.000	

Fig. S94. HPLC analysis of **29**.

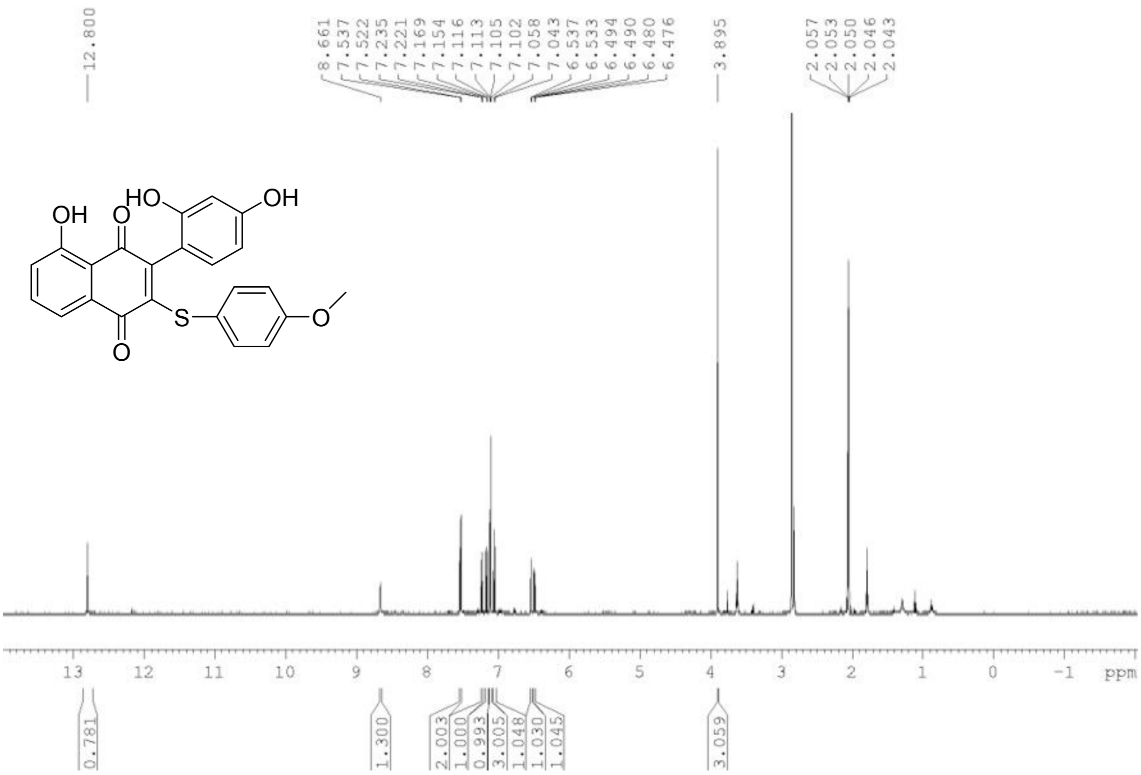

Fig. S95. ^{1}H NMR spectrum of **30** ($(CD_3)_2CO$, 600 MHz).

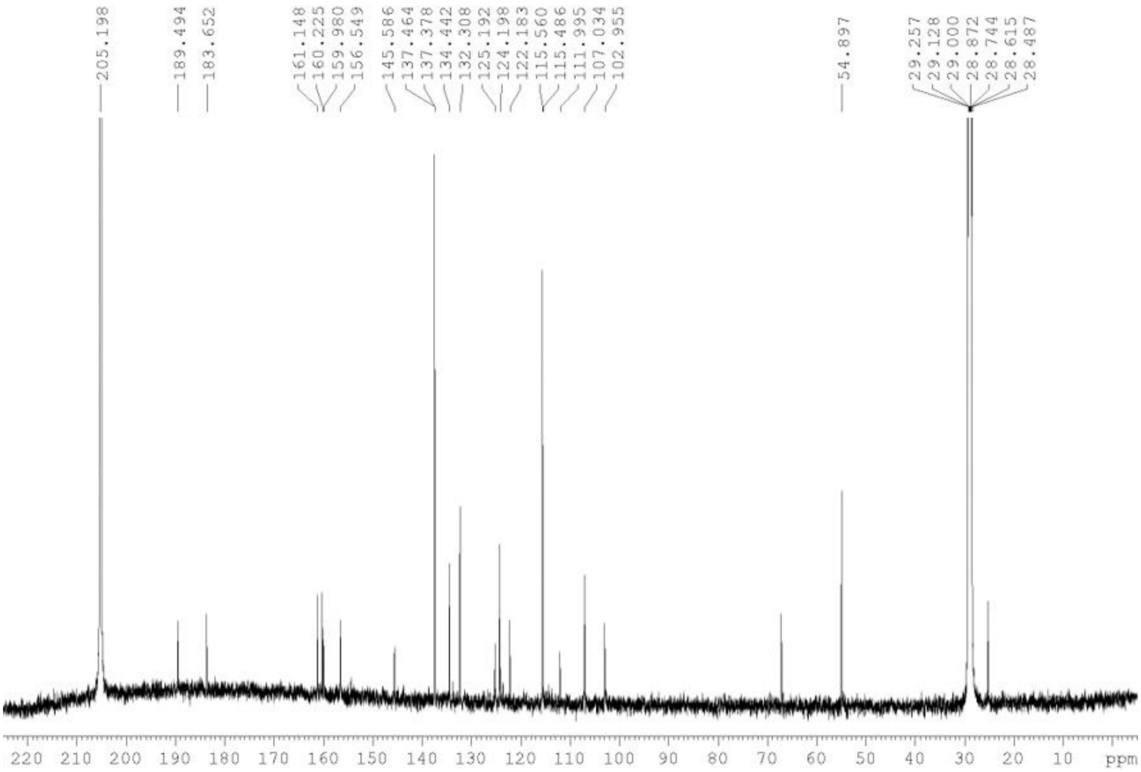

Fig. S96. ^{13}C NMR spectrum of **30** ($(CD_3)_2CO$, 150 MHz).

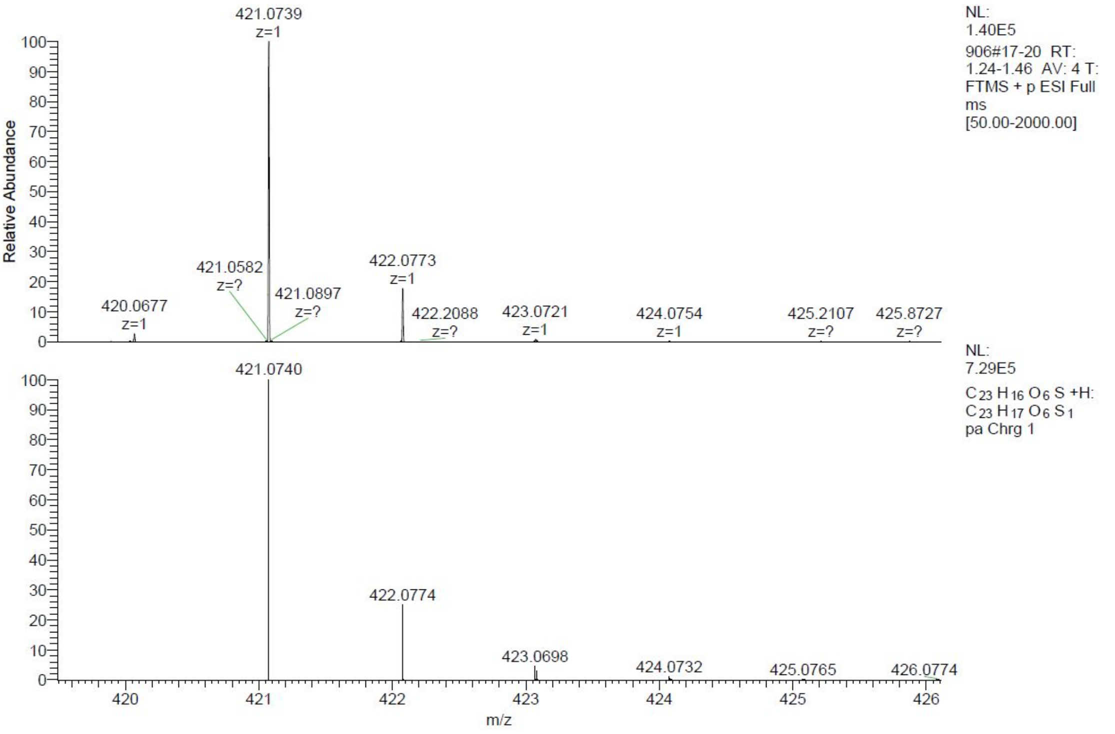

Fig. S97. HR-ESI-MS (+) spectrum of **30.** Upper: found MS; Lower: calculated MS.

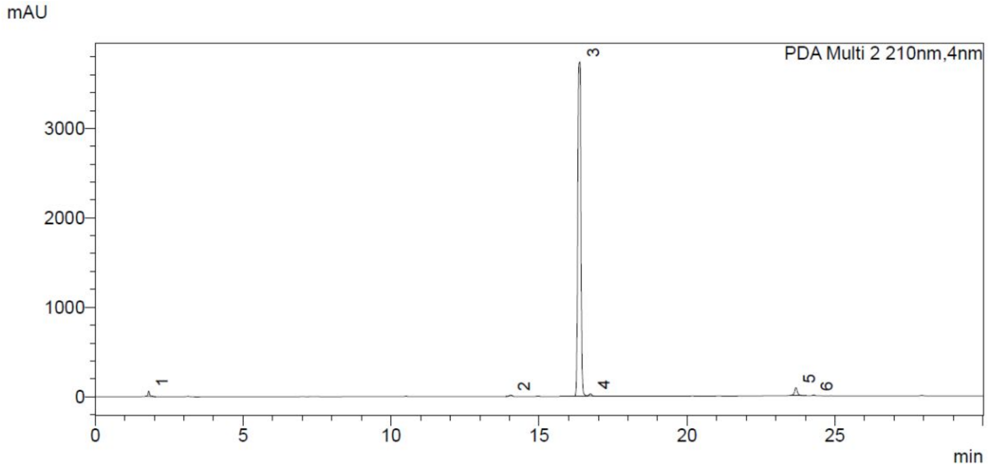

<Peak Table>

PDA Ch2 210nm

Peak#	Ret. Time	Area	Height	Conc.	Mark	Area%
1	1.811	215083	58833	0.000	M	0.754
2	14.043	29951	7780	0.000	M	0.105
3	16.379	27572810	3739716	0.000		96.711
4	16.737	128365	24137	0.000	M	0.450
5	23.680	562727	85664	0.000	M	1.974
6	24.281	1701	975	0.000	M	0.006
Total		28510638	3917105			100.000

Fig. S98. HPLC analysis of **30**.

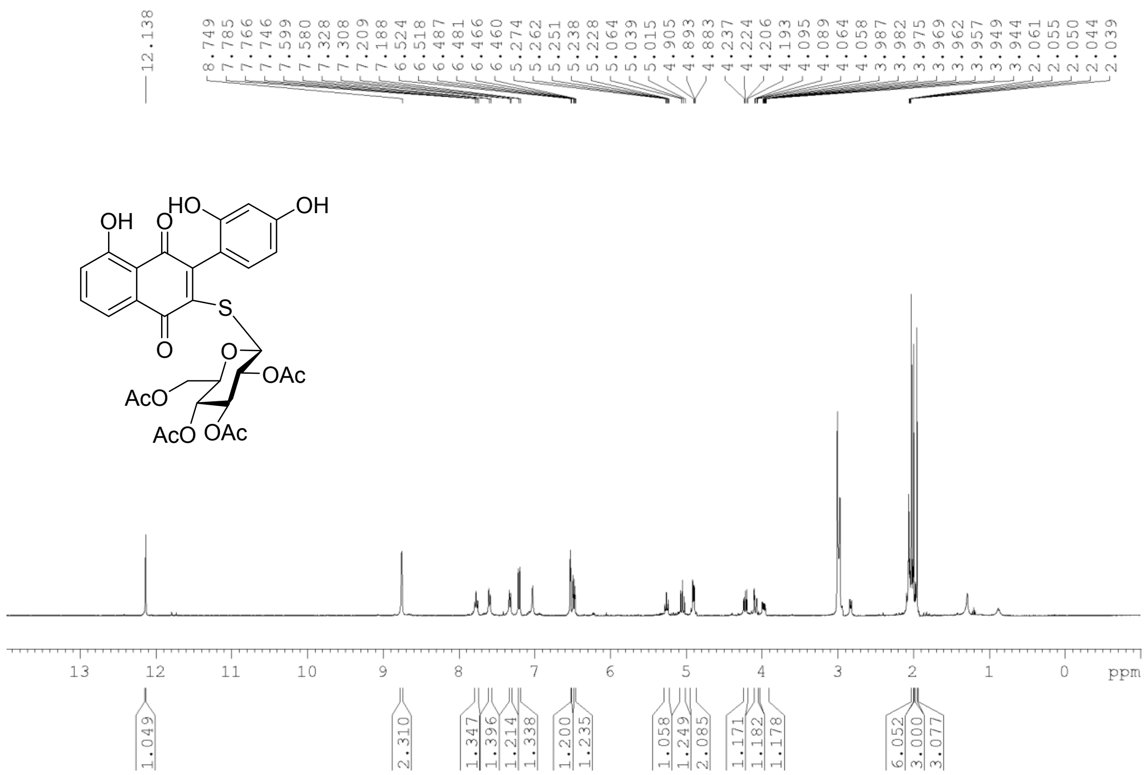

Fig. S99. ^{1}H NMR spectrum of **31** ($(CD_3)_2CO$, 400 MHz).

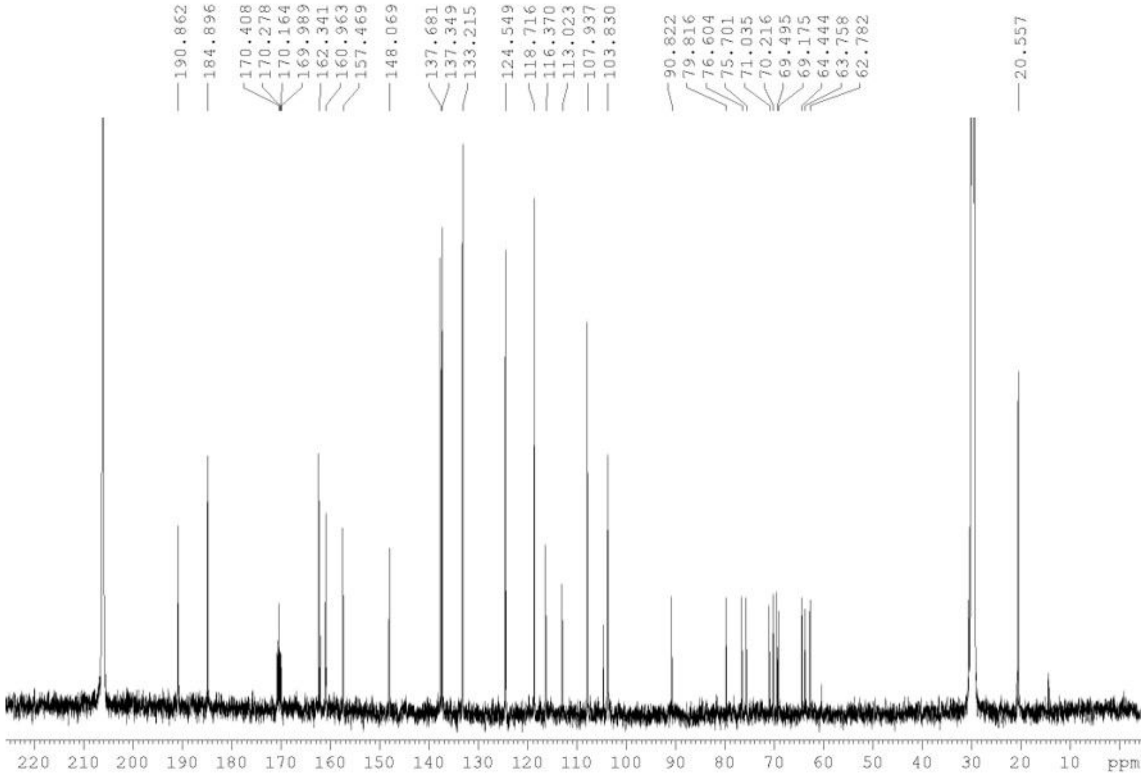

Fig. S100. ^{13}C NMR spectrum of **31** ($(CD_3)_2CO$, 100 MHz).

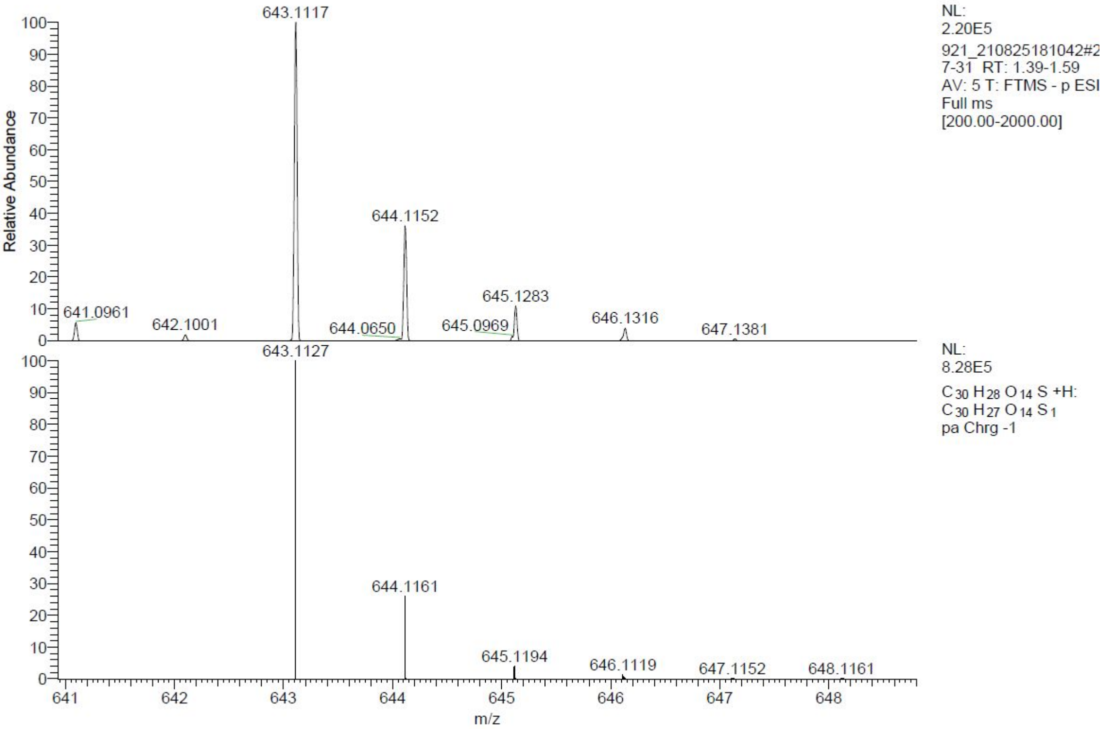

Fig. S101. HR-ESI-MS (+) spectrum of **31.** Upper: found MS; Lower: calculated MS.

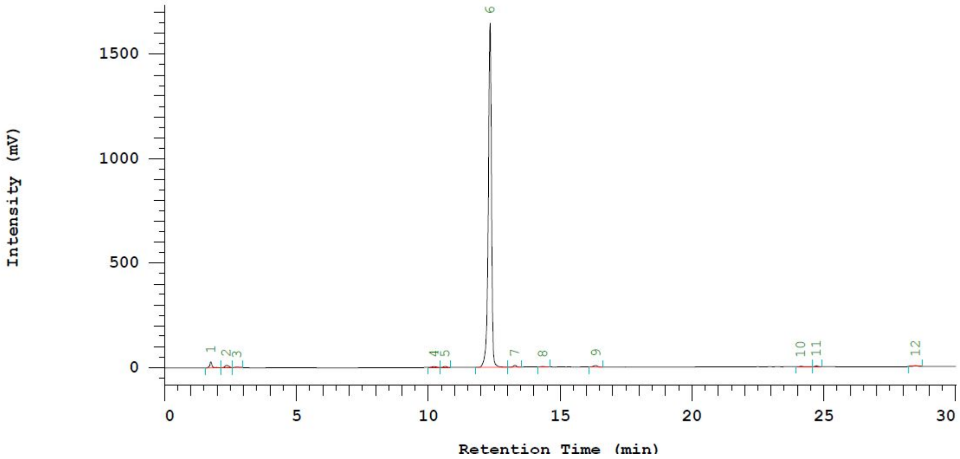

No.	RT	Area	Conc 1	BC
1	1.76	180302	1.238	BB
2	2.35	120687	0.828	BB
3	2.75	27569	0.189	BB
4	10.23	46603	0.320	BB
5	10.65	45866	0.315	BB
6	12.35	13898020	95.394	BB
7	13.29	67881	0.466	BB
8	14.35	22887	0.157	BB
9	16.36	75263	0.517	BB
10	24.13	15590	0.107	BB
11	24.72	27468	0.189	BB
12	28.48	40895	0.281	BB
		14569031	100.000	

Fig. S102. HPLC analysis of **31**.
